# Journey on VX-809-Based Hybrid Derivatives towards Drug-like F508del-CFTR Correctors: From Molecular Modeling to Chemical Synthesis and Biological Assays

**DOI:** 10.3390/ph15030274

**Published:** 2022-02-23

**Authors:** Alice Parodi, Giada Righetti, Emanuela Pesce, Annalisa Salis, Valeria Tomati, Cristina Pastorino, Bruno Tasso, Mirko Benvenuti, Gianluca Damonte, Nicoletta Pedemonte, Elena Cichero, Enrico Millo

**Affiliations:** 1 Department of Experimental Medicine, Section of Biochemistry, University of Genoa, Viale Benedetto XV, 1, 16132 Genoa, Italy; alice.parodi1994@gmail.com (A.P.); annalisa.salis@unige.it (A.S.); mirko.benvenuti@edu.unige.it (M.B.); gianluca.damonte@unige.it (G.D.); 2 Department of Pharmacy, Section of Medicinal Chemistry, School of Medical and Pharmaceutical Sciences, University of Genoa, Viale Benedetto XV, 3, 16132 Genoa, Italy; righetti@difar.unige.it (G.R.); tasso@difar.unige.it (B.T.); 3 UOC Genetica Medica, IRCCS Istituto Giannina Gaslini, 16147 Genoa, Italy; emanuela.pesce@yahoo.it (E.P.); valeriatomati@gaslini.org (V.T.); nicolettapedemonte@gaslini.org (N.P.); 4 Department of Neurosciences, Rehabilitation, Ophthalmology, Genetics, Maternal and Child Health (DI-NOGMI), University of Genoa, 16132 Genoa, Italy; cristinapastorino22@gmail.com

**Keywords:** cystic fibrosis, F508del-CFTR, CFTR corrector, aminoarylthiazole, VX-809, docking

## Abstract

Cystic fibrosis (CF) is a genetic disease affecting the lungs and pancreas and causing progressive damage. CF is caused by mutations abolishing the function of CFTR, a protein whose role is chloride’s mobilization in the epithelial cells of various organs. Recently a therapy focused on small molecules has been chosen as a main approach to contrast CF, designing and synthesizing compounds acting as misfolding (correctors) or defective channel gating (potentiators). Multi-drug therapies have been tested with different combinations of the two series of compounds. Previously, we designed and characterized two series of correctors, namely, hybrids, which were conceived including the aminoarylthiazole (AAT) core, merged with the benzodioxole carboxamide moiety featured by VX-809. In this paper, we herein proceeded with molecular modeling studies guiding the design of a new third series of hybrids, featuring structural variations at the thiazole moiety and modifications on position 4. These derivatives were tested in different assays including a YFP functional assay on models F508del-CFTR CFBE41o-cells, alone and in combination with VX-445, and by using electrophysiological techniques on human primary bronchial epithelia to demonstrate their F508del-CFTR corrector ability. This study is aimed (i) at identifying three molecules (**9b, 9g,** and **9j**), useful as novel CFTR correctors with a good efficacy in rescuing the defect of F508del-CFTR; and (ii) at providing useful information to complete the structure–activity study within all the three series of hybrids as possible CFTR correctors, supporting the development of pharmacophore modelling studies, taking into account all the three series of hybrids. Finally, in silico evaluation of the hybrids pharmacokinetic (PK) properties contributed to highlight hybrid developability as drug-like correctors.

## 1. Introduction

Cystic fibrosis (CF) is an autosomal recessive disease caused by several mutations involving the cystic fibrosis transmembrane conductance regulator (CFTR), a gene encoding for a chloride/bicarbonate channel expressed on the apical side of epithelial cells of different organs, including lungs, pancreas, and testis [1,2]. CFTR is a multidomain transmembrane protein constituted by four domains including the Membrane-Spanning Domains MSD1 and MSD2, as well as the Nucleotide-Binding Domains NBD1 and NBD2, and a regulatory (R) region whose phosphorylation regulates channel activity. Each MSD consists of six segments crossing the phospholipid bilayer and then turning into a particular channel pore through which anions may flow [3]. So far, more than 2000 mutations have been reported, which give a variety of molecular defects, but most are very rare and not all manifest a clinical phenotype [4]. The classification of mutations into six different classes can be extended, for example taking in account the observation of combinatorial defects for some of them and their susceptibility to modulators [5]. The deletion of Phe508 (F508del) represents the most frequent CFTR mutation involved in this pathology (about 90% of CF patients show at least one copy) [6].

F508del causes two main defects: (i) incorrect folding of proteins with consequent degradation and reduction in channel numbers; and (ii) a gating defect with consequent low probability of opening and reduction in the function for the remaining channels, as well as plasma membrane instability [7,8].

The first nucleotide binding domain (NBD1) results in a reduced stability caused by the deletion of phenylalanine 508 (F508del) that alters the interactions between NBD1 and NBD2, as well as between NBD1 and the membrane spanning domains (MSD) [9].

To overcome these defects turning into CF, two kinds of pharmacological modulators are used, known as CFTR correctors, and able to increase the amount of F508del-CFTR protein properly folded to the plasma membrane, and CFTR potentiators to allow effective gating (i.e., function) of F508del-CFTR [10,11,12].

Potentiators appear to bind directly to mutant CFTR to foster channel gating following CFTR phosphorylation by a PKA protein kinase [10], while the action of the correctors is more disparate since some act as pharmacological chaperons, while others are essentially regulators of proteostasis [13,14].

Regardless of their mechanism of action, the administration of a single CFTR modulator does not seem to be sufficient in any case to obtain a therapeutically relevant rescue of F508del-CFTR [15,16].

In fact, using a corrector such as VX-809 (Lumacaftor) alone has little efficiency, whereas by combining it to a potentiator such as VX-770 (Ivacaftor), the function and the activity were improved [17].

However, there is only a modest clinical benefit [18] as uncorrected single defects remain and, at the same time, an increase in the turnover rate may occur following chronic treatment with VX-770 [19].

The most widespread opinion is that administering combinations of CFTR correctors with different mechanisms of action and featuring additive/synergistic efficacy can be effective [20,21,22].

In recent years, a new subdivision of correctors has been introduced (i) type I correctors, targeting the NBD1/MSD1 and NBD1/MSD2 interfaces; (ii) type II correctors, targeting NBD2 and/or its interfaces; and (iii) type III correctors, acting on the folding and stability of NBD1. The possible interaction between the correctors leads to consider that different binding sites can be exploited within the CFTR protein. Interaction with these sites can also lead to a better rescue of mutated proteins through allosteric phenomena [22].

The importance of binding multiple active sites on the protein to increase the correction is now also underlined by the improved clinical efficiency achieved by using Ivacaftor (VX-770), Tezacaftor (VX-661), and new discovered correctors such as VX-445 and VX-659, which seem to act on different sites of the total protein [23,24].

These encouraging results with combinations of three drugs stimulated the effort to discover new correctors with different mechanisms, because they have proven to be poorly effective in particular subgroups of patients. Hence, the need to look for new combinations capable of covering a wider range of patients and with greater efficacy than current combinations.

In the search of new CFTR modulators, during the last years, we designed and synthesized several aminoarylthiazoles (AATs) able to act as potentiators and/or correctors, featuring some of them synergistic and additive effect with VX-809 [25,26].

This allowed us to derive preliminary information that was exploited to create interesting correctors that were developed incorporating the AAT core, and merged with the benzodioxole carboxamide moiety that distinguished VX-809 (named hybrids) [27].

**Figure 1:**
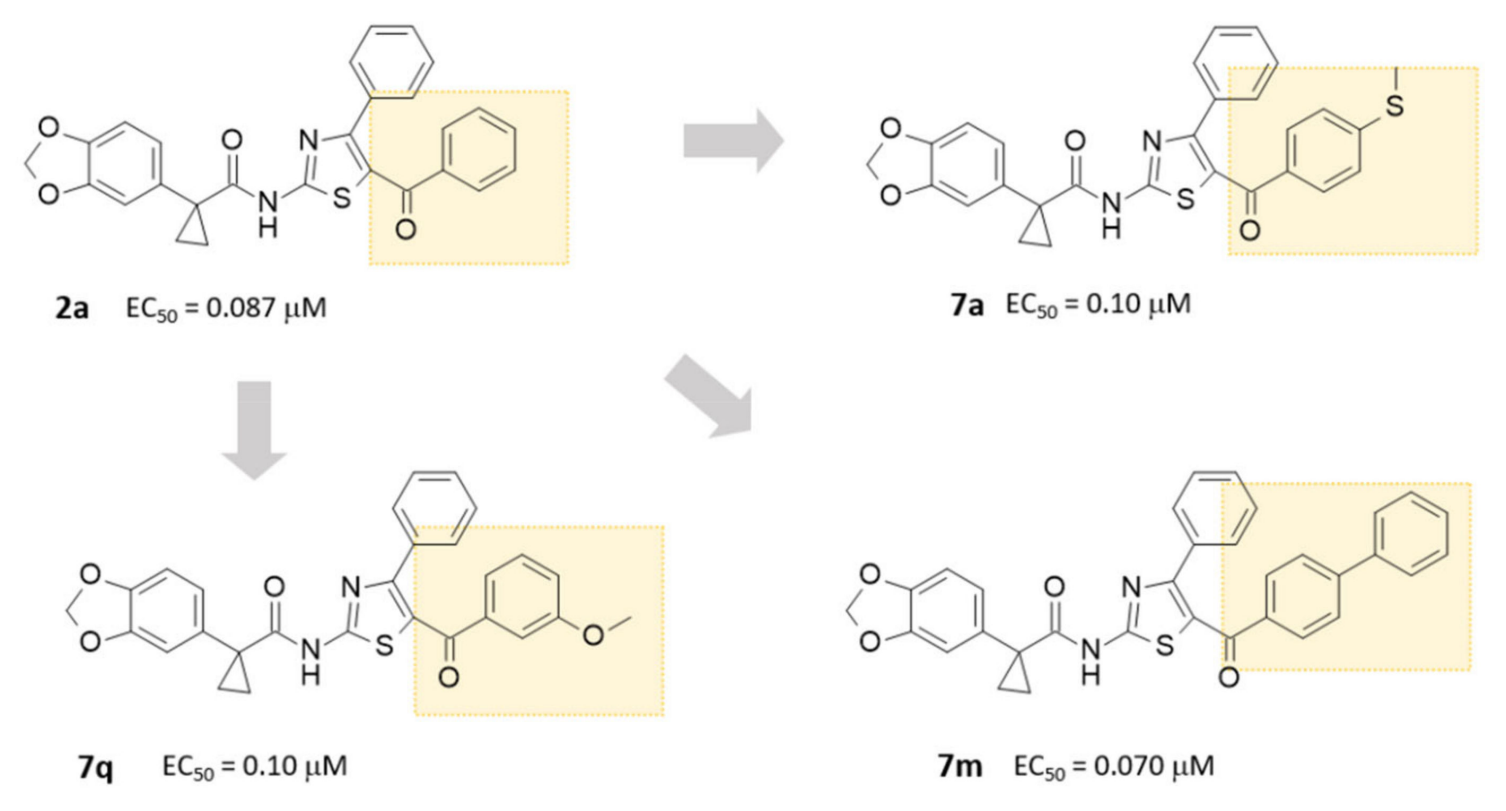
Chemical structure and biological activity of the previously developed hybrids as F508del CFTR correctors. Compound **2a** is represented as prototype of the first hybrids series while congeners **7** are reported as representative of the second one. Some of the most effective substitutions involving the thiazole position 5 are highlighted in light orange.

Among these, we designed a first series of hybrids, regarding CFTR rescue ability, with EC_50_ values between 0.09 and 0.2 µM, with derivative **2a** the most active (EC_50_ = 0.087 µM) (Figure 1).

In this frame, we improved our molecule’s library with a second series of optimized hybrids, showing improved potency values (EC_50_ values ranging from 0.020 to 0.10 µM), achieving these results by modification carried on portions belonging to the main scaffold of the molecule.

In this second round, we adopted a strategy leading to several promising compounds such as **7a, 7m,** and **7q**, with comparable or higher potency than **2a** (EC_50_ = 0.10 µM, EC_50_ = 0.070 µM (Figure 1) [28].

In this second series of substituted analogues, we investigated the substitutions of the phenyl ring in position 5, keeping unchanged, for the purpose of comparison, the thiazole core’s phenyl ring at position 4.

Inspired by these results, we herein proceeded with structure-based studies guiding the rational design and identification of a third new series of derivatives, relying on molecular docking studies involving the previous two series of compounds. Indeed, deepening molecular docking calculations have been performed on the previously developed benzoyl-containing **2a** as well as **2a** analogues (second hybrids series), with structural modifications at the benzoyl moiety. The derived information allowed us to better point out potential targeting modifications on position 4 of the thiazole ring, checking whether this replacement can improve the activity as F508del-CFTR correctors.

Biological assays confirmed the expected F508del-CFTR corrector ability, leading to the identification of three molecules (**9b**, **9g**, and **9j**), as novel promising correctors. Furthermore, accompanying pharmacophore modelling and in silico prediction of PK properties allowed to better explore the structure–activity relationship (SAR) within all the three series of hybrids, giving also a useful perspective of the hybrids drug-like profile.

## 2. Results

### 2.1. Structure-Based Studies Guiding the Rational Design of Novel Derivatives

During the last years, we described the rational design process and the chemical synthesis of different series of VX-809 congeners [27,28], thought to be hybrid derivatives based on the benzodioxole-containing carboxamide motif of the reference corrector and the thiazole ring previously experienced by the in-house CFTR modulators [26].

Most of them proved to be promising F508del-CFTR correctors, being endowed by rescue activity in primary bronchial epithelia. As a result, these data confirmed the effectiveness of the di-substituted thiazole core as bioisostere of the methyl pyridine ring exhibited by VX-809.

In particular, while our first series of compounds had been designed relying on QSAR methods, more recently we applied structure-based studies based on the NBD1 X-ray data of the F508del-CFTR protein to support the synthesis of the second series of hybrids [28]. Then, we focused on the development of a whole F508del-CFTR model to be exploited for further molecular docking calculations [29].

Briefly, we proceeded bearing in mind the data described in the literature concerning VX-809 as a full-length F508del-CFTR-targeting compound, with a putative binding site at the NBD1-ICL4 interface [16,30]. Indeed, recent approaches explored the docking mode of different F508del-CFTR correctors and VX-809 based on the whole CFTR protein, applying a combined surface plasmon resonance (SPR) and molecular dynamic (MD) simulation strategy, revealing key contacts supporting the modulator binding and the related corrector ability [31]. The main interactions, therefore, detected included the NBD1 residues S492, W496, and R560 as well as the ICl4 amino acids K1060 and W1063.

**Figure 2:**
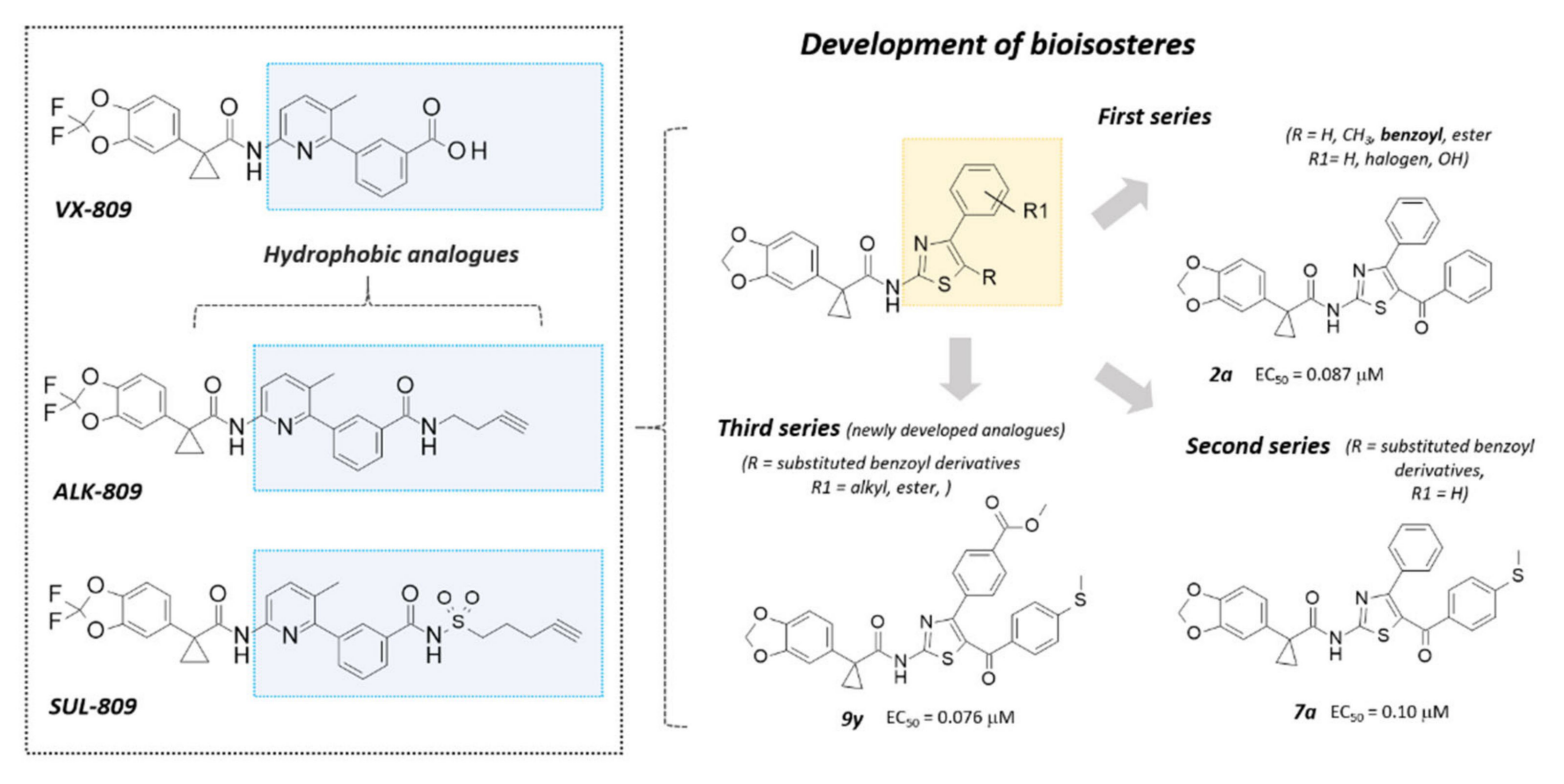
Chemical structure of VX-809 and of the related congeners ALK-809 and SUL-809. Structural variations featured by the in-house hybrids are also shown.

Accordingly, we described the results obtained by molecular docking of VX-809, ALK-9809, and SUL-809 at our modelled F508del-CFTR, which has been built thanks to the available data of the wild-type protein (pdb code: 6MSM) acquired by cryoelectron microscopy [29,32]. In particular, taking in consideration the structural variations experienced by the two correctors ALK-809 and SUL-809, bringing an amide and a sulfonamide function to replace VX-809 carboxylic moiety (Figure 2), allowed the role played by hydrophobic groups in F508del-CFTR targeting to be explored [29].

Based on our results, VX-809 proved to be highly stabilized within the protein crevice via salt-bridges involving the acid moiety and R552, K1292. The two oxygen atoms of the corrector benzodioxole ring were also H-bonded to K1060 [29].

Both the two analogues ALK-809 and SUL-809 showed one H-bond with K1351 thanks to the amide moiety and further H-bonds to L1062, W1063, and I1295 (see Figure S1). This kind of docking mode allowed orienting the hydrophobic terminal chains of the two correctors in the hydrophobic cavity surrounded by L1059, W1063, D1341, and C1344 (Figure S2).

Regarding our first series of hybrids (see chemical structures in Table S1), the most potent derivatives were decorated with a carbonyl group (such as **2a**) or an ester moiety (such as **2b** and **2c**) linked to the position 5 of the thiazole (see Figure 2) [27]. As previously reported, concerning the ester-containing derivatives, the benzodioxole group and the carboxamide moiety allowed the hybrid to be anchored at the ICL4 and NBD1 domain, featuring one H-bond with K1060 and S495, while the two substituents linked at the thiazole positions 4 and 5 especially interacted with ICL4 [29]. In detail, as shown for the **2b** corrector (Figure S2), the presence of hydrophobic groups at the thiazole position 5 guarantees van der Waals contacts and π–π stacking with the L1059 and W1063 residues, being the oxygen atom of the ester moiety H-bonded to W1063.

On the other hand, the presence of a proper pendant at the position 4 plays a key role being placed between the NBD1 and ICL4 portion of the channel. Indeed, the 4-Br-phenyl ring of **2b** was engaged in van der Waals contacts and π–π stacking with S492, T1064 and W496, W1063, respectively.

Conversely, the most potent compound **2a** lacking of flexible chains at the position 5 of the main ring, experienced a higher potency profile being able to detect more contacts and H-bonds with the protein, similarly to ALK-809 and SUL-809 [29].

As shown in Figure S3, **2a** maintained the key H-bonds with K1351 and I1295 by means of the carboxamide and benzodioxole motif as described for the previous reference correctors while the carbonyl moiety obliged the compounds to move only the unsubstituted phenyl ring linked at the thiazole position 4 towards S492, F494 and W496, W1063, detecting hydrophobic and π–π interactions. These data suggested a pivotal role determined by lipophilic substituents onto the main thiazole ring, in order to achieve lipophilic contacts with the channel, especially involving the aforementioned aromatic residues F494, W496, and W1063. On the other hand, H-bonds with K1351 and I1295 seem to guarantee the F508del-CFTR corrector activity.

Herein, deepening molecular docking calculations performed on the most promising derivatives discovered within the second series of hybrids (see Table S2) allowed the SAR of the compounds to be better explored. The estimated binding affinity values for the protein-corrector complexes are reported in Table S3.

**Figure 3:**
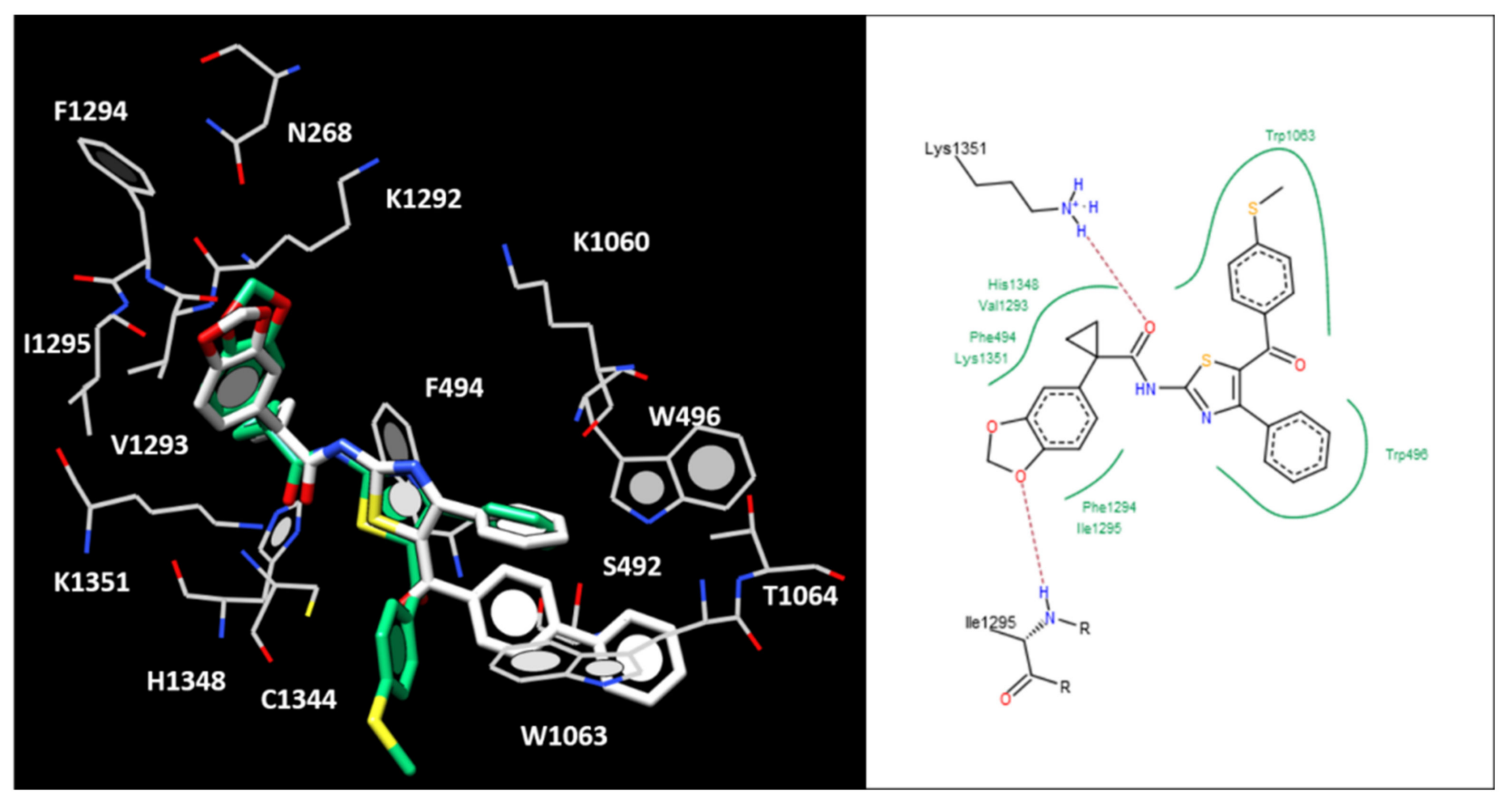
Docking positioning at the modelled F508del-CFTR of **7a** (C atom; light green) and **7m** (C atom; white), chosen as representative of the hybrids second series. The hybrid **7a** ligplot is shown on the right.

Thus, the introduction of the thiomethyl group or the biaryl motif at the benzoyl para position of the prototype **2a** led to the effective analogues **7a** (EC_50_ = 0.10 μM) and **7m** (EC_50_ = 0.07 μM), whose docking positioning underlined several contacts with the aromatic residues F494, W496, and W1063. Notably, the biaryl group of **7m** was properly projected towards F494, aW496, and W1063, displaying π–π stacking (see Figure S4), while the smaller thiomethyl moiety of **7a** was oriented in proximity of C1344 featuring hydrophobic interactions (Figure 3).

**Figure 4:**
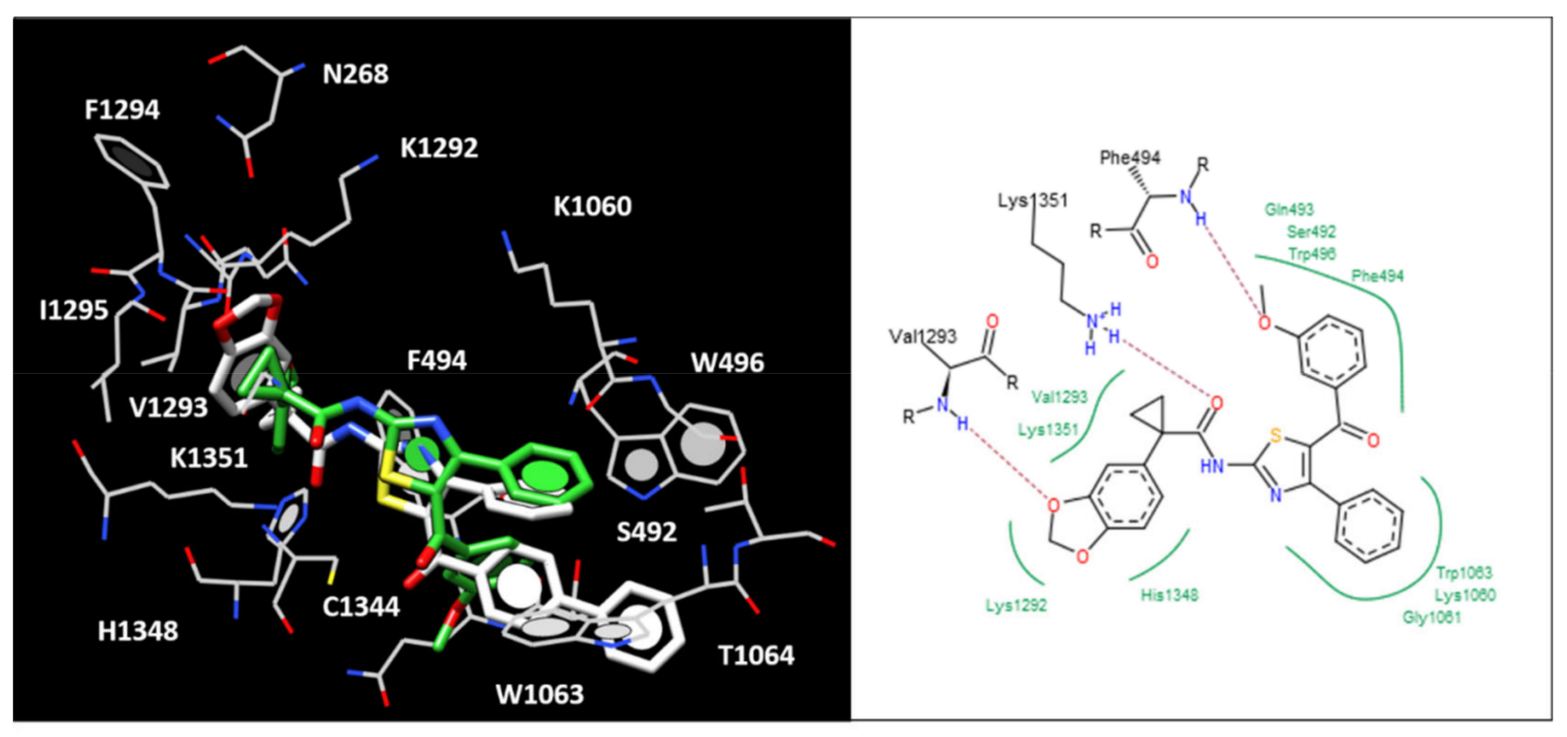
Docking positioning at the modelled F508del-CFTR of **7q** (C atom; light green) and **7m** (C atom; white). The hybrid **7q** ligplot is shown on the right.

Notably, both the two analogues placed the unsubstituted phenyl ring near W464, exhibiting further π–π stacking (Figure 4). This kind of positioning was quite maintained also by the corrector **7q** (EC_50_ = 0.10 μM), exhibiting a polar substituent onto the benzoyl ring. Indeed, **7q** experienced the aforementioned contacts between the phenyl ring and W496 and W1063, while the methoxy group was engaged in H-bonds with F494. As a consequence, the benzodioxole ring and the carboxamide function were H-bonded to V1293 and K1351, respectively.

On this basis, herein, we report the chemical synthesis and biological evaluation, followed by molecular docking studies, of a novel third series of hybrids. To address this issue, chemically different substitution involving the phenyl group linked at the position 4 of the main thiazole core had been taken into account. The applied chemical variations, involving a different position of the main thiazole core, allowed to optimize the SAR of hybrids providing the opportunity for the development of optimized analogues.

### 2.2. Chemistry

We applied a known synthetic way with suitable modifications to synthesize all the derivatives [27]. Cyclopropanation of active methylene compounds has been used as the way to obtain the benzodioxole substituted portion, as published [27]. A multi-strategy approach has been used to obtain aminoarylthixoles with different substitutions in position 4 of the thiazole ring. For the structures of the different (2-amino-4-arylthiazol-5-yl)(aryl)methanone derivatives, a convergent synthesis, according to the Wang protocol, has been developed with modifications [33].

**Scheme 1:**
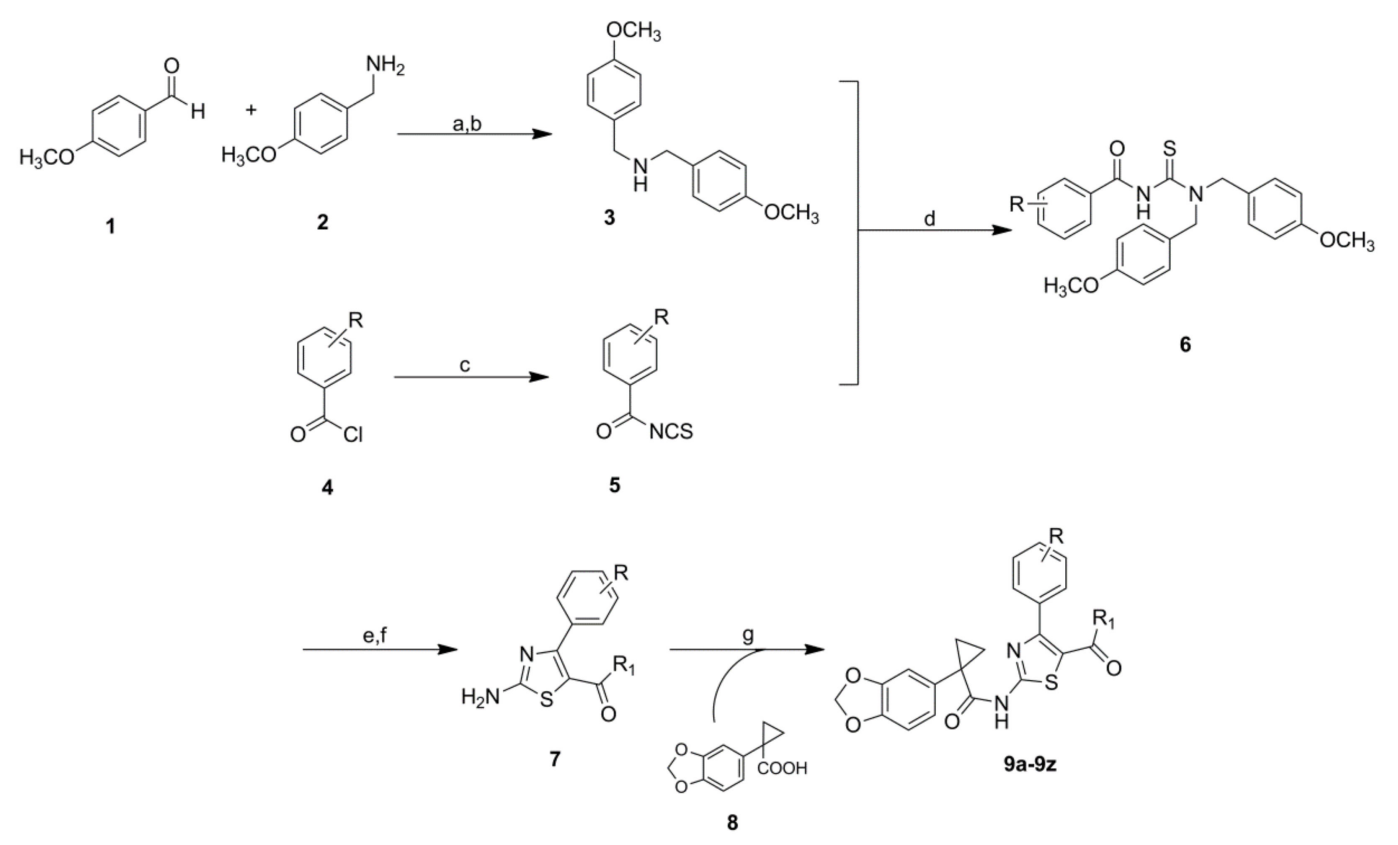
Reagents and conditions: (**a**) methanol, reflux, 3 h; (**b**) NaBH_4_, 0 °C to RT, 10 h; (**c**) NH_4_SCN, acetone, 0 °C, 2 h, 80–85%; (**d)** acetone, RT, 2–3 h, 53–91%; (**e**) R_1_COCH_2_Br, N,N-DMF, 85 °C, 3 h; (**f**) TFA, 100 °C, 36–48 h, 41–73%; (**g**) HATU, DIPEA, N,N-DMF, 50 °C, 24–48 h, 17–43%.

The synthetic route to the compounds **9a–9z** is reported in Scheme 1.

To have a suitable introduction of the various groups on the thiazole ring, a protected carbamothioyl amide was conjugated with α-bromoketone substituted. The reaction of 1-[4-(methoxy)phenyl]methanamine **1** and 4-(methoxy)benzaldehyde **2** in methanol at reflux, followed by the reduction with NaBH_4_, results in the formation of bis(4-methoxybenzyl)amine **3**.

Condensation of acyl chloride **4** and thiocyanate with bis(4-methoxybenzyl)amine **3** allows a protected carbamothioyl amide **6** in high yield (>50%) to be obtained.

The thiazolic derivatives **7** were provided by condensation of α-bromoketone with protected carbamothioyl amide, followed by deprotection; then, aminothiazoles were conjugated with 1-(benzo[d][1,3]dioxol-5-yl)cyclopropanecarboxylic acid **8** to produce the expected compounds **9a–9z.**

This final step was obtained by reaction of 2-amino-thiazole with the carboxylic portion of **8** with uronium salt activation in anhydrous DMF [27].

**Scheme 2:**
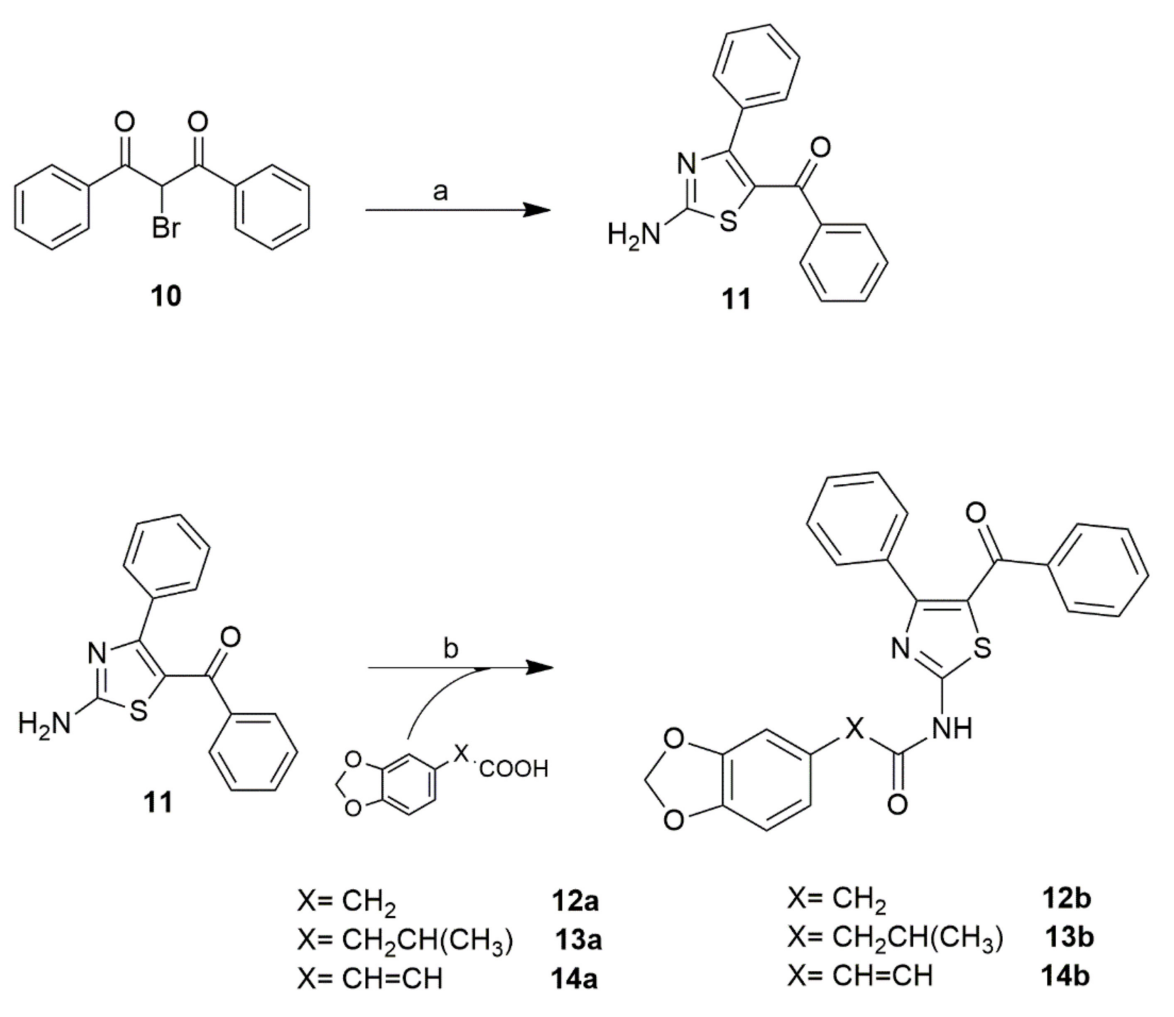
Reagents and conditions: (**a**) thiourea, ethanol, reflux, 2 h, 75%; (**b**) HATU, DIPEA, N,N-DMF, 50 °C, 18–24 h, 40–45%.

For the compounds **12b**,**13b**, and **14b** a traditional Hantszch protocol was used to furnish (2-amino-4-phenylthiazol-5-yl)(phenyl)methanone **11.** In particular, **11** was achieved in high yield by the condensation of 2-bromo-1-,3-diphenylpropane-1,3-dione **10** with thiourea in refluxing ethanol. The synthesis of amide derivatives of 2-aminothiazoles (**12b–14b**) was achieved by reaction of **11** with the suitable carboxylic derivatives (**12a–14a**) with HATU/ DIPEA activation in anhydrous DMF (Scheme 2).

**Scheme 3:**
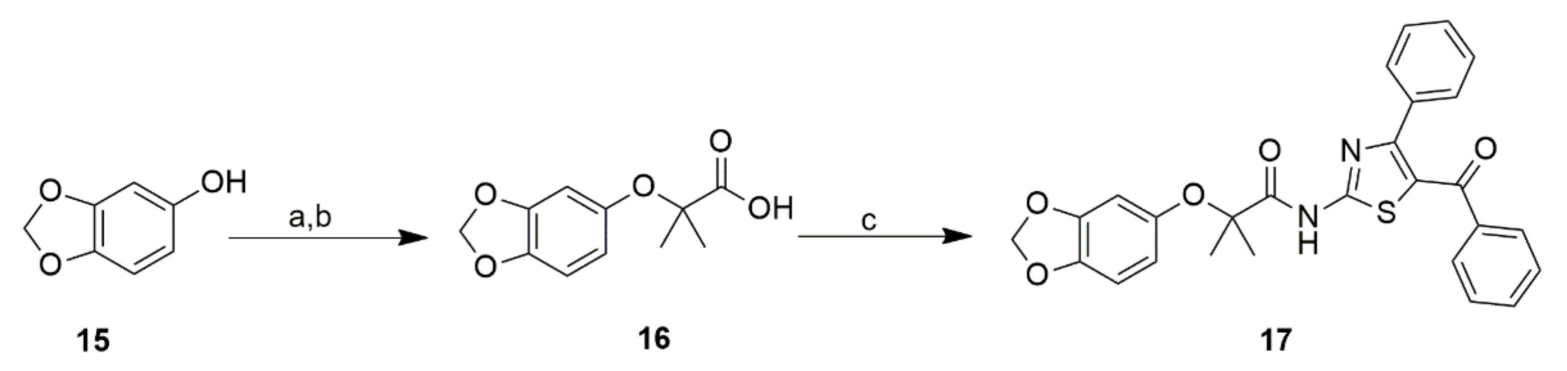
Reagents and conditions: (**a**) ethyl-2-bromoisobutyrate, K_2_CO_3_, N,N-DMF, RT, 16 h; (**b**) KOH 6N, ACN, 60 °C, 3 h, 61%; (**c**) 11, HATU, DIPEA, N,N-DMF, 50 °C, 24 h, 22%.

In Scheme 3, ethyl 2-(benzo[d][1,3]dioxol-5-yloxy)-2-methylpropanoate was prepared by reaction of ethyl-2-bromoisobutyrate and benzo[d][1,3]dioxol-5-ol **15** in DMF at room temperature in the presence of K_2_CO_3_. Hydrolysis of substituted ester under aqueous basic conditions yielded acid derivative **16**. Amide coupling of **16** with 2-amino-4-phenylthiazol-5-yl)(phenyl)methanone afforded final compound **17** with a yield of 22%.

For the synthesis of compound **18** the aminothiazole **11** was conjugated with 1-(4-chlorophenyl) cyclopropanecarboxylic acid with HATU/DIPEA activation in anhydrous DMF to afford the derivative in good yield.

### 2.3. Structure-Activity Relationship of Hybrids Third Series

Based on one our previous study [27], the introduction of a benzoyl moiety at the main thiazole position 5 led to one of the most effective hybrids so far developed, compound **2a** (EC_50_ = 0.087 μM) (Figure 1). In our recent data, a number of congeners involving possible structural variations at the prototype benzoyl group were explored, herein we proceeded with further substitutions, especially at the thiazole position 4.

**Figure 5:**
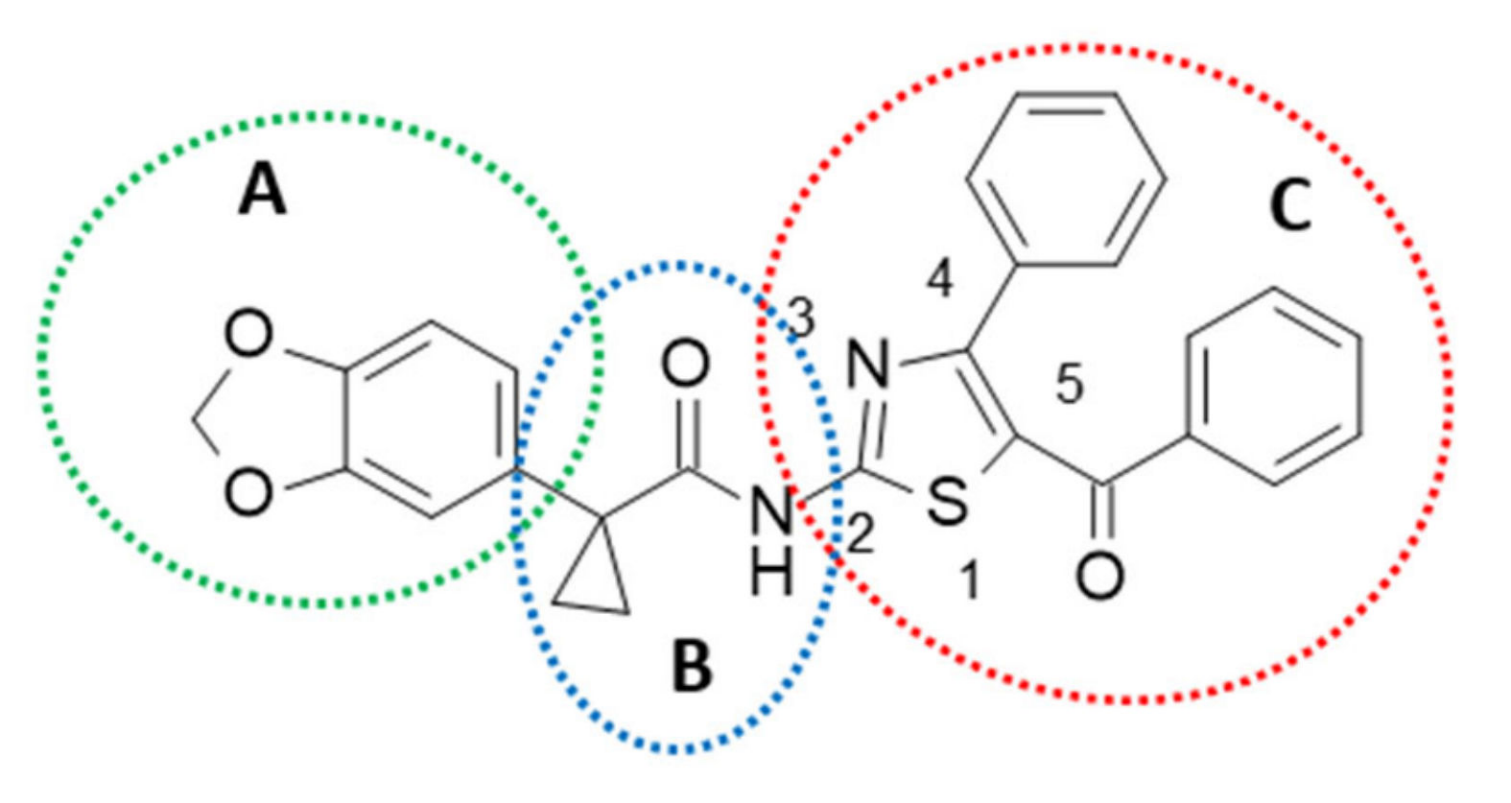
Sites of chemical modifications on the structure **2a** to explore the SAR of the thiazole hybrids.

First, based on the aforementioned information regarding structure-based studies, we were interested in better exploring the role played by the different portions of the hybrid turning into improved biological activity. Notably, compound **2a** can be divided into three different fragments of similar size **A,B** and **C** (Figure 5).

The SAR study was based on the study of the role of the benzo[d][1,3]dioxole portion (**A**), the substitution of cyclopropane carboxamide moiety (**B**), and the modification of the pattern substitution on the thiazole ring (**C**).

Then, to find selective and potent correctors of F508del-CFTR trafficking defect, all derivatives were then tested to evaluate the structure–activity relationships as F508del-CFTR correctors (Table 1 and Table 2).

The ability of these compounds to correct the basic defects related to F508del mutant was evaluated on immortalized CFBE41o-bronchial epithelial cells stably expressing F508del-CFTR and the halide sensitive yellow fluorescent protein (HS-YFP).

By using the HS-YFP functional assay, we tested all compounds at different concentrations and after 24 h of incubation to derive the EC_50_ values of the derivatives as promising correctors of the explored mutant CFTR (Table 1 and Table 2).

The measurements of the rate of HS-YFP quenching caused by iodide influx allowed us to determine the activity of F508del-CFTR, after incubation with the compounds, as cited [27]. Activity was then compared to that of cells treated with the corrector VX-809 (1 µM) or with vehicle alone (DMSO).

Initially, the role of the benzo[d][1,3]dioxole portion **(A**) was explored by modifying the substituent on the phenyl ring while keeping the cyclopropanecarboxamide moiety (B) unmodified (Table 1).

The results demonstrated our modification in which 1-(benzo[d][1,3]dioxol-5-yl)cyclopropanecarboxamide of **2a** was replaced by 1-(4-chlorophenyl)cyclopropanecarboxamide as poorly effective, leading to the derivative **18** (Table 1). These data were in accordance with a previous paper about different correctors containing the 4-methoxyphenyl cyclopropane carboxamide moiety, which were inactive [34], and proved the importance of benzodioxole moiety (**A**) for the F508del-CFTR correction activity.

We next investigated the utility of cyclopropane carboxamide portion (B) in the entire structure. To demonstrate this concept, we keep intact 2-amino-(4-phenylthiazol-5-yl)phenylmethanone, aminothiazole core of **2a**, and the cyclopropane carboxamide was replaced by different chemical scaffolds.

First, we synthesized the parent molecule without a cyclopropane ring (**12b**), but this derivative was inactive. After this, we built two different compounds in which we inserted, respectively, isobutyramide (**13b**) and acrylamide portions (**14b**). This modification should have led to a less rigid scaffold, possibly resulting in increased affinity at the target binding site, but also these substitutions are not tolerated (Table 1).

Finally, the cyclopropane carboxamide was replaced by aryloxy-2-methylpropanamide moiety. This modification should create a rigidity comparable to cyclopropane moiety and at the same time a new point of target binding through the oxygen atom of the chain.

To explore the effect of this substituent, 2-(benzo[d][1,3]dioxol-5-yloxy)-2-methylpropanoic acid was conjugated with the 2-amino-4-phenylthiazol-5-yl)(phenyl)methanone to obtain derivative **17**. Unfortunately, this substitution results in lowered efficacy, proving that this replacement is not suitable for amide containing correctors (Table 1).

Several trends were observed regarding the importance of the benzo[d][1,3]dioxole cyclopropane carboxamide moiety (**A** + **B**) for corrector activity of this class as shown in Table 1.

Nevertheless, among F508del-CFTR correctors, the above cited structure is an essential feature across otherwise diverse chemical scaffolds, suggesting that this portion is a key pharmacophore for corrector efficacy for our and other derivatives [11,15,34,35,36].

As a further step in the study of SAR within this chemotype, different thiazole molecules were also explored. On this basis, we designed a small series of thiazole compounds (Table 2), inspired by a number of congeners previously developed within the first and second series of hybrids [27,28]. We proceed taking into account some of the discovered effective substituents at the thiazole position 5 and then applying further variations at the position 4. This allowed us to progressively decorate the hybrid scaffold and to obtain some information on activity as possible correctors.

In fact, we already showed that the substitutions on positions 4 and 5 of the thiazole ring in portion **C** were necessary for the activity since the most interesting molecules of the previous libraries all held substituents in these positions [27,28].

In particular, a hindered substituent, such as benzoyl or ethyl acetate, at the position 5, could be useful to improve some pharmacokinetic characteristics, while with the analogues that did not have any substitution, a decrease in the corrector activity was observed [27].

So, we decided to synthetize 4,5 disubstituted thiazole derivatives as represented in Figure 5 and Table 2. Whereas in our previous study [28], the developed second series of hybrids maintaining an unsubstituted phenyl ring was tethered to the position 4 of the thiazole; herein, we placed our attention on substitutions involving the phenyl ring linked to the same position of the thiazole.

First, we investigated different phenyl-substituted analogues starting from a para position.

We inserted a methoxy group as in **9a** but a decrease in activity was observed (EC_50_ = 0.35 µM) respect to the **2a** (EC_50_ = 0.087 µM). So, we decided to modify the substituent in position 5, inserting different substituted phenyl derivatives fixing the methoxy group in a para position (see Table 2). With the presence of p-methoxy portion like in **9b,** an increase in potency was obtained (EC_50_ = 0.064 µM). A similar trend was observed with the presence of trifluoromethoxy group **9c** (EC_50_ = 0.14 µM) in the same position too.

In the same position, different electron-withdrawing groups such as methylthio group **9d** (EC_50_ = 0.10 µM) or ethyl ester derivative **9e** (EC_50_ = 0.56 µM) were inserted, but only in the case of the methylthio group was the potency conserved.

Then, two different compounds were designed with a second aromatic phenyl ring **9f** (EC_50_ = 0.14 µM) or hetero aliphatic ring **9g** (EC_50_ = 0.033 µM). As reported in Table 2, compound **9g** showed an increase in activity and an EC_50_ lower than the reference compound **2a** (EC_50_ =0.087 µM).

On the contrary, by shifting the methoxy group in 5 from the para to the meta position like in **9h,** a small decrease in activity (EC_50_ = 0.12 µM) was evaluated.

After examining the influence of the methoxy group in para, we moved the same substituent from the para to the meta position of the phenyl ring in 4.

Almost all the same para-substituents were considered as for **9i** EC_50_ = 0.19 µM, **9j** EC_50_ = 0.037 µM, **9k** EC_50_ = 0.36 µM, **9l** EC_50_ = 0.49 µM, **9m** EC50 = 0.74 µM, and **9n** EC_50_ = 0.6 µM), but only the p-methoxy **9i** (EC_50_ =0.19 µM) and, overall, the trifluoromethoxy group **9j** maintained a high activity (EC_50_ = 0.037 µM).

Indeed, we built a derivative **9o** containing two methoxy groups in a meta position in the phenyl ring at both positions 4 and 5. The compound bearing methoxy group in the meta position of 5 position was slightly less active respect to that with para substitution (compare **9o** EC_50_ = 0.21 µM with **9i** EC_50_= 0.19 µM) indicating that the para position is preferential for the substitution on phenyl ring in 5.

Then, the importance of the methoxy group on phenyl ring was broadened by synthetizing analogues where the methoxy residue was in ortho. Based on the promising effects shown by **9b**, **9i** (p-OCH3) and **9c, 9j** (p-OCF3) in dose-response data in F508del-CFTR FRT cells, we maintained the same substituents in 5, and two analogues with the methoxy group in ortho were synthesized to achieve additional data on the SAR of this class (Table 2).

The two compounds **9p** (EC_50_ = 0.22 µM) and **9q** (EC_50_ = 0.15 µM) were always active but the ortho position decreased the activity with respect to the meta and para positions. Compare, for example, **9b** (EC_50_ = 0.064 µM) with **9i** (EC_50_ = 0.19 µM) and **9p** (EC_50_ = 0.22 µM).

In our previous data [28], we evaluated how the most active compounds of the series retained phenyl or thiomethyl in the para position or the methoxy group in meta in position 5 (see compounds **7a**, **7m,** and **7q** in Figure 1), keeping an unsubstituted phenyl ring in position 4. In an attempt to improve their potency, we modified the phenyl rings at position 4 with different chemical groups, keeping intact the same moiety in position 5.

First, we synthetized and evaluated compounds with different residues in the para position of the phenyl ring in 4 (methyl ester, methylthio, pyridine, and chlorine group) keeping unchanged the biphenyl portion in 5. A similar positive effect was displayed in **9r** (EC_50_ = 0.12 µM), **9s** (EC_50_ = 0.13 µM), and **9t** (EC_50_ = 0.13 µM), while an opposite behavior was detected when the phenyl ring in 4 was replaced with a p-chlorine as in **9u** (EC_50_ = 0.64 µM).

On the contrary, with the presence of m-OCH3 in position 5, only weak corrector activity was maintained with p-SCH3 **9v** (EC_50_ = 0.28µM), decreasing both with a pyridinic ring **9w** (EC_50_ = 1.52 µM) and with chloride **9x** (EC_50_ = 0.37 µM) (Table 2).

Then, we deepened the analogues with the p-SCH3 in position 5 with compounds such as **9y**, which contained methyl carboxylate in position 4, where the potency was conserved (EC_50_ = 0.076 µM), and **9z,** while the presence of another p-methylthio in the phenyl ring in 4 caused a marked loss of activity (EC_50_ = 0.3 µM).

Interestingly, comparing the results described, it is possible to obtain some hints underlining the structural requirements to achieve corrector ability.

Changing the compound **2a** with a replacement only on the phenyl ring in position 4 leaving at the same time unsubstituted phenyl group in position 5 does not improve the activity (compare **9a** vs. **2a**).

For the substitution in 4, the introduction of a methoxy group at the para position of the phenyl group is better than the meta and the ortho.

Well-tolerated in the para position are methylthio (**9s, 9v, 9z**) and methyl ester groups (**9r** and **9y**) while the introduction of a halogen (chloride in particular) in the same position is detrimental (**9u** and **9x)**. When the phenyl ring in 4 is replaced with a pyridinic ring the activity is maintained only with a biphenyl at position 5 (**9t**).

For the substitution in 5, the best group seems the p-OCH3 both with para and meta substitution into phenyl in 4 (see **9b** and **9i**), while p-OCF3, p-SCH3, pyrrolidine, and biphenyl are well tolerated if the phenyl group in 4 is only para substituted (**9c, 9d, 9f, 9g, 9r, 9s, 9t, 9y,** and **9z**). Interestingly all the derivatives having p-OCH3 are more active than the respective derivatives containing the isostere p-SCH3, underlining a better behavior for this substitution pattern on the phenyl ring in position 5 (compare **9b** with **9d** and **9i** with **9k**).

On the contrary, all the compounds bearing the same four substituents are less active if the m-methoxy moiety (**9k, 9m, 9n**) is present in 4, except for the trifluoromethoxy group (**9j**), one of the best compounds of the series (see Table 2).

Lastly, the simultaneous presence of two symmetric groups, one on the 4 position and the other on the 5 one of the phenyl rings, leads us some evaluations.

In detail, the compounds **9b** and **9h** containing methoxy groups in para on phenyl in 4 are active if both the same substituent is in para as in meta in 5. On the contrary, the activity decreases with compounds in which the methoxy group is in meta on the 4 position, regardless of the position of the substituent in 5 (**9i** and **9o**).

In addition, if the two substituents are methylthio groups as in the molecule **9z,** the corrector activity decreases in agreement with the idea that the p-OCH3 is more active than the respective derivatives containing p-SCH3.

In conclusion, we compared different hybrids with different functional groups on phenyl in position 4 of the thiazole ring (**9a–9z**) with the analogues (**7a–7z**) published in the previous paper [28].

The most active compounds of this series are: **9b,** which displays p-methoxy phenyl both at position 4 and at position 5; **9g** with a p-methoxy phenyl in 4 and a p-pyrrolidine phenyl in 5; and **9j** with a m-methoxy phenyl in 4 and a p-trifluoromethoxy phenyl in 5. Comparing their values of EC_50_ with the activity of analogues bearing the same groups at 5 and the phenyl with no substituents in 4, we can observe a significative improvement in the activity for our new hybrids (**9b** EC_50_ = 0.064 µM vs. **7h** EC_50_ = 0.45 µM, **9g** EC_50_ = 0.033 µM vs. **7n** EC_50_ = 0.37 µM and **9j** EC_50_ = 0.037 µM vs. **7i** EC_50_ = 0.36 µM).

### 2.4. Biological Assays of Novel Hybrids

**Figure 6:**
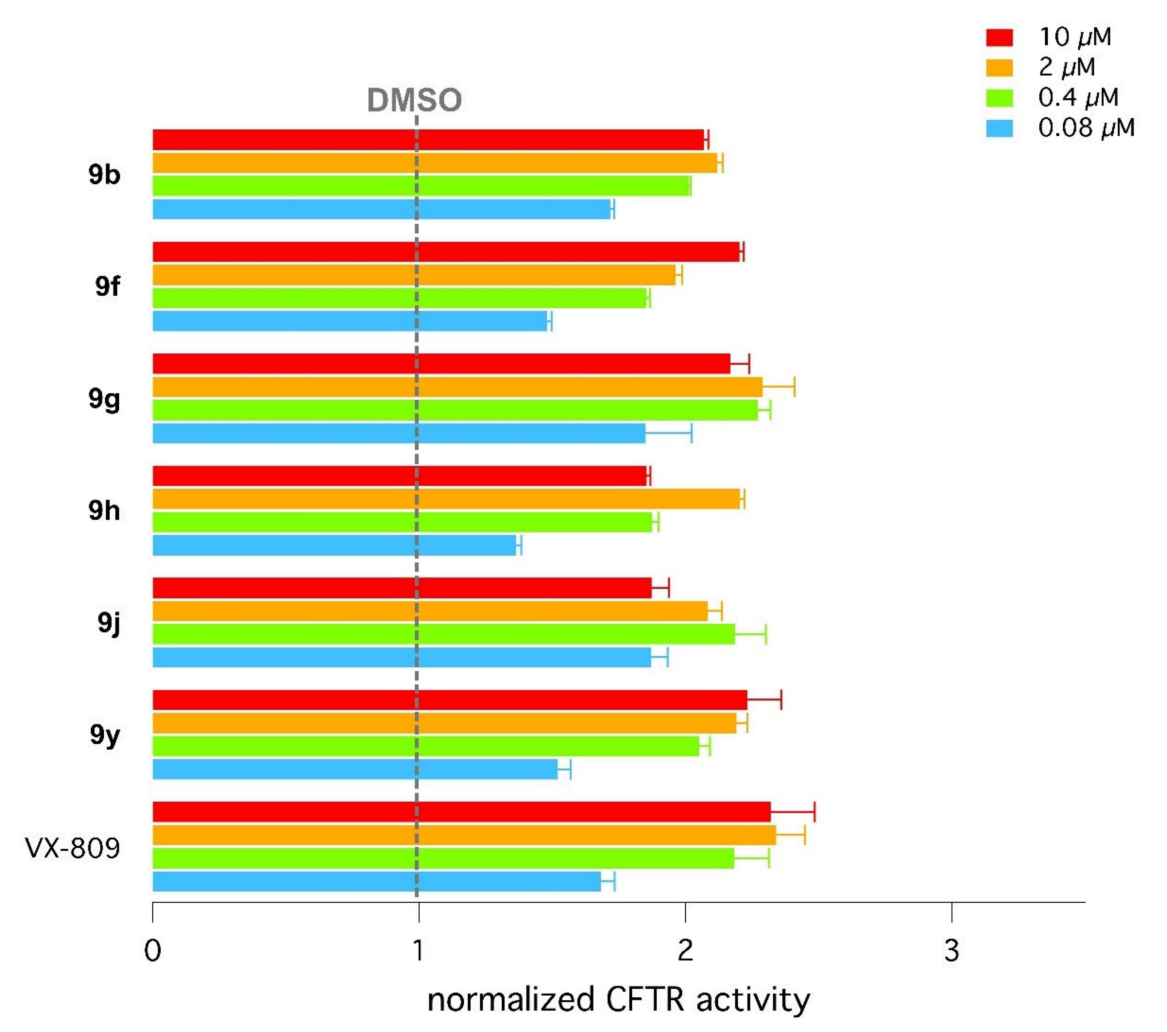
Efficacy of six active compounds on mutant CFTR rescue. The bar graph reports normalized F508del-CFTR activity, measured with the HS-YFP assay in F508del-CFTR-expressing CFBE41o-cells treated for 24 h with the indicated compounds at different concentrations. The dashed line indicates activity in cells treated with vehicle alone.

Within the novel third series of hybrids herein proposed, based on their potency and efficacy in terms of CFTR rescue, we selected six compounds and determined the dose–response relationships (Figure 6). To this aim, F508del-CFTR expressing CFBE41o-cells were treated for 24 h with our hybrids **9b**, **9f**, **9g**, **9h**, **9j**, and **9y** and then mutant CFTR activity was measured by using the YFP functional assay (see Materials and Methods section for details).

**Figure 7:**
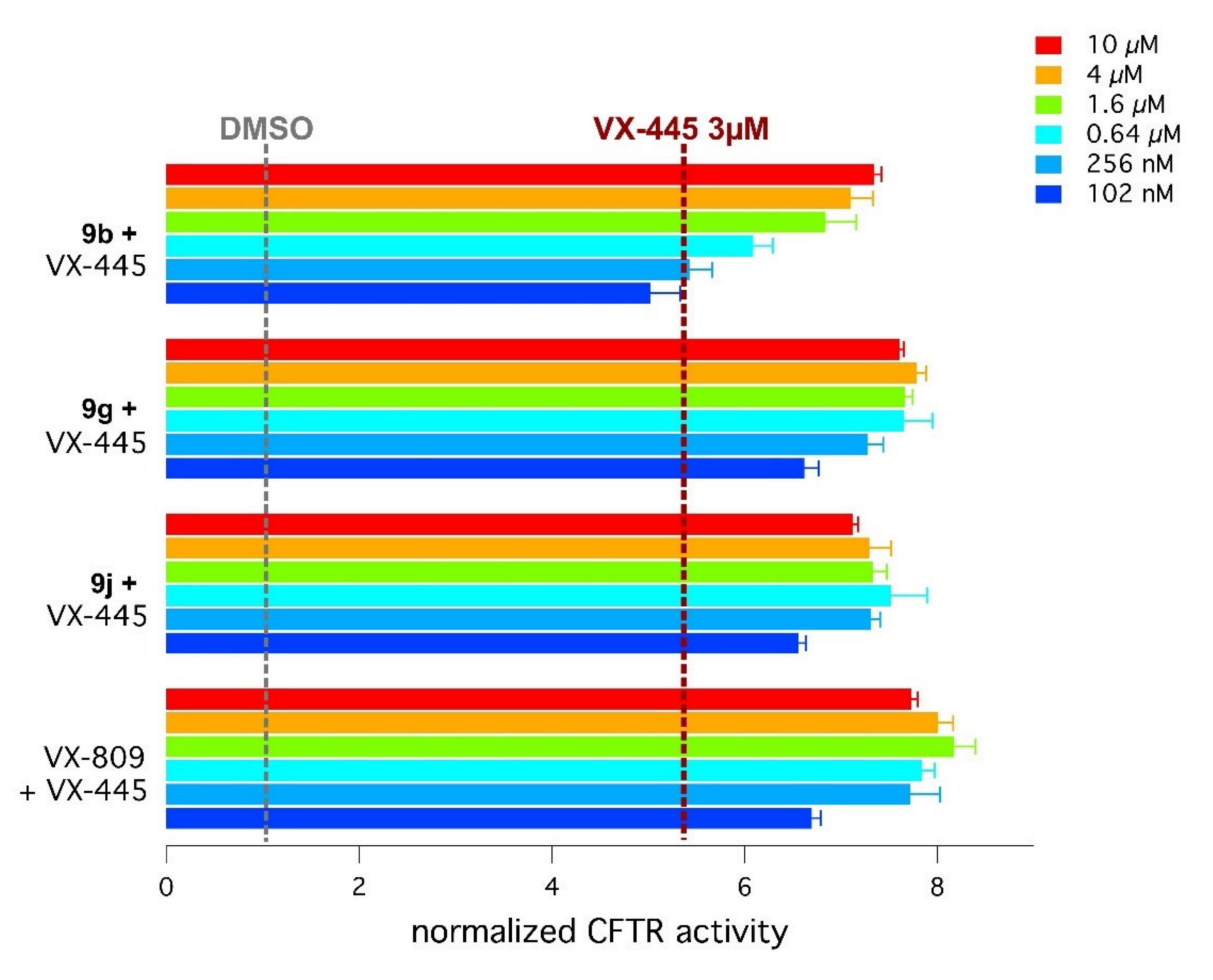
Functional analysis of corrector combinations. F508del-CFTR activity was determined in CFBE41o-cells with stable expression of the HS-YFP. Cells were treated for 24 h with VX-445 (3 µM) alone or in combination with **9b**, or **9g**, or **9j**, or VX-809 at different concentrations (from 102 nM to 10 µM). The YFP-based assay was performed after stimulation of CFTR activity with forskolin (20 µM) plus VX-770 (1 µM).

We have previously shown that the combination of our hybrids with VX-809 or VX-661 showed no additive or synergistic effect, sustaining the concept that all these hybrids may have the same binding site [28]. Now, to further characterize the activity of these compounds, we tested the novel derivates **9b**, **9g,** and **9j**, along with VX-809 as a positive control, in combination with VX-445 (3 µM), in CFBE41o-cells stably expressing F508del-CFTR by using the YFP functional assay (Figure 7). To this aim, cells were treated for 24 h with test compounds at different concentrations, in combination with VX-445 (3 µM), and then assayed after maximal stimulation of F508del-CFTR in the presence of forskolin (20 µM) plus VX-770 (1 µM). The three novel derivatives as well as VX-809 showed a clear additive effect with VX-445, in particular the combinations based on **9g** and **9j** were as effective as the combination with the positive control VX-809 in terms of maximal rescue and activity at low concentration.

**Figure 8:**
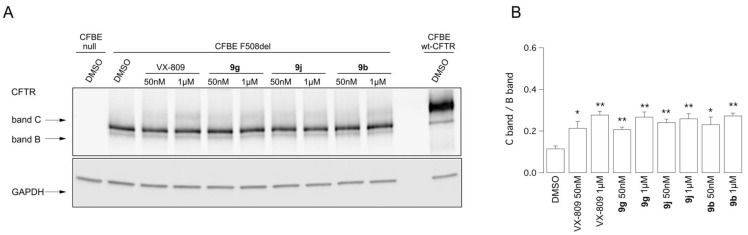
Biochemical analysis of the F508del-CFTR expression pattern: (**A**). The Western blot image shows the electrophoretic mobility of F508del-CFTR in CFBE41o- cells, treated for 24 h with vehicle alone or test compounds (as indicated). Arrows indicate mature, complex-glycosylated (band C) and immature, core-glycosylated (band B) forms of CFTR protein. (**B**). Quantification of the C band/B band ratio in experiments as in (**A**). Data are shown as mean ± SD (*n* = 3 independent experiments). Asterisks indicate statistical significance versus negative control: * *p* < 0.05; ** *p* < 0.01.

We then biochemically estimated the rescue of the trafficking defect by analyzing the electrophoretic mobility of mutant CFTR protein. In Western blots, CFTR protein can be detected as two bands B and C, of approximately 150 and 170 kDa, respectively. Band B corresponds to immature, partially glycosylated CFTR, while band C corresponds to the mature fully glycosylated CFTR. In cells expressing wild-type CFTR protein, the prevalent form is band C, while the main form in cells expressing F508del-CFTR is band B, in agreement with the severe trafficking defect caused by this mutation (Figure 8). To estimate the effect of our molecules on CFTR expression pattern, we treated F508del-CFTR/HS-YFP expressing CFBE41o-cells for 24 h with test compounds **9b**, **9g,** and **9j** (50 nM and 1 µM) VX-809 as a positive control or DMSO as a vehicle alone.

The next day, cells were lysed and the obtained lysates were subjected to SDS-PAGE followed by Western blotting (Figure 8A). Western blot images were analyzed with ImageJ software. CFTR bands, analyzed as ROI, were quantified in each lane after normalization for GAPDH as a loading control. Treatment of F508del-CFTR cells with **9b, 9g,** and **9j** resulted in a significant enhancement in the C band/B band ratio, similar to that with VX-809 (Figure 8B).

**Figure 9:**
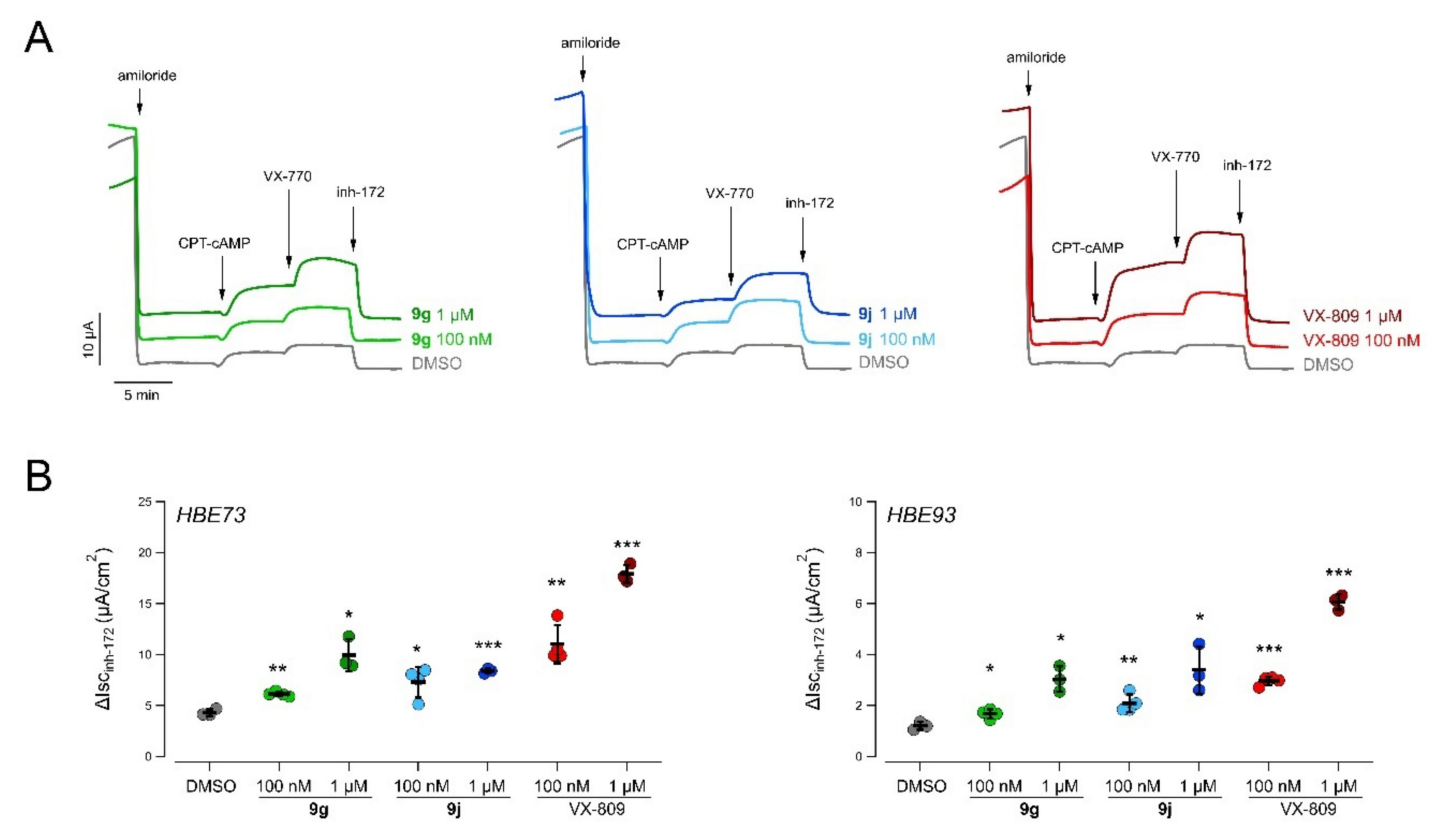
Evaluation of the effect of hybrid derivatives **9g** and **9j** on F508del-CFTR activity measured on human primary bronchial epithelia derived from two CF patients: (**A**). Representative traces from Ussing chamber recordings of human primary bronchial epithelia derived from a homozygous F508del patient (donor code: HBE73) following 24 h treatment with DMSO alone or indicated test compounds. (**B**). Dot plots summarizing CFTR-mediated currents from Ussing chamber recordings of human primary bronchial epithelia derived from two homozygous F508del patients (donor codes: HBE73 and HBE93) treated as described in (**A**). Analysis of variance was calculated for the groups. Asterisks indicate statistical significance versus negative control: * *p* < 0.05; ** *p* < 0.01; *** *p* < 0.001.

Then, we tested the ability of these compounds to rescue F508del-CFTR on well-differentiated primary cultures of human bronchial epithelial cells from two different F508del homozygous subjects (donor codes: HBE73 and HBE93) by using electrophysiological techniques. To this aim, bronchial cells were seeded on permeable supports and cultured until cells polarized and differentiated under air–liquid interface condition for 20 days. Bronchial epithelia were then treated for 24 h with DMSO vehicle alone (negative control) or with **9g** and **9j** (50 nM and 1 µM) two of the most important hybrids described in this paper. As positive control, we used VX-809 (treated at the same concentrations used for test compounds). The following day, epithelia were mounted in Ussing chambers for the measurement of chloride transepithelial transport by short-circuit current measurements (Figure 9). After blocking epithelial sodium channel (ENaC) activity with amiloride, cells treated with vehicle (DMSO) showed little CFTR function, as indicated by the small response to the membrane-permeable cAMP analog CPT-cAMP and the potentiator VX-770. The low activity of F508del-CFTR in the apical membrane was confirmed by adding the selective CFTR inhibitor, CFTRinh-172, which caused a relatively small current drop. As expected, 24 h incubation with corrector VX-809 resulted in dose-dependent significant F508del-CFTR rescue, as evidenced by the marked current increase elicited by stimulation with CPT-cAMP and VX-770 and the amplitude of the current drop caused by CFTRinh-172. Epithelia treated with **9g** and **9j** showed significant increase in CFTR-dependent function as compared to DMSO-treated epithelia; although, the rescue observed was lower than that achieved following treatment with VX-809 (Figure 9).

### 2.5. Molecular Docking of the Most Potent New Hybrids

The following molecular docking calculations allowed us to clarify some hints concerning the main SAR requirements featured by the new third series of hybrids as F508del-CFTR correctors. The estimated binding affinity values for the protein-corrector complexes are reported in Table S3.

Bearing in mind the previously discussed docking poses for the main precursors described as **7** compounds [28], the m-methoxy substituent or the p-SCH_3_ and the biaryl motif at the **2a** benzoyl group led to the promising **7q** (EC_50_ = 0.10 µM), **7a** (EC_50_ = 0.10 µM), and **7m** (EC_50_ = 0.07 µM), respectively.

**Figure 10:**
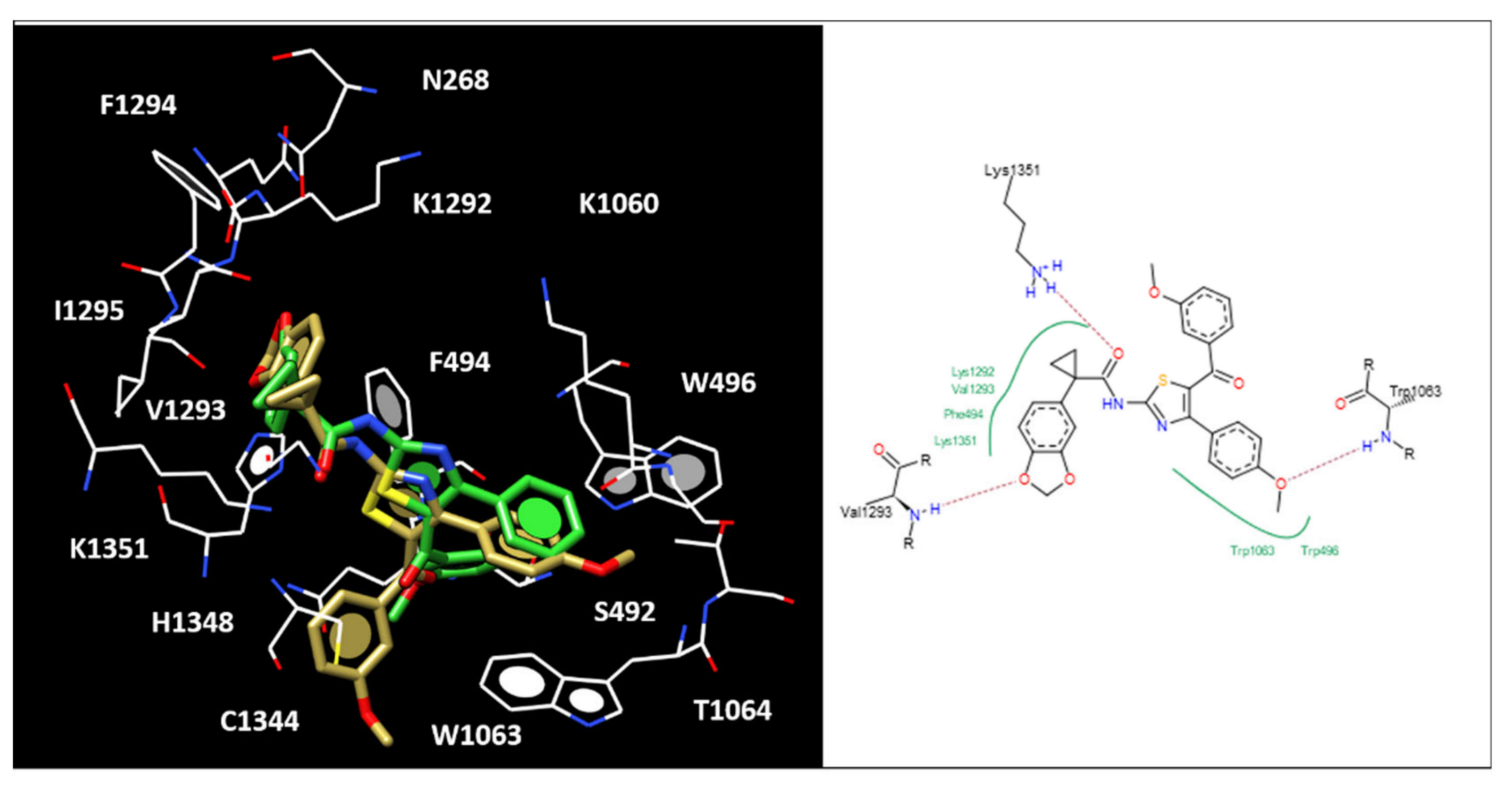
Docking positioning at the modelled F508del-CFTR of **7q** (C atom; light green) and **9h** (C atom; gold). The hybrid **9h** ligplot is shown on the right.

Herein, maintaining the m-methoxy substituted phenyl ring in R1 turns into likewise effective compounds, especially in presence of properly substituted phenyl group linked to the position 4 of the main thiazole core, such as H-bonding features as experienced by the p-OCH_3_ substituted analogue **9h** (EC_50_ = 0.12 µM). As shown in Figure 10, the precursor **7q** as well as the newly developed analogue **9h** shared the same positioning at the benzodioxole-carboxamide portion, being H-bonded to V1293 and K1351, while the benzodioxole aromatic group was engaged in π–π stacking and cation–π interaction with F494 and K1351, respectively. On the other hand, the R substituent of **9h** displayed one H-bond with the key residue W1063, thanks to the additional p-OCH_3_ group in R, while the methoxy moiety at the **7q** benzoyl substituent was H-bonded to F494. In any case, the driving force guiding for the two-compound docking mode was thought to be featuring hydrophobic and π–π contacts with F494 and W1063.

The beneficial role played by H-bonding moieties was confirmed focusing on the most promising substitutions applied at the R substituent, when in the presence of the p-SCH_3_ benzoyl group at the position 5 of the thiazole, as shown by the previous analogue **7a** (EC_50_ = 0.10 µM). Indeed, compound **9d** (EC_50_ = 0.10 µM) and **9y** (EC_50_ = 0.076 µM), bearing a p-OCH_3_ or a p-methyl ester phenyl in R, were endowed with comparable or higher potency then the precursor **7a**. As shown in Figure S5, the derived best scored docking poses for all of them guarantee the proper contacts with the protein region delimited by W1063, V1293, and K1351, exhibiting the most potent **9y** additional H-bonds with I177 and E267. Likewise, choosing the same biaryl motif in R1 as for the previous **7m** (EC_50_ = 0.07 µM), led to compounds displaying adequate corrector ability only when combined with small H-bonding groups at the para position of the phenyl ring as R. Thus, only compounds **9f** (EC_50_ = 0.14 µM) and **9r** (EC_50_ = 0.12 µM) displayed quite as similar potencies, as F508del-CFTR correctors, as **7m**.

**Figure 11:**
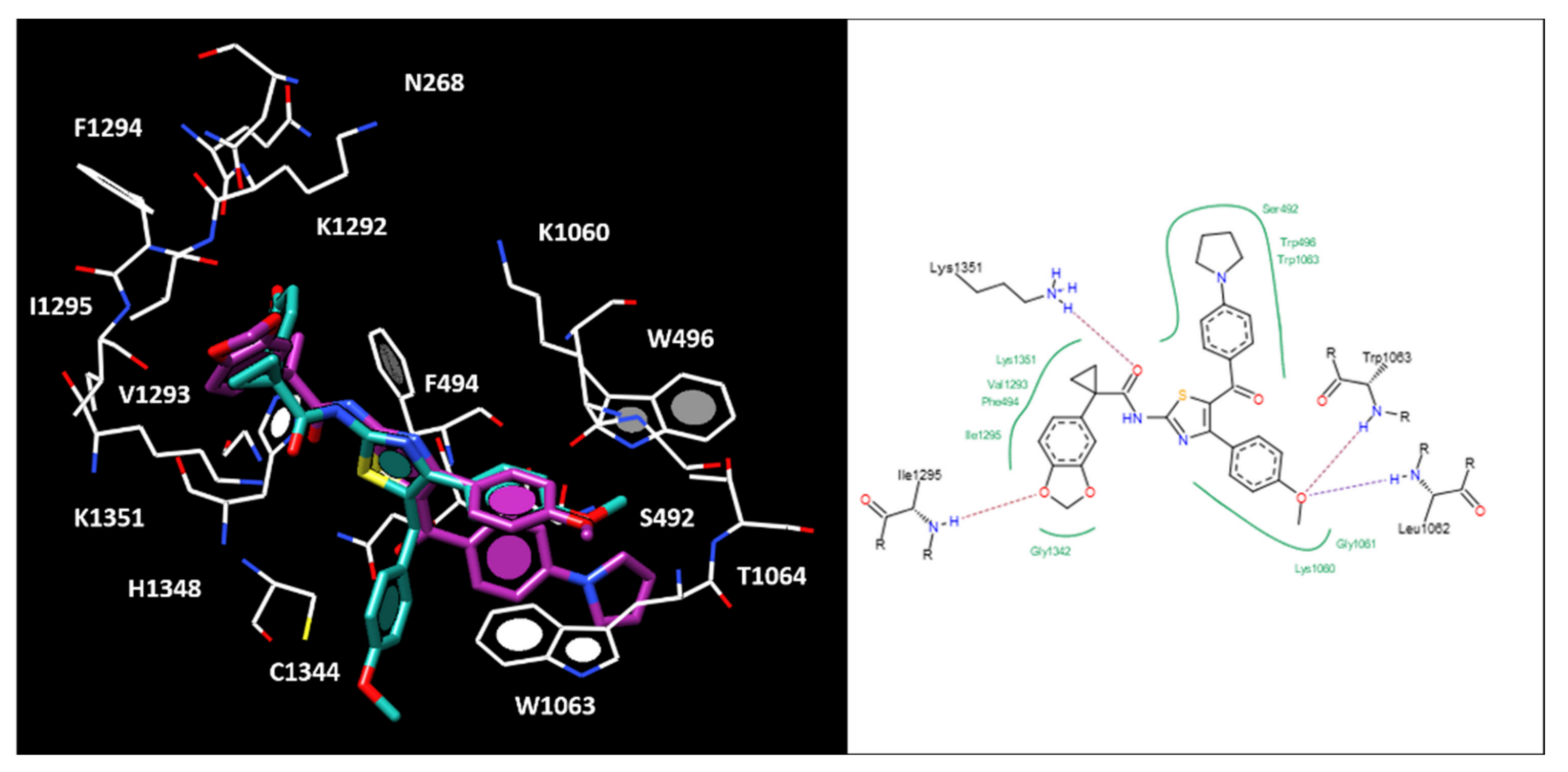
Docking positioning at the modelled F508del-CFTR of 9b (C atom; light sea green) and **9g** (C atom; purple). The hybrid **9g** ligplot is shown on the right.

In accordance with these data, the most promising derivatives herein disclosed featured a methoxy substituted phenyl ring tethered at the position 4 of the main thiazole, as described for compounds **9b** (EC_50_ = 0.064 µM), **9g** (EC_50_ = 0.033 µM), and **9j** (EC_50_ = 0.037 µM). In details, compounds **9b** and **9g**, displaying the p-OCH_3_-phenyl substituent in R, moved this portion of the corrector towards the same crevice of the protein, interacting with the surrounding residues W1063. While both of them maintained the key contacts with K1351 thanks to the carboxamide group, **9b** and **9g** were H-bonded by the benzodioxole oxygen atoms to V1293 and I1296, respectively (Figure 11).

In addition, the most potent **9g** featured further H-bonds with L1062 by means of the R methoxy group. It should be noticed that the bulkier R1 group of **9g** was projected towards the deeper crevice delimited by W496, K1060, and W1063, featuring π–π stacking, while the smaller substituent in R1 for the analogue **9b** was oriented between the W1063 and C1344 residues. This was in agreement with our previous results supporting for hydrophobic contacts with W1063.

As regards **9j**, the introduction of the methoxy group at the meta position of the R phenyl ring moved the compound in proximity of T1064, detecting one H-bond with the protein, while the p-OCF3 phenyl ring included in R1 experienced the aforementioned π -π stacking with W1063 (see Figure S6). However, this docking mode allowed the compound to properly mimic the positioning discussed for **9g**, maintaining the main contacts previously stated for the benzodioxole and carboxamide moieties with I1295 and K1351, then displaying comparable potency values (**9j**, EC_50_ = 0.037 µM; **9g**, EC_50_ = 0.033 µM).

### 2.6. Pharmacophore Modelling

The design, chemical synthesis, and biological evaluation of the newly described hybrids (third series) allowed the structural variations featured by the main thiazole core to be optimized and, conceivably, the SAR of this chemo-type as CFTR modulators to be enlightened. In addition, by collecting about eighty hybrids within all three series of derivatives (see the molecular structure as SMILE format in Table S4) we proceeded with the development of a common pharmacophore model. In order to explore the specific requirements leading to more effective compounds endowed with promising CFTR corrector ability, we focused on those hybrids exhibiting pEC50 values >6.50 M. This threshold was selected in order to guarantee the key information turning into compounds whose potency could be comparable or higher than that of VX-809 (pEC50 = 5.59 M).

About thirty compounds over the collected eighty analogues fulfilled the above-described feature, as shown in Table S5. Interestingly, with the exception of the first best ranked hybrid (**7j**; pEC_50_ = 7.77 M), the novel compounds **9g**, **9j,** and **9b** (pEC_50_ = 7.19–7.48 M) are the most potent of all the three series of derivatives.

Then, by a perspective of the best ranked compounds shown in Table S5, hybrids belonging to the first, second, and third series represent about the 6%, the 41% and the 53% of the collected most potent thirty-two correctors, respectively. Notably, this information provides an important validation of the applied journey and structural variations within hybrids, leading to improved corrector ability, which was especially experienced by the novel derivatives.

Based on these data, the pharmacophore model was built taking into account the aforementioned best ranked thirty-two compounds, featuring the main effective chemical substitutions so far applied within all the three series. Alignment of the derivatives is reported in Figure S7, with **9g** being highlighted as the most potent hybrid of the new third series herein exploited for the development of the pharmacophore analysis.

This model was calculated by means of the pharmacophore search tool by MOE software identifying the most recurrent pharmacophore features exhibited by the collected set of molecules. Any pharmacophore moiety is recognized by an identification code (ID), the percentage by which this feature is shared by the set molecules (SCORE), by a radius that represents the maximum space within which this moiety can be placed with respect to the ligand (RADIUS), and by a symbol explaining the interaction with the biological target (EXPRESSION).

As reported in Table 3, at least 80% of the collected most potent hybrids shared nine pharmacophore features turning into compounds endowed with F508del-CFTR corrector ability, especially aromatic features exhibiting H-bonding groups.

**Figure 12:**
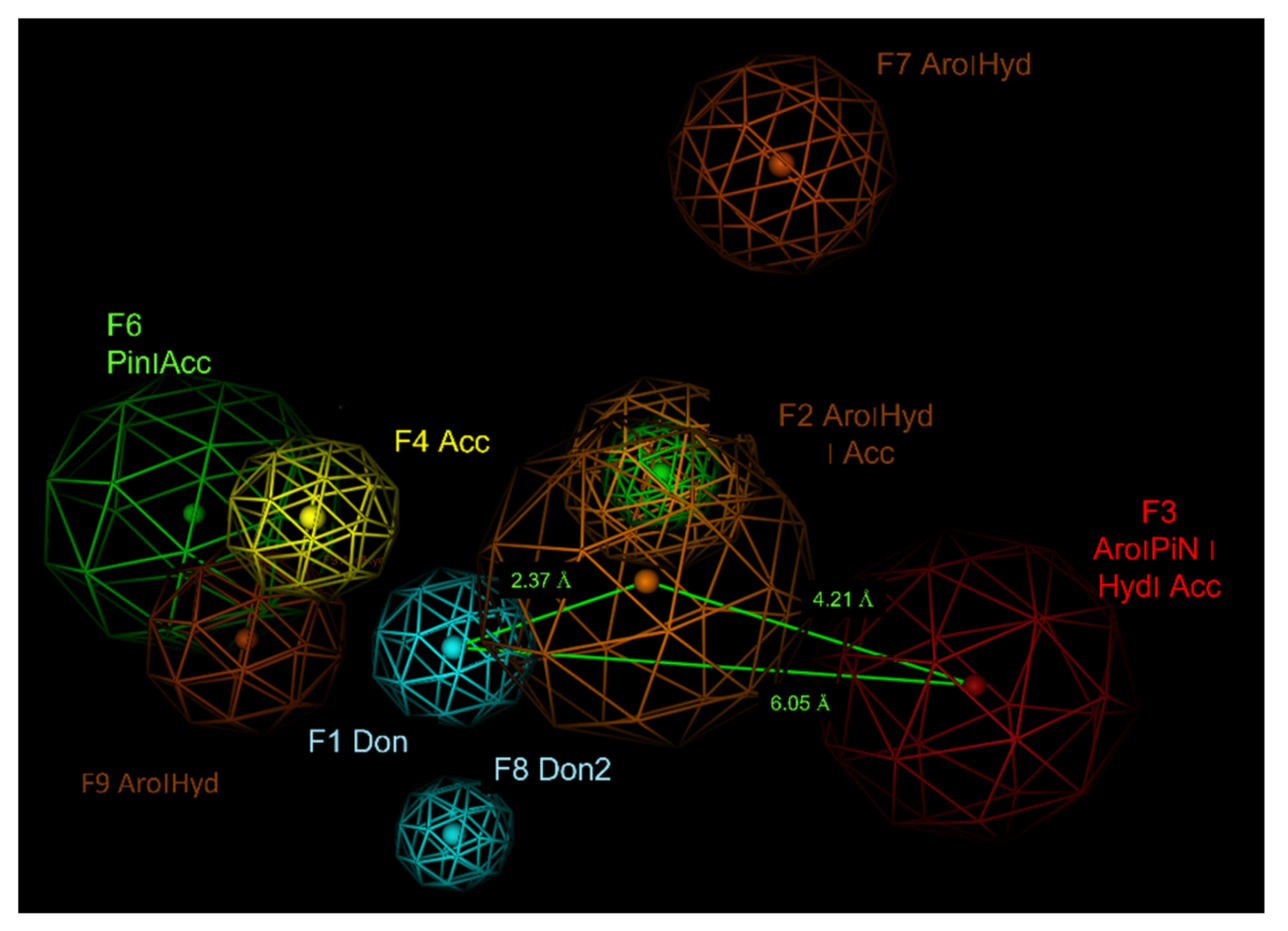
The main pharmacophore requirements experienced by hybrids are reported along with their related distances, focusing on F1–F3 groups. H-bonding groups are named as F Acc and F Don while aromatic features are reported as F Aro.

As shown in Figure 12, the developed model reveals bulky (hetero)aromatic rings including H-bonding function (namely, F2: Aro|Hyd|Acc and F3:Aro PiN|Hyd|Acc), as the thiazole core and the substituent at the thiazole position 4, respectively (see Figure S7).

In addition, the presence of F6:Pin|Acc, F9:Aro|Hyd and of F4:Acc, F1–F2 Don stands for the bicyclic benzodioxole group and the carboxamide function of the well-known corrector VX-809. Then, the F7 Aro|Hyd represents the role played by the substituent at the position 5 of the main thiazole core (see Figure S7).

The suggested reciprocal distances between all the F1–F9 groups exhibited by the collected hybrids highlighted useful requirements for the further screening of novel compounds, conceivably acting as F508del-CFTR correctors.

Indeed, the H-bond donor group exemplified by F1:Don should be at 2.37 Ǻ and 6.05 Ǻ from the main heterocyclic ring represented by F2:Aro|Hyd|Acc and by the terminal aromatic core F3:Aro|PiN|Hyd|Acc, enriched of H-bond acceptor moieties (see the previous Figure 12). The two aromatic groups F2:Aro|Hyd|Acc and F3:Aro|PiN|Hyd|Acc are placed at 9.06 Ǻ to each other, giving some hints for the design of further chemotypes, in place of the phenyl thiazole ring, endowed with the same properties.

**Figure 13:**
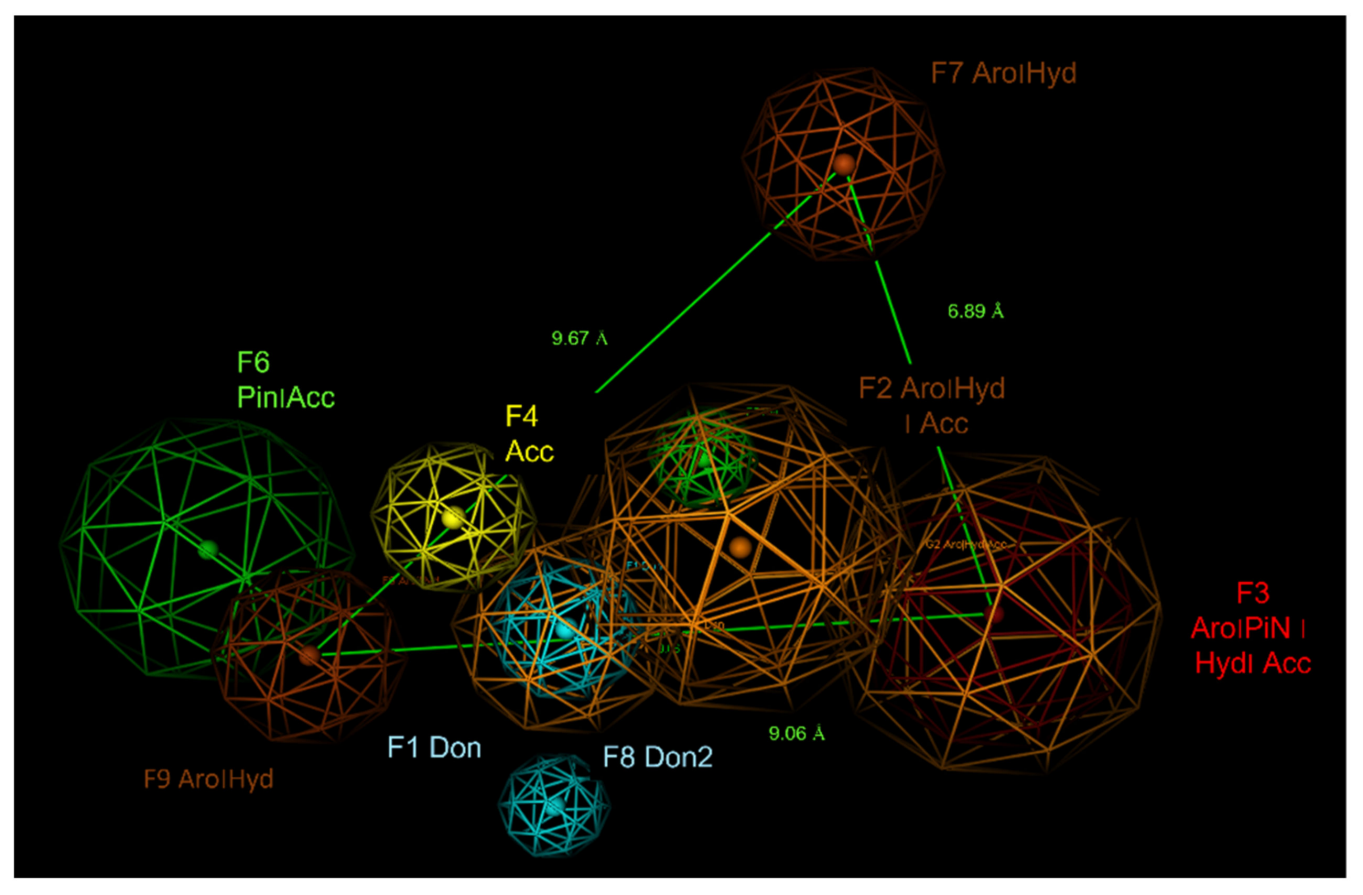
The main pharmacophore requirements experienced by hybrids are reported along with their related distances, focusing on F3, F7, andF9 groups. H-bonding groups are named as F Acc and F Don while aromatic features are reported as F Aro.

Furthermore, the terminal F3:Aro|PiN|Hyd|Acc should be oriented at 6.89 Ǻ and 9.06 Ǻ far from the F7:Aro|Hyd and F9:Aro|Hyd, respectively (see Figure 13). On the other hand, the F7:Aro|Hyd and F9:Aro|Hyd should be placed at 9.67 Ǻ to each other (see Figure 13).

These data once again suggest for proper branched chemo-types involving the benzodioxole group of hybrids (represented by F6:Pin|Acc, F9:Aro|Hyd) as tethered to terminal aromatic or hydrophobic features (F7:Aro|Hyd) as well as to H-bonding groups (F3:Aro|PiN|Hyd|Acc).

Interestingly, the derived model properly matched also the main pharmacophore features shown by the structurally different corrector VX-661 (see Figure S8) revealing: (i) the substituted indole ring of VX-661 as bioisostere of the hybrid benzoyl-thiazole portion, (ii) the diol moiety of VX-661 as effective substitute of the hybrid **9g** p-methoxy phenyl ring, and of (iii) the VX-809 carboxylic moiety.

### 2.7. In Silico Evaluation of Hybrid Pharmacokinetic Properties

Drug discovery strategies recently deeply relied on predictive tools for the in silico evaluation of absorption, distribution, metabolism, and excretion properties (ADME) of novel compounds. Performing computational methods focused on prediction of the pharmacokinetic (PK) profile of derivatives could accelerate the hit-to-lead step and optimization one [37].

Accordingly, herein, we applied a preliminary in silico evaluation of the main PK features explaining the drug-like properties of a number of hybrids, chosen as representative of the three series from the complete library, as reported in Table 4.

Thus, we evaluated the main descriptors analyzed within the Lipinski’ rule [38] and Veber’ rule [39] including the logarithmic ratio of the octanol–water partitioning coefficient (cLogP), the molecular weight (MW) of derivatives, their H-bonding acceptor number (HBA), of donor moieties (HBD), the number of rotatable bonds (nRot_bond). In addition, evaluation of ADME properties was also performed about human intestinal absorption (HIA), volume of distribution (Vd), evaluation of the plasmatic protein binding (%PPB), and evaluation of ligand affinity toward human serum albumin (LogKa HSA). These calculations were made to assess the putative oral bioavailability in terms of percentage (%F).

Based on the performed PK features prediction, with the exception of the reference compounds VX-809, the two correctors VX-661 and VX-445 displayed at least one violation of the Lipinski’s rule or of Veber’s rule, reporting high MW values. On the other hand, all the three known correctors are endowed with high plasmatic protein binding values. Despite this, all of them proved to be exploited for CF therapy featuring somewhat beneficial effects for the patient.

Similarly, most of the developed hybrids showed quite high MW and protein binding values. On the contrary, for most of them, the predicted Vd and cLogP values proved to be more promising than those of the reference correctors.

However, the bioavailability percentage was quite variable within the first series of hybrids (F% = 56–99%, see Table 4), being the prototype **2a** (F% = 56%) less promising than the analogues featuring the ester moiety (**2b–2d**, F% = 69.7–71.8%) or the methyl group (**5c**, F% = 98.6%) at the thiazole position 5. This made the development of further optimized **2a** analogues an urgent need in order to improve, at the same time, the potency, as F508del-CFTR correctors, as well as the hybrid bioavailability. Notably, the development of the second and third series of hybrids led to the design of more potent (**9g**; F% = 64.6%) and drug-like analogues (**7n** and **9g**; (F% = 56–68.1%), exhibiting the pyrrolidine-containing benzoyl substituent as the better choice to foster the effectiveness of both the two **2a** analogues. Acceptable bioavailability values were also featured by **9b**, **9j,** and **9q** (F% = 61.3–61.4%), maintaining a methoxy-substituted phenyl ring at the thiazole position 4, as the previous most active **9g**. Interestingly, this opened the possibility for a further development of new hybrids combining (i) the previously cited pyrrolidine-containing benzoyl substituent at the thiazole position 5 with further electron-rich groups at the thiazole position 4, and (ii) the methoxy-substituted phenyl ring at the thiazole position 4 with a methyl or ester group involving the thiazole position 5.

## 3. Discussion

In this paper, we utilized a multi-disciplinary strategy to determine a third series of derivatives containing promising F508del-CFTR corrector activity. The molecular docking studies carried out on precursor **2a** revealed the main key feature involved in corrector binding, leading the design of this new series of hybrids characterizing structural modifications of the prototype **2a**. Based on computational predictions of the docking mode experienced by the precursor previously described as hybrids (second series), the most promising analogues have been herein conceived and synthesized, taking into account specific substitutions at the main thiazole position 4. All of them have been evaluated as correctors of F508del-CFTR trafficking defect in order to confirm their F508del-CFTR rescue ability and then explored in silico by further molecular docking studies. In particular, molecular docking studies supported the SAR observed within most promising derivatives herein disclosed, bearing a methoxy substituted phenyl ring as R substituent, as described for compounds **9b** (EC_50_ = 0.064 µM), **9g** (EC_50_ = 0.033 µM), and **9j** (EC_50_ = 0.037 µM).

Indeed, molecular modeling analyses suggested once again the pivotal role determined by lipophilic substituents onto the position 5 of the main thiazole ring, in order to properly interact with F494, W496, and W1063. On the other hand, H-bonding K1351 and I1295 also turn into F508del-CFTR corrector activity. In addition, the most promising hybrids herein identified were endowed with further H-bonding motifs at the R substituents, gaining often additional polar contacts with the key residue W1063.

Furthermore, the development of the pharmacophore model allowed the specific requirements to be explored, leading to more effective compounds endowed with promising CFTR corrector ability. The results supported the data obtained by molecular docking studies, underlining the effectiveness of coupling (especially *para*) substituted phenyl ring at the thiazole position 4, with H-bonding groups, with *p*-substituted benzoyl moieties at the position 5. In particular, this substituent should include a somewhat hydrophobic portion enriched with H-bond acceptor features. Accordingly, small groups such as alkoxy, pyrrolidine, or trifluoromethyl substituents were effective (see the chemical structure of **7j**, **9g,** and **9j**), as was the phenyl ring. Indeed, it is thought that this kind of substituent tethered to the thiazole position 5 should be projected within a pocket delimited by aromatic residues, such as W496 and W1063 (see the previous Figure 11).

These promising compounds **9g** and **9j** have been explored in biological studies including by YFP functional assays and short-circuit current measurements in Ussing chamber on primary bronchial epithelia, in order to demonstrate their effectiveness. To complete the study, we considered rescuing the processing defect biochemically by observing the electrophoretic mobility of CFTR protein. In particular, treatment with the active compounds resulted in an increase in the C band/B band ratio, similar to that obtained in the same conditions with VX-809. Lastly, we then investigated the possible corrector combinations using our hybrids and VX-445. In accordance with its functional classification as a type III corrector, VX-445 led to significant additive/synergistic effects when combined with VX-809, so we evaluated the effect of double corrector treatment on the rescue of F508del-CFTR by VX-445 in the presence of our correctors.

In silico prediction of ADME properties confirmed the beneficial role played by the development of further two series of hybrids beyond the synthesis of the prototype **2a**, in order to improve the potency and bioavailability of the new derivatives, as confirmed by compound **9g**, giving also new hints for the further design of new analogues.

Our approach enlightened the effectiveness of VX-809-like derivatives, exhibiting the thiazole main core, as endowed with F508del CFTR corrector ability. To conclude, these data allowed the identification of three molecules (**9b, 9g,** and **9j**), useful as novel CFTR correctors with a good efficacy in rescuing the defect of F508del-CFTR.

## 4. Materials and Methods

### 4.1. Chemistry

#### 4.1.1. Experimental Instrumentation

All solvents and chemicals were reagent grade. Unless otherwise mentioned, all solvents and chemicals were acquired from Alfa Aesar (Kandel, Germany), Sigma Aldrich (St. Louis, MO, USA), VWR(Radnor, PA, USA and Zentek (Milano, Italy) and used as received unless purification was required.

A rotary evaporator allowed solvent removal at ca. 10–50 Torr. The analytical instrument used was an Agilent (Santa Clara, CA, USA) 1260 high performance liquid chromatography (HPLC). The analytical HPLC column was a Phenomenex (Bologna, Italy) C18 Luna (4.6 × 250 mm, 5 µm).

The preparative HPLC was Agilent 1260 Infinity preparative HPLC and the column Phenomenex C18 Luna (21.2 × 250 mm, 15 µm) was used for preparative chromatography. Liquid chromatography–electrospray mass spectrometry (HPLC-ESI-MS) was used to analyze the intermediates and the raw products with an Agilent 1100 series LC/MSD ion trap instrument.

HRMS experiments were performed using Q Exactive Orbitrap instrument by Thermo Scientific (Waltham, MA, USA).

GC analyses were performed using HP5890 series II gas chromatograph coupled to a HP5972 mass spectrometer equipped with an electron impact ionization source (Hewlett-Packard, Palo Alto, CA, USA).

The nuclear magnetic resonance (NMR) analyses were performed using a Varian (Palo Alto, CA, USA) Gemini spectrometer 200 MHz.

The proton spectra and the carbon spectra were acquired at room temperature, at 200 MHz and at 50 MHz, respectively. Chemical shifts are reported in δ units (ppm) relative to TMS as an internal standard. Coupling constants (J) are reported in Hertz (Hz).

All the raw products were purified with preparative HPLC using the following gradient: from 0 to 5 min at 20% eluent B, from 5 min to 40 min to 100% eluent B, and from 40 to 45 min at 100% eluent B. Eluent A was water with 0.1% formic acid (FOA) and eluent B was acetonitrile with 0.1% FOA. All the final products utilized in biological assays were judged to have a purity of 95% or higher, based on analytical HPLC/MS analysis.

Compound purity was determined by integrating peak areas of the chromatogram obtained in liquid phase, monitored at 254 nm.

#### 4.1.2. General Procedure for the Synthesis of Structures

1-(benzo[d][1,3]dioxol-5-yl)-*N*-(5-benzoyl-4-(4-methoxyphenyl)thiazol-2-yl)cyclopropane-1-carboxamide (9a).

4-methoxybenzoyl chloride (270.8 µL, 2 mmol) was dissolved in acetone and at this solution was added ammonium thiocyanate (304.4 mg, 4 mmol) at T = 0 °C. After two hours, bis(4-methoxybenzyl)amine (620.0 mg, 2.4 mmol), obtained by 4-(methoxy)benzaldehyde and (4-methoxyphenyl)methanamine as already described [28], was added to the mixture and the reaction was stirred at room temperature. After completion of the reaction, as monitored by HPLC, acetone was removed by rotavapor and to the residue was added ethyl acetate (EtOAc) and H_2_O. The organic layers were washed with water (3 × 3.5 mL), dried over anhydrous Na_2_SO_4_ and filtered. Ethyl acetate was removed under reduced pressure to give *N*-(bis(4-methoxybenzyl)carbamothioyl)-4-methoxybenzamide (720.0 mg, 80%) as yellow oil, which was used in the next step without further purification.

2-bromo-1-phenylethan-1-one (39.8 mg, 0.2 mmol) was dissolved in *N*,*N*-dimethylformamide (DMF) and *N*-(bis(4-methoxybenzyl)carbamothioyl)-4-methoxybenzamide (90.1 mg, 0.2 mmol) was added; the reaction was heated to 85 °C for 2 h. The mixture was cooled to room temperature, and then ethyl acetate and water were added. The organic phase was washed with brine, dried over anhydrous Na_2_SO_4_ and filtered. After removing solvent in a vacuum, the product obtained was resuspended in trifluoroacetic acid (TFA) (4 mL) and stirred at T = 100 °C for 48 h. Most of TFA was evaporated by using rotavapor and a solution of NaHCO_3_ 1N was added to neutralize the residue. Then the mixture was extracted by ethyl acetate (3 × 5 mL), the organic phase was dried over anhydrous Na_2_SO_4_, filtered and concentrated to afford (2-amino-4-(4-methoxyphenyl)thiazol-5-yl)(phenyl)methanone.

(2-amino-4-(4-methoxyphenyl)thiazol-5-yl)(phenyl)methanone was crystallized in acetonitrile to give a pure product (45.3 mg, 73%).

A solution of benzo[1,3]dioxol-5-yl-cyclopropanecarboxylic acid (21.0 mg, 0.1 mmol), HATU (34.2 mg, 0.09 mmol) and DIPEA (38 µL, 0.1 mmol) was dissolved in anhydrous DMF and stirred at room temperature for 5 min. Then, (2-amino-4-(4-methoxyphenyl)thiazol-5-yl)(phenyl)methanone (31.0 mg, 0.1 mmol) in anhydrous DMF was added and the mixture was heated to 50 °C for 24 h.

Preparative HPLC was used to purify the product and after concentration of the peak of interest, the final product was obtained with a purity higher than 95%. The purity of the compound was determined by HPLC-MS. Then the product was lyophilized and afforded as a powder (12.5 mg, 25%).

^1^H NMR (200 MHz, DMSO-d6): δ 12.01 (s, 1H, broad, NH); 7.72–6.79 (m, 12H, arom); 6.02 (s, 2H, OCH_2_O); 3.64 (s, 3H, OCH_3_); 1.63–1.44 (m, 2H, cyclopr); 1.38–1.11 (m, 2H, cyclopr).

^13^C NMR (50 MHz, DMSO-d6): δ 188.2, 171.8, 159.8, 159.1, 153.8, 146.8, 146.3, 138.5, 131.8, 130.6, 128.8, 126.4, 123.2, 121.1, 118.4, 113.1, 112.6, 110.2, 107.8, 100.6, 54.6, 30.2, 15.4.

HRMS (ESI) calculated for C_28_H_23_N_2_O_5_S: [M + H]^+^ 499.13276; found 499.13264.

1-(benzo[d][1,3]dioxol-5-yl)-N-(5-(4-methoxybenzoyl)-4-(3-methoxyphenyl)thiazol-2-yl)cyclopropane-1-carboxamide (**9i**).

Ammonium thiocyanate (152.2 mg, 2 mmol) was added to a solution of 3-methoxybenzoyl chloride (141.0 µL, 1 mmol) in acetone (1.5 mL) and the mixture was stirred at T = 0 °C for 2 h.

To the suspension was added bis(4-methoxybenzyl)amine (308.0 mg, 1.2 mmol ) and the reaction was stirred at room temperature. After completion, acetone was removed in vacuum; the residue was added to EtOAc and water. The organic phases were washed with H_2_O (3 × 3 mL), then dried over anhydrous Na_2_SO_4_, filtered and evaporated to afford *N*-(bis(4-methoxybenzyl)carbamothioyl)-3-methoxybenzamide (347.0 mg, 77%) as yellow oil, which was used in the next step without further purification.

2-bromo-1-(4-methoxyphenyl)ethan-1-one (45.0 mg, 0.2 mmol) was dissolved in DMF and N-(bis(4-methoxybenzyl)carbamothioyl)-3-methoxybenzamide (90.1 mg, 0.2 mmol) was added; the reaction was heated to 85 °C for 3 h. The mixture was cooled to room temperature and was extracted by ethyl acetate and washed with H_2_O (3 × 3 mL). The organic layer was dried over anhydrous Na_2_SO_4_ and filtered. Ethyl acetate was removed by rotavapor. The compound was resuspended in TFA (about 5 mL) and heated to 100 °C until complete deprotection (36 h). Most of TFA was evaporated in vacuum, then, a solution of NaHCO_3_ 1N was added to the residue to neutralize the acid and the product was extracted with EtOAc (3 × 5 mL). The organic phase was washed with brine, dried over anhydrous Na_2_SO_4_ and filtered. After concentration, (2-amino-4-(3-methoxyphenyl)thiazol-5-yl)(4-methoxyphenyl)methanone was crystallized in acetonitrile (47.2 mg, 69%).

Benzo[1,3]dioxol-5-yl-cyclopropanecarboxylic acid (21.0 mg, 0.1 mmol) was dissolved in anhydrous DMF (1 mL) with DIPEA (38 µL, 0.1mmol) and HATU (34.2 mg, 0.09 mmol). After 5 min, a solution of (2-amino-4-(3-methoxyphenyl)thiazol-5-yl)(4-methoxyphenyl)methanone (35.0 mg, 0.11 mmol) was added portion wise to the mixture. The reaction was heated to 50 °C and stirred until completeness (24 h). The mixture was purified by preparative HPLC to afford the title compound with purity higher than 95%, as confirmed by HPLC-MS. (13.5 mg, 26%).

^1^H NMR (200 MHz, DMSO-d6): δ 11.85 (s, 1H, broad, NH); 7.68–6.86 (m, 11H, arom); 6.01 (s, 2H, OCH_2_O); 3.61 (s, 3H, OCH_3_); 3.58 (s, 3H, OCH_3_); 1.67–1.43 (m, 2H, cyclopr); 1.39–1.11 (m, 2H, cyclopr).

^13^C NMR (50 MHz, DMSO-d6): δ 187.8, 172.4, 159.7, 159.2, 158.4, 153.9, 146.8, 146.3, 138.5, 131.8, 130.5, 128.9, 126.3, 123.2, 121.2, 118.4, 113.2, 112.7, 110.2, 107.8, 100.6, 54.7, 30.2, 15.4.

HRMS (ESI) calculated for C_29_H_25_N_2_O_6_S: [M + H]^+^ 529.14332; found 529.14262

1-(benzo[d][1,3]dioxol-5-yl)-N-(5-(4-methoxybenzoyl)-4-(2-methoxyphenyl)thiazol-2-yl)cyclopropanecarboxamide (**9p**).

2-methoxybenzoyl chloride was synthetized as previously described [40] with some modification. Briefly, to a solution of 2-methoxybenzoic acid (152 mg, 1 mmol) in anhydrous dichloromethane (DCM) (2 mL) were added few drops of DMF and thionyl chloride (198 µL, 2.5 mmol) at T= 0 °C. The reaction was stirred at T = 80 °C for 2 h and then at T = 50 °C overnight. The reaction mixture was concentrated in vacuum to afford the crude 2-methoxybenzoyl chloride, controlled with GC-MS, as yellow oil (150 mg, 88%). A mixture of 2-methoxybenzoyl chloride (68 mg, 0.4 mmol) in acetone (1 mL) and ammonium thiocyanate (60.8 mg, 0.8 mmol) was stirred for about 2 h, at T = 0 °C.

Bis(4-methoxybenzyl)amine (128.5 mg, 0.5 mmol) was added to the reaction and the resulting mixture was stirred for 3 h at room temperature. Acetone was removed by rotavapor and EtOAc and H_2_O were added to the residue. The organic phase was washed with water (3 × 3.5 mL), dried over anhydrous Na_2_SO_4_, filtered, and concentrated under reduced pressure to afford *N*-(bis(4-methoxybenzyl)carbamothioyl)-2-methoxybenzamide (150.0 mg, 83%), a yellow oil, which was used in the next step without further purification.

To a solution of 2-bromo-1-(4-methoxyphenyl)ethanone (56.0 mg, 0.2 mmol) in DMF was added portionwise *N*-(bis(4-methoxybenzyl)carbamothioyl)-2-methoxybenzamide (50.0 mg, 0.2 mmol) and the resulting mixture was stirred at T = 85 °C for 3 h. At room temperature, to the mixture were added EtOAc and H_2_O. The organic layers were washed with brine, dried over anhydrous Na_2_SO_4_, filtered, and concentrated. The residue was stirred in TFA (4 mL) and heated to 100 °C for 48 h. Most of TFA was removed in vacuum and the residue was neutralized using a solution of NaHCO_3_ 1N. The extraction of the compound was performed with EtOAc (3 × 5 mL) and then the layers were washed with brine, dried over anhydrous Na_2_SO_4_, and filtered. After concentration, (2-amino-4-(2-methoxyphenyl)thiazol-5-yl)(4-methoxyphenyl)methanone was used in the next step without purification (45.0 mg, 66%).

Benzo [1,3] dioxol-5-yl-cyclopropanecarboxylic acid (21.0 mg, 0.1 mmol), HATU (34.2 mg, 0.09 mmol), and DIPEA (38 µL, 0.1mmol) were dissolved in 1 mL of anhydrous DMF.

After few minutes, a solution of (2-amino-4-(2-methoxyphenyl)thiazol-5-yl)(4-methoxyphenyl)methanone (34 mg, 0.1 mmol) was added portionwise to the reaction and the resulting mixture was stirred at T = 50 °C (36 h). The purification was made by preparative HPLC. The peak of interest was concentrated to give the final product with purity of >95% as confirmed by HPLC-MS (12.0 mg, 23%).

^1^H NMR (200 MHz, DMSO-d6): δ 11.76 (s, 1H, broad, NH); 7.71–6.88 (m, 11H, arom); 6.02 (s, 2H, OCH_2_O); 3.65 (s, 3H, OCH_3_); 3.61 (s, 3H, OCH_3_); 1.73–1.45 (m, 2H, cyclopr); 1.42–1.09 (m, 2H, cyclopr).

^13^C NMR (50 MHz, DMSO-d6): δ 188.1, 171.7, 159.6, 159.2, 157.9, 153.8, 146.8, 146.2, 138.5, 131.8, 131.0, 128.9, 126.3, 123.5, 123.2, 121.1, 118.4, 113.2, 112.7, 110.2, 107.8, 54.7, 53.9, 30.2, 15.4.

HRMS (ESI) calculated for C_29_H_25_N_2_O_6_S: [M + H]^+^ 529.14332; found 529.14266

Methyl 4-(5-([1,1′-biphenyl]-4-carbonyl)-2-(1-(benzo[d][1,3]dioxol-5-yl)cyclopropane-1-carboxamido)thiazol-4-yl)benzoate (**9r**).

Methyl 4-(chlorocarbonyl)benzoate (198.6 mg, 1 mmol) was dissolved in acetone (1 mL) and ammonium thiocyanate (152.2 mg, 2 mmol) was added at 0 °C; the reaction was stirred for about 2 h, at this temperature.

Bis(4-methoxybenzyl)amine (308.4 mg, 1.2 mmol) was added to the reaction and the resulting mixture was stirred for 2 h, at room temperature. The mixture was concentrated under reduced pressure and the residue was diluted by water and extracted with EtOAc. The organic phase was dried over anhydrous Na_2_SO_4_ and filtered. After concentration, methyl 4-((bis(4-methoxybenzyl)carbamothioyl)carbamoyl)benzoate was afforded (254.0 mg, 53%), as a yellow oil, which was used in the next step without further purification.

A solution of 1-([1,1′-biphenyl]-4-yl)-2-bromoethan-1-one (55.0 mg, 0.2 mmol) and methyl 4-((bis(4-methoxybenzyl)carbamothioyl)carbamoyl)benzoate (95.7 mg, 0.2 mmol) in DMF was stirred at T = 85 °C. After 3 h, the mixture was cooled to room temperature, diluted in water, and extracted with EtOAc (3 × 5 mL). The organic layer was dried over anhydrous Na_2_SO_4_ and filtered. Solvent was removed by rotavapor and the residue was resuspended in TFA (4 mL) and heated to 100 °C for 48 h. The reaction was concentrated by rotavapor and the residue was neutralized preparing a solution of NaHCO_3_ 1N. The mixture was extracted with EtOAc (3 × 5 mL), the organic layers were dried over anhydrous Na_2_SO_4_ and filtered.

After concentration, methyl 4-(5-([1,1′-biphenyl]-4-carbonyl)-2-aminothiazol-4-yl)benzoate was crystallized in absolute ethanol (45.6 mg, 55%).

A mixture of benzo[1,3]dioxol-5-yl-cyclopropanecarboxylic acid (21.0 mg, 0.1 mmol), DIPEA (38 µL, 0.1 mmol) and HATU (34.6 mg, 0.09 mmol) was dissolved in anhydrous DMF.

After few minutes, a solution of methyl 4-(5-([1,1′-biphenyl]-4-carbonyl)-2-aminothiazol-4-yl)benzoate (41.5 mg, 0.1 mmol) was added to the reaction and the resulting mixture was stirred at T = 50 °C for 42 h. To purify the product, preparative HPLC was used and it allowed the title compound with purity higher than 95% to be obtained, as confirmed by HPLC-MS (12.7 mg, 21%).

^1^H NMR (200 MHz, DMSO-d6): δ 11.87 (s, 1H, broad, NH); 7.81–6.63 (m, 16H, arom); 6.02 (s, 2H, OCH_2_O); 4.06 (s, 3H, CH_3_O); 1.72–1.44 (m, 2H, cyclopr); 1.40–1.15 (m, 2H, cyclopr).

^13^C NMR (50 MHz, DMSO-d6): δ 188.2, 172.3, 166.4, 159.6, 159.1, 153.4, 146.9, 146.3, 143.4, 138.5, 136.1, 131.8, 130.5, 129.2, 128.4, 127.9, 126.4, 125.9, 123.2, 121.2, 118.5, 112.8, 110.2, 107.8, 100.6, 52.5, 30.3, 15.5.

HRMS (ESI) calculated for C_35_H_27_N_2_O_6_S: [M + H]^+^ 603.15897; found 603.15834

*N*-(5-([1,1′-biphenyl]-4-carbonyl)-4-(4-(methylthio)phenyl)thiazol-2-yl)-1-(benzo[d][1,3]dioxol-5-yl)cyclopropane-1-carboxamide (9s)

To a solution of 4-methylthiobenzoic acid (80 mg, 0.5 mmol) in anhydrous dichloromethane (DCM) (2 mL) were added few drops of DMF and thionyl chloride (91 µL, 1.25 mmol) at T = 0 °C. The reaction was stirred at T = 80 °C, and then at T = 50 °C overnight. The reaction mixture was concentrated in vacuum to afford crude 4-(methylthio)benzoyl chloride, controlled with GC-MS, as colorless oil (75 mg, 81%).

A mixture of 4-(methylthio)benzoyl chloride (75 mg, 0.4 mmol) in acetone (1 mL) and ammonium thiocyanate (60.8 mg, 0.8 mmol) was stirred for about 2 h, at T= 0 °C.

Bis(4-methoxybenzyl)amine (128.5 mg, 0.5 mmol) was added to the reaction and the resulting mixture was stirred for 3 h at room temperature. The solvent was evaporated by rotavapor and the residue was diluted with water and extract with EtOAc (3 × 5 mL). The organic phase was dried over anhydrous Na_2_SO_4_ and filtered. Solvent was removed in vacuum to give N-(bis(4-methoxybenzyl)carbamothioyl)-4-(methylthio)benzamide (131.0 mg, 70%), a yellow oil, which was used in the next step without further purification.

To a solution of 1-([1,1′-biphenyl]-4-yl)-2-bromoethan-1-one (55.0 mg, 0.2 mmol) in DMF was added portionwise *N*-(bis(4-methoxybenzyl)carbamothioyl)-4-(methylthio)benzamide (93.3 mg, 0.2 mmol) and the resulting mixture was heated to 85 °C. After 2 h the reaction was completed as monitored by HPLC, cooled to room temperature and the mixture was partitioned between EtOAc and H_2_O. The organic layer was washed with brine and dried over anhydrous Na_2_SO_4_. The organic phase was filtered, and ethyl acetate was evaporated by rotavapor. The residue was resuspended in TFA (4 mL) and heated to 100 °C for 48 h. TFA was evaporated in vacuum and the residue was neutralized with a solution of NaHCO_3_ 1N. After having extracted with EtOAc (3 × 3 mL), the organic layers were dried over anhydrous Na_2_SO_4_ and filtered.

After concentration, [1,1′-biphenyl]-4-yl(2-amino-4-(4-(methylthio)phenyl)thiazol-5-yl)methanone was crystallized in acetonitrile (52.3 mg, 65%).

Benzo[1,3]dioxol-5-yl-cyclopropanecarboxylic acid (21.0 mg, 0.1 mmol), HATU (34.2 mg, 0.09 mmol) and DIPEA (38 µL, 0.1 mmol) were resuspended in anhydrous DMF (1 mL) at room temperature. After 5 min, a solution of [1,1′-biphenyl]-4-yl(2-amino-4-(4-(methylthio)phenyl)thiazol-5-yl)methanone (40.0 mg, 0.1 mmol) was added and the resulting mixture was heated to 50 °C for 40 h. Preparative HPLC was used to purify the final product. The peak of interest was concentrated to obtain a powder with a purity higher than >95%, as confirmed by HPLC-MS (13.0 mg, 22%).

^1^H NMR (200 MHz, DMSO-d6): δ 11.89 (s, 1H, broad, NH); 7.85–6.70 (m, 16H, arom); 6.04 (s, 2H, OCH_2_O); 2.48 (s, 3H, SCH_3_); 1.69–1.43 (m, 2H, cyclopr); 1.38–1.09 (m, 2H, cyclopr).

^13^C NMR (50 MHz, DMSO-d6): δ 188.1, 172.1, 159.6, 159.2, 152.8, 146.9, 146.3, 144.2, 138.5, 135.9, 131.8, 130.7, 129.4, 128.6, 127.9, 126.2, 125.8, 123.2, 121.2, 118.4, 114.7, 112.8, 110.2, 107.8, 100.6, 30.3, 14.5.

HRMS (ESI) calculated for C_34_H_27_N_2_O_4_S_2_: [M + H]^+^ 591.141216; found 591.14013.

*N*-(5-([1,1′-biphenyl]-4-carbonyl)-4-(pyridin-3-yl)thiazol-2-yl)-1-(benzo[d][1,3]dioxol-5-yl)cyclopropane-1-carboxamide (**9t**).

A solution of nicotinoyl chloride (141.6 mg, 1 mmol) in acetone (1 mL) was cooled at T = 0 °C, and then ammonium thiocyanate (152.2 mg, 2 mmol) was added and stirred for 2 h, at this temperature.

Bis(4-methoxybenzyl)amine (308.0 mg, 1.2 mmol) was added to the reaction and the resulting mixture was stirred for 2 h at room temperature. Acetone was evaporated under vacuum, the residue was diluted with water and extracted with EtOAc. The organic layers were dried over anhydrous MgSO_4_ and filtered. After concentration, N-(bis(4-methoxybenzyl)carbamothioyl)nicotinamide (312.0 mg, 74%), was afforded as a yellow oil, which was used in the next step without further purification.

A solution of *N*-(bis(4-methoxybenzyl)carbamothioyl)nicotinamide (84.3 mg, 0.2 mmol) and 1-([1,1″-biphenyl]-4-yl)-2-bromoethan-1-one (55.0 mg, 0.2 mmol) in DMF was heated to 85 °C until complete. To the mixture were added EtOAc and H_2_O. The organic layer was dried over anhydrous Na_2_SO_4_ and filtered. After concentration, the residue was dissolved in TFA (4 mL) and heated to 100 °C. After complete deprotection, TFA was removed by rotavapor and to the residue was added a solution of NaHCO_3_ 1N to neutralize the residual acid. The compound was extracted with EtOAc (3 × 5 mL), and then the organic phases were dried over anhydrous Na_2_SO_4_.

After concentration, [1,1′-biphenyl]-4-yl(2-amino-4-(pyridin-3-yl)thiazol-5-yl)methanone was crystallized in acetonitrile (48.6 mg, 68%).

[1,1′-biphenyl]-4-yl(2-amino-4-(pyridin-3-yl)thiazol-5-yl)methanone (35.7 mg, 0.1 mmol) was dissolved in anhydrous DMF and a mixture of benzo[1,3]dioxol-5-yl-cyclopropanecarboxylic acid (21.0 mg, 0.1 mmol), HATU (34.2 mg, 0.09 mmol) and DIPEA (38 µL, 0.1 mmol) in DMF was added to the solution, after 5 min of activation.

The resulting mixture was heated to 50 °C for 48 h. The purification of the final product was made by preparative HPLC to obtain a compound with a purity higher than 95% as confirmed by HPLC-MS (11.0 mg, 20%).

^1^H NMR (200 MHz, DMSO-d6): δ 12.01 (s, 1H, broad, NH); 8.03–6.98 (m, 16H, arom); 6.05 (s, 2H, OCH_2_O); 1.79–1.48 (m, 2H, cyclopr); 1.42–1.12 (m, 2H, cyclopr).

^13^C NMR (50 MHz, DMSO-d6): δ 187.8, 172.2, 159.7, 159.1, 153.4, 149.3, 147.7, 146.3, 138.5, 136.0, 131.9, 131.3, 129.2, 128.7, 127.9, 126.2, 124.0, 123.2, 121.2, 118.4, 112.9, 110.2, 107.8, 100.6, 30.2, 15.5.

HRMS (ESI) calculated for C3_2_H_23_N_3_O_4_S: [M + H]^+^ 545.14091; found 546.14856.

*N*-(5-([1,1′-biphenyl]-4-carbonyl)-4-(4-chlorophenyl)thiazol-2-yl)-1-(benzo[d][1,3]dioxol-5-yl)cyclopropane-1-carboxamide **(9u**).

To a solution of 4-chlorobenzoyl chloride (102.6 µL, 0.8 mmol) in acetone (1 mL) cooled at T = 0 °C, ammonium thiocyanate (121.6 mg, 1.6 mmol) was added and stirred for 2 h at this temperature.

Bis(4-methoxybenzyl)amine (246.7 mg, 0.96 mmol) was added to the mixture and the reaction was stirred for 2 h, at room temperature. Solvent was removed, the residue was diluted with water and then the extraction was made by EtOAc. The organic phase was dried over anhydrous MgSO_4_ and filtered. Solvent was removed by rotavapor to obtain *N*-(bis(4-methoxybenzyl)carbamothioyl)-4-chlorobenzamide (330 mg, 91%) as pale-yellow oil, which was used in the next step without further purification.

1-([1,1′-biphenyl]-4-yl)-2-bromoethan-1-one (55.0 mg, 0.2 mmol) was dissolved in DMF and N-(bis(4-methoxybenzyl)carbamothioyl)-4-chlorobenzamide (91.0 mg, 0.2 mmol) was added; the mixture was heated to 85 °C. After 2 h the solution was diluted with water and extracted with EtOAc (3 × 5 mL). The organic layer was dried over anhydrous Na_2_SO_4_ and filtered. After concentration, the residue was resuspended in TFA (4 mL) and heated to 100 °C until complete deprotection (36 h). After removing TFA by rotavapor, the residue was neutralized with NaHCO_3_ 1N, and then an extraction was made by EtOAc (3 × 5 mL). The organic layers were washed with brine, dried over anhydrous MgSO_4_, and filtered. After concentration, [1,1′-biphenyl]-4-yl(2-amino-4-(4-chlorophenyl)thiazol-5-yl)methanone was crystallized in acetonitrile (32.0 mg, 41%).

Benzo[1,3]dioxol-5-yl-cyclopropanecarboxylic acid (16.5 mg, 0.08 mmol) was dissolved in anhydrous DMF (1 mL) with DIPEA (31 µL, 0.08 mmol) and HATU (27.4 mg, 0.07 mmol).

After few minutes, a solution of [1,1′-biphenyl]-4-yl(2-amino-4-(4-chlorophenyl)thiazol-5-yl)methanone (32.0 mg, 0.08 mmol) was added to the solution and the reaction was heated to 50 °C until complete (36 h). The final product was purified by preparative HPLC. The peak of interest was concentrated and lyophilized to obtain the title compound as a powder with purity of >95% as confirmed by HPLC-MS (14.8 mg, 32%).

^1^H NMR (200 MHz, DMSO-d6): δ 11.98 (s, 1H, broad, NH); 7.73–6.81 (m, 16H, arom); 6.01 (s, 2H, OCH_2_O); 1.64–1.40 (m, 2H, cyclopr); 1.38–1.12 (m, 2H, cyclopr).

^13^C NMR (50 MHz, DMSO-d6): δ 187.9, 172.4, 159.9, 152.7, 146.8, 146.3, 143.6, 138.4, 136.0, 132.9, 132.6, 131.7, 130.7, 129.4, 128.6, 127.9, 127.4, 126.5, 125.9, 124.5, 123.2, 110.2, 107.8, 100.6, 30.2, 15.5.

HRMS (ESI) calculated for C_33_H_24_ClN_2_O_4_S: [M + H]^+^ 579.11452; found 579.11385.

### 4.2. Computational Studies

All the herein explored compounds were manually built by the MOE Builder module of the MOE software (V.MOE2019) and then were parametrized (AM1 partial charges as calculation method). Then, they were energy minimized by the Energy Minimize tool, using MMFF94x forcefield and RMS (root mean square) equal to 0.0001 Kcal/mol/A^2^ to produce a single local low-energy conformation for each ligand.

Docking calculations within the previously modelled F508del CFTR protein [29] were performed by means of the LeadIT 2.1.8 software suite (www.biosolveit.com) accessed on 31 January 2022, relying on the FlexX scoring algorithm. This software runs on in silico evaluation of the binding free energy applying the Gibbs–Helmholtz equation [41,42,43]. The software identifies the binding site by a radius of 6 Å far from the previously investigated compound **2a**, in order to further proceed with the setup of a spherical search space for the following docking calculation. The 7-compound series as well as the novel synthesized 9-compound series was evaluated applying the standard setting as docking protocol. Thus, the Hybrid Approach (enthalpy and entropy criteria) was followed, whose related scoring function role is reported in the literature [44]. The obtained docking conformers were prioritized by specific scores values based on thin terms of lowest energy pose of the derivatives docked to the protein model. All ligands were then optimized and rescored by assessment with the HYDE algorithm, included in the LeadIT 2.1.8 software. The HYDE tool is based on dehydration enthalpy and hydrogen bonding as shown in the literature [45,46].

The pharmacophore model was obtained thanks to the pharmacophore search module included in MOE software. The corresponding pharmacophore consensus tool led to a set of recommended properties based on the proposed alignment of compounds. These are classified by a position, radius, and a type expression. Details of the applied method are reported in our previous works [47,48].

The prediction of ADME properties was made based on the Advanced Chemistry Development (ACD) Percepta platform This software works relying on the implemented training libraries, which include different series of ligands in tandem with their experimentally explored pharmacokinetic properties.

### 4.3. Biological Evaluations

#### 4.3.1. Cell Culture

Immortalized bronchial epithelial CFBE41o-cells with stable co-expression of the halide-sensitive yellow fluorescent protein (HS-YFP) and of F508del-CFTR were cultured using MEM medium supplemented with 10% fetal calf serum, 100 U/mL penicillin, 2 mM L-glutamine, and 100 mg/mL streptomycin.

The protocols to isolate, culture, and differentiate primary bronchial epithelial cells were previously detailed [49]. In brief, epithelial cells were obtained from mainstem human bronchi of CF individuals who had undergone lung transplantation. For the present study, cells from two F508del homozygous CF patients (HBE73 and HBE93) were utilized. Epithelial cells were cultured in a serum-free medium (LHC9 mixed with RPMI 1640, 1:1) enriched with several hormones and supplements to promote cell number expansion. To eradicate bacteria, the culture medium also contained a mixture of antibiotics (usually colistin, piperacillin, and tazobactam) for the first 3–5 days of culture. The collection of bronchial epithelial cells (supported by Fondazione per la Ricerca sulla Fibrosi Cistica through the “Servizio Colture Primarie”) and their study to investigate the mechanisms of transepithelial ion transport were specifically approved by the Ethics Committee of the Istituto Giannina Gaslini following the guidelines of the Italian Ministry of Health (registration number: ANTECER, 042-09/07/2018). Each patient provided informed consent to the study using a form that was also approved by the Ethics Committee. Fully-differentiated epithelia were generated by seeding cells at high density on porous membranes (Snapwell inserts, Corning Life Sciences, Acton, MA, USA, code 3801). The following day, the serum-free medium was removed from both sides and, on the basolateral side only, replaced with Pneumacult ALI medium (StemCell Technologies, Vancouver, BC, Canada). Epithelia were cultured in air–liquid interface (ALI) condition for up to 20 days.

#### 4.3.2. Antibodies

The following antibodies were used: mouse monoclonal anti-CFTR (570 and 596) provided through a program of the Cystic Fibrosis Foundation by J.R. Riordan [50], mouse monoclonal anti-GAPDH (clone 6C5, Santa Cruz Biotechnology, Dallas, TX, USA); horseradish peroxidase (HRP)-conjugated anti-mouse IgG (Abcam, Cambridge, UK); or HRP-conjugated anti-rabbit IgG (DAKO, Santa Clara, CA, USA).

#### 4.3.3. Fluorescence Assay for CFTR Activity

CFBE41o-cells co-expressing mutants CFTR and HS-YFP were plated (50,000 cells/well) on clear-bottom 96-well black microplates (Corning Life Sciences, Acton, MA) and grown at 37 °C in 5% CO_2_ for 24 h. For the corrector assay, CFBE41o-cells were treated for further 24 h with compounds as indicated. The following day, prior to the assay, the culture medium was removed and cells were stimulated for 30 min at 37 °C with 60 µL PBS (containing 137 mM NaCl, 2.7 mM KCl, 8.1 mM Na_2_HPO_4_, 1.5 mM KH_2_PO_4_, 1mM CaCl_2_, and 0.5 mM MgCl_2_) plus forskolin (20 µM) and VX-770 (1 µM).

Microplates containing CFBE41o-cells were then transferred to a plate reader (FluoStar Galaxy; BMG Labtech, Offenburg, Germany) equipped with excitation (HQ500/20X: 500 ± 10 nm) and emission (HQ535/30M: 535 ± 15 nm) filters for YFP (Chroma Technology, Brattleboro, VT, USA). During the assay fluorescence was measured continuously for 14 s with 2 s before and 12 s after injection of an iodide containing solution (165 µL of a modified PBS containing I^-^ instead of Cl^-^; final I^-^ concentration in the well: 100 mM).

Fluorescence data were normalized for the initial value and fluorescence quenching rate following I^-^ influx, were determined by fitting the final 10 s of data for each well with an exponential function to extrapolate initial slope (dF/dt).

To evaluate the dose–response relationships, activity slopes obtained for each compound at the different concentrations were fitted with the Hill equation using the Igor software (WaveMetrics) to extrapolate EC_50_, maximal effect, and Hill coefficient.

#### 4.3.4. Western Blot Analysis of CFTR Expression Pattern

CFBE41o-cells treated with vehicle alone (DMSO), or with VX-809, or with test compounds (at the desired concentrations) were grown to confluence on 60 mm diameter dishes and lysed in RIPA buffer containing a complete protease inhibitor (Roche, Basel, Switzerland). Cell lysates were subjected to centrifugation at 15,300× *g* at 4 °C for 10 min.

Supernatant protein concentration was calculated using the BCA assay (Euroclone, Milan, Italy). For each sample, 10 μg of total proteins was resolved in 4–20% gradient Criterion TGX precast gels, transferred to nitrocellulose membranes (using a Trans-Blot Turbo system; Bio-Rad, Hercules, CA, USA), and analyzed by Western blotting. Proteins were detected using the antibodies described above and subsequently visualized by chemiluminescence using the SuperSignal West Femto Substrate (Thermo Fisher Scientific, Waltham, MA, USA). A Molecular Imager ChemiDoc XRS System was used to monitor chemiluminescent signals. Images were analyzed with ImageJ software (NIH). Bands were analyzed as ROI, normalized against the GAPDH loading control.

#### 4.3.5. Short-Circuit Current Recordings

Differentiated bronchial epithelia grown on snapwells were mounted in a vertical diffusion Ussing chamber with internal fluid circulation. Both hemichambers were filled with a solution containing (in mM): 126 NaCl, 0.38 KH_2_PO_4_, 2.13 K_2_HPO_4_, 1 MgSO4, 1 CaCl_2_, 24 NaHCO_3_, and 10 glucoses, and continuously bubbled with a 5% CO_2_–95% air mixture. The temperature was kept at 37 °C. The transepithelial voltage was short-circuited with a voltage-clamp (DVC-1000, World Precision Instruments, Sarasota, FL, USA; VCC MC8 Physiologic Instruments, Reno, NV, USA) connected to the apical and basolateral chambers via Ag/AgCl electrodes and agar bridges (1 M KCl in 1% agar). Before each experiment, the offset between voltage electrodes and the fluid resistance were adjusted to compensate parameters. The short-circuit current was recorded on a personal computer after analogical to digital conversion.

## Figures and Tables

**Table 1:** Chemical structure and potency trend of the new hybrids **12b, 13b, 14b, 17,** and **18** as F508del CFTR correctors.

Compound	Structure	EC_50_ (µM)
**12b**	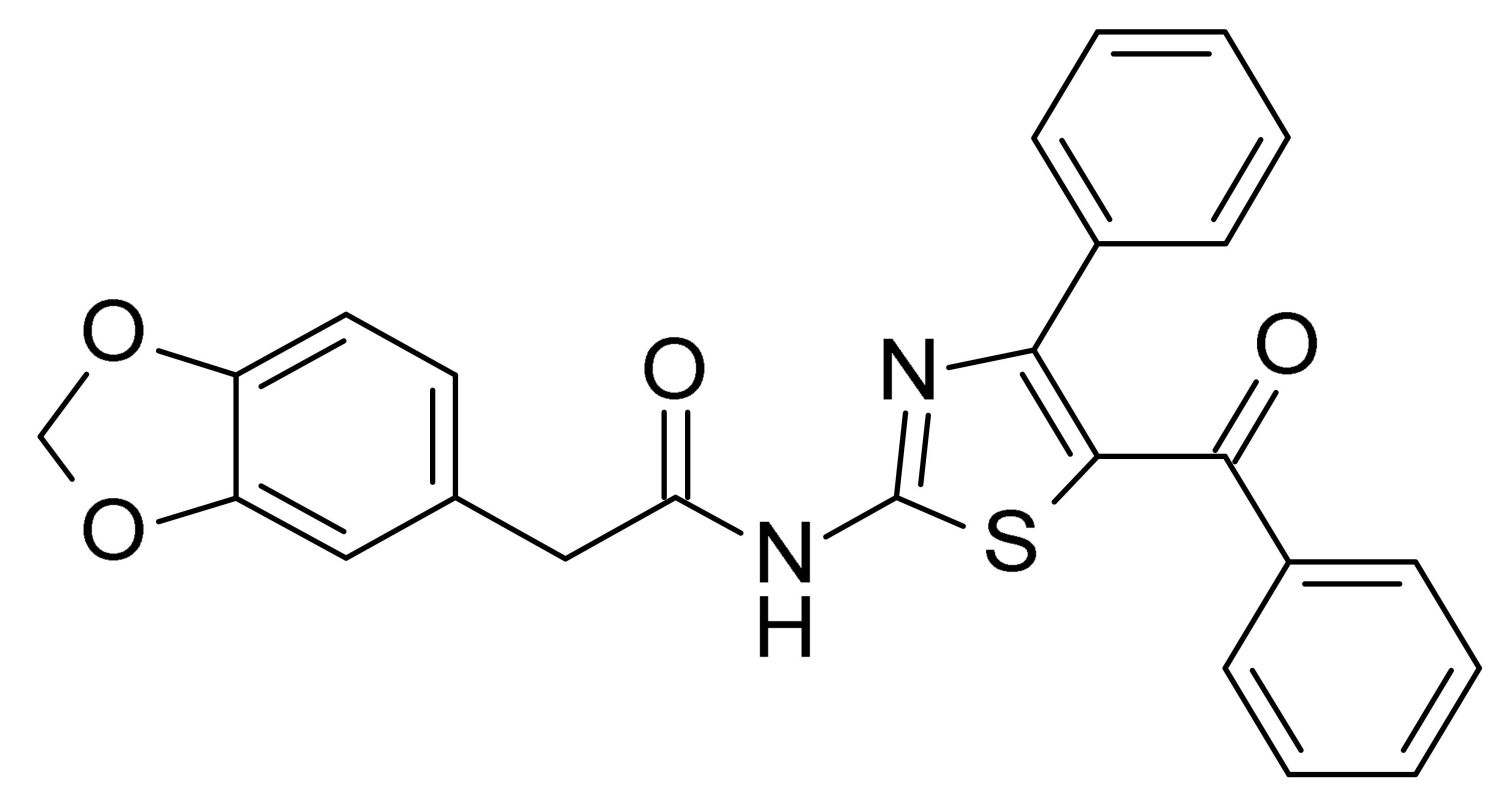	NA
**13b**	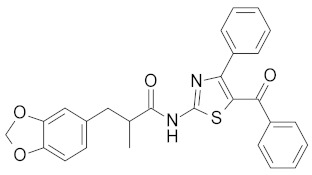	NA
**14b**	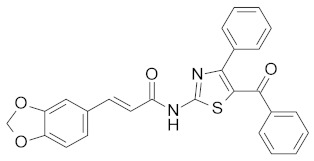	NA
**17**	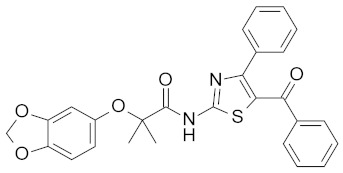	NA
**18**	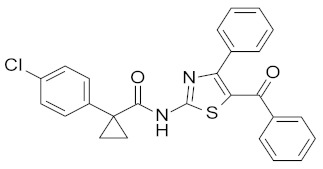	NA

NA = not active.

**Table 2:**
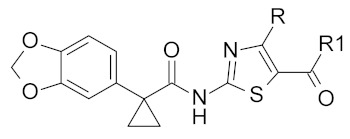
Chemical structure and potency trends of the newly described hybrids **9a–9z** as F508del CFTR correctors.

Compound	R	R1	EC_50_ (µM)
**9a**	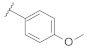	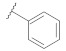	0.35
**9b**	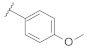	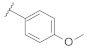	0.064
**9c**	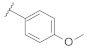	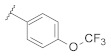	0.14
**9d**	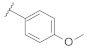	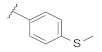	0.10
**9e**	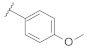	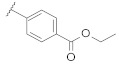	0.56
**9f**	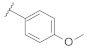	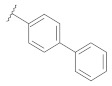	0.14
**9g**	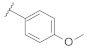	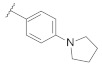	0.033
**9h**	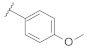	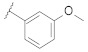	0.12
**9i**	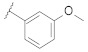	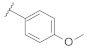	0.19
**9j**	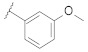	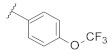	0.037
**9k**	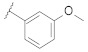	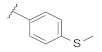	0.36
**9l**	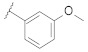	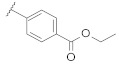	0.49
**9m**	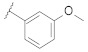	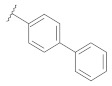	0.74
**9n**	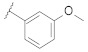	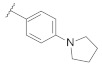	0.6
**9o**	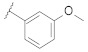	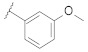	0.21
**9p**	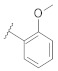	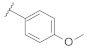	0.22
**9q**	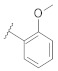	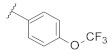	0.15
**9r**	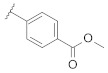	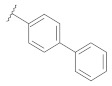	0.12
**9s**	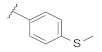	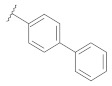	0.13
**9t**	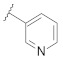	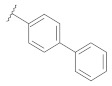	0.13
**9u**	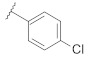	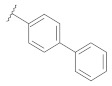	0.64
**9v**	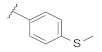	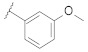	0.28
**9w**	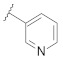	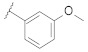	1.52
**9x**	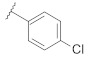	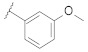	0.37
**9y**	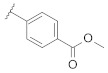	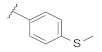	0.076
**9z**	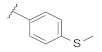	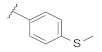	0.30

**Table 3:** List of the recurrent pharmacophore groups shared by 80% of the F508del-CFTR correctors herein studied, in tandem with their ID, score, radius, and expression parameters.

ID	Score	Radius (Å)	Expression
F1	100%	1.39	Don
F2	100%	2.21	Aro|Hyd|Acc
F3	100%	2.35	Aro|PiN|Hyd|Acc
F4	94%	1.36	Acc
F5	88%	1.20	PiN
F6	84%	2.22	PiN|Acc
F7	81	1.19	Aro|Hyd|
F8	81	1.19	Don2
F9	81	1.53	Aro|Hyd|

**Table 4:** Calculated ADME properties as representative of the absorption and distribution features for a number of hybrid derivatives and for the drug-like correctors VX-809, VX-661, and VX-445, taken as reference compounds (highlighted in grey). Hybrids discovered within the first [27] and the second series [28] are depicted in cyan and light orange, respectively. Newly discovered analogues, reported within the third series of hybrids, are reported in green.

Compound	cLogP ^a^	MW ^b^	HBA ^c^	HBD ^d^	nRot_bond ^e^	HIA ^f^ (%)	Vd ^g^(l/kg) ^b^	%PPB ^h^	LogKaHSA ^i,d^	%F ^l^ (oral) ^e^
**VX-809**	3.97	452.41	7	2	5	100	0.31	98	4.26	99.6
**VX-661**	2.55	520.5	8	4	8	100	2.3	93	3.38	97.2
**VX-445**	3.91	597.65	11	1	9	100	0.26	99	5.25	99.1
**2a**	3.85	468.52	6	1	6	100	3.1	100	5.01	56
**2b**	4.25	515.38	7	1	7	100	3.1	97	4.16	69.7
**2c**	3.87	470.93	7	1	7	100	3.8	98	4.32	71.8
**2d**	3.87	470.93	7	1	7	100	2.9	98	4.32	71.8
**3b**	4.01	457.34	5	1	4	100	3	97	4.18	78
**3e**	3.03	396.44	5	1	4	100	2.8	95	4.09	92.4
**4d**	3.46	490.32	5	1	4	100	2.9	96	4.29	79.7
**5b**	2.59	380.42	6	2	4	100	2.2	95	3.8	99
**5c**	2.62	394.44	6	2	4	100	2.4	95	3.82	98.6
**6a**	4.17	493.32	5	1	4	100	4.3	98	4.21	84.7
**6c**	2.97	416.4	6	2	4	100	2.7	96	3.86	98.7
**7a**	3.96	514.62	6	1	7	100	4.2	100	5	45.2
**7h**	3.86	498.55	7	1	7	100	3.1	100	5.02	50.8
**7j**	4.58	526.6	7	1	9	100	4.5	99	5.06	36.6
**7m**	5.27	544.62	6	1	7	100	5.1	100	5.76	13.5
**7n**	3.83	537.63	7	1	7	100	4.5	100	5.1	68.1
**7q**	3.86	498.55	7	1	7	100	3.2	100	5.02	50.8
**9a**	3.86	498.55	7	1	7	100	3.1	100	5.02	50.8
**9b**	3.53	528.58	8	1	8	100	2.9	99	5.04	61.3
**9d**	4.99	544.64	7	1	8	100	4.1	100	4.99	36.6
**9g**	3.82	567.66	8	1	8	100	4.5	99	5.01	64.6
**9f**	5.76	574.65	7	1	8	100	5	100	5.76	11
**9j**	4.06	582.55	8	1	9	100	4.3	100	5.25	61.4
**9q**	4.06	582.55	8	1	9	100	4.4	100	5.25	61.4
**9s**	5.26	590.71	6	1	8	100	5.4	100	5.74	7
**9t**	4.72	545.61	7	1	7	100	4.3	100	5.49	32
**9u**	5.63	579.07	6	1	7	100	5.6	100	5.96	4.9
**9v**	4.99	544.64	7	1	8	100	4	100	4.99	36.6
**9x**	4.83	533	7	1	7	100	4.2	100	5.28	25.8
**9y**	4	572.65	8	1	9	100	4.1	100	5.07	44.9

^a^ cLogP represents the logarithmic ratio of the octanol–water partitioning coefficient, ^b^ molecular weight (MW) of compounds, ^c^ number of H-bonding acceptor, ^d^ number of donor groups, ^e^ number of rotatable bonds, ^f^ HIA stands for the human intestinal absorption, reported as percentage of the compound able to pass through the intestinal membrane; ^g^ prediction of Volume of Distribution (Vd) of the molecule in the body; ^h^ plasmatic protein binding; ^i^ ligand affinity toward human serum albumin; ^l^ oral bioavailability as a percentage.

## Data Availability

All data generated or analyzed during this study are included in this manuscript and the Supplementary Material.

## Supplementary Materials

The following supporting information can be downloaded at: https://www.mdpi.com/article/10.3390/ph15030274/s1, Figure S1: Docking positioning of the two reference compounds ALK-809 and SUL-809 at the modelled F508del-CFTR., Figure S2: Docking positioning of ALK-809 and of the hybrid **2b** at the modelled F508del-CFTR., Figure S3: Docking positioning of ALK-809 and of the hybrid **2a** at the modelled F508del-CFTR., Figure S4: Docking positioning of the hybrid **7m** at the modelled F508del-CFTR., Figure S5: Docking positioning of the hybrid precursor **7a** and of the newly synthesized analogues **9d** and **9y** at the modelled F508del-CFTR., Figure S6: Docking positioning of the newly synthesized compounds **9g** and **9j** at the modelled F508del-CFTR., Figure S7: Pharmacophore model as developed based on the most potent hybrids., Figure S8: Comparison of the developed pharmacophore model with the chemical structure of VX-809 and VX-661. Table S1: Chemical structure and biological activity of the first series of VX-809 and amino aryl-thiazole hybrids, Table S2: Chemical structure and biological activity of the second series of developed hybrids, Table S3: Binding affinity values obtained by molecular docking studies., Table S4: Chemical structure (as SMILE format) and biological activity of the most potent hybrids so far developed., Table S5: Molecular structure and biological activity as F508del-CFTR correctors of the most potent hybrids discovered, featuring pEC_50_ values >6.50 M. Experimental procedure: synthesis and characterization for all compounds.
