# Supplementary material for: Journey on VX-809-Based Hybrid Derivatives towards Drug-like F508del-CFTR Correctors: From Molecular Modeling to Chemical Synthesis and Biological Assays
Source: Pharmaceuticals (Basel). 2022 Feb 23;15(3):274. doi: 10.3390/ph15030274 (PMC8955485; doi:10.3390/ph15030274)
Supplement: Supplementary file 1 [file pharmaceuticals-15-00274-s001.zip › Parodi A.Supporting Information2022rev.pdf]

pharmaceuticals

Supporting Information

Figure S1. Docking positioning of the two reference compounds ALK-809 and SUL-809 at the modelled F508del-CFTR.

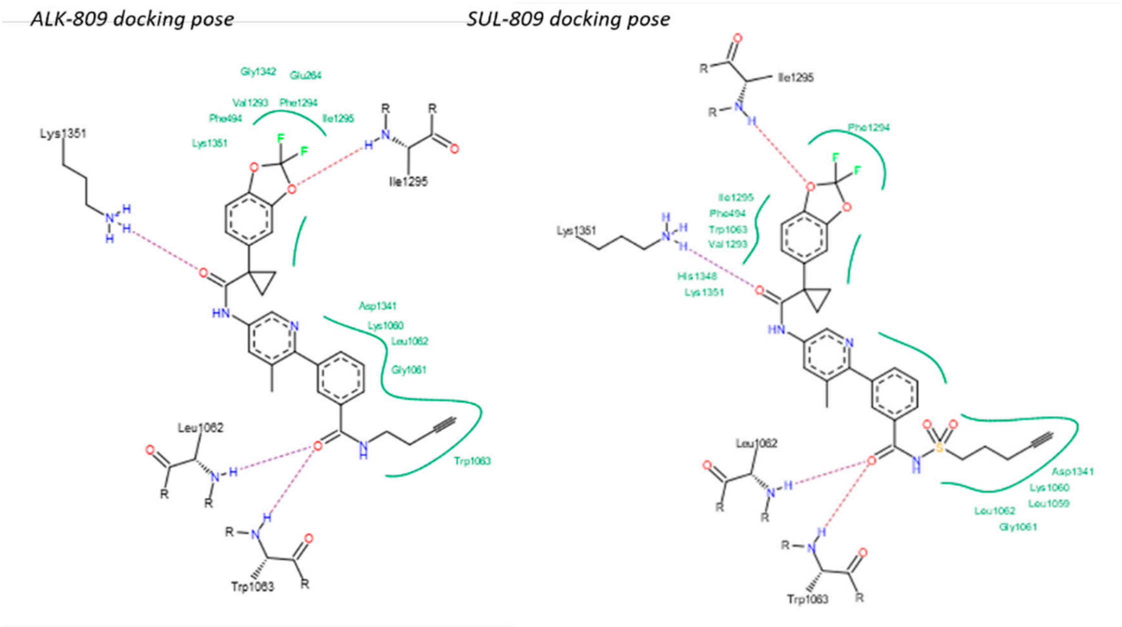

Figure S2. Docking positioning at the modelled F508del-CFTR of ALK-809 (C atom; pink) and **2b** (C atom; green), as representative of the hybrids first series. The related ligplot images are shown on the right.

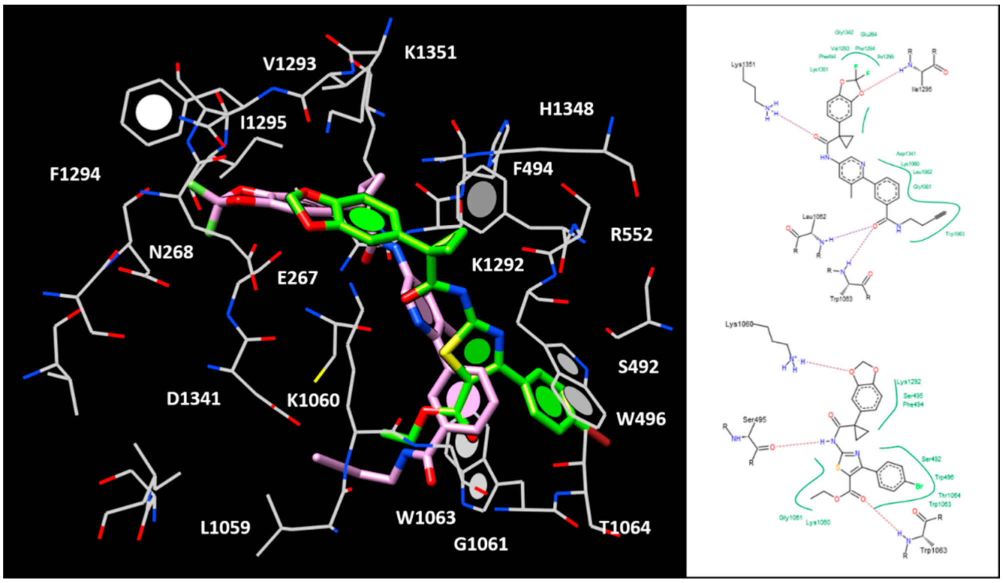

Table S1. Chemical structure and biological activity of the first series of VX-809 and amino aryl-thiazole hybrids [27].

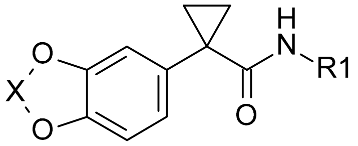

compound	X	R1	EC_{50} (μM)
**2a**	$-CH_2$	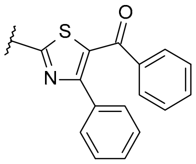	0.087
**2b**	$-CH_2$	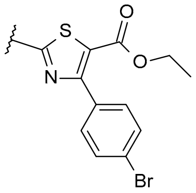	0.3
**2c**	$-CH_2$	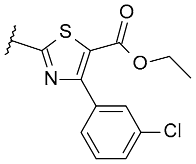	0.55
**2d**	$-CH_2$	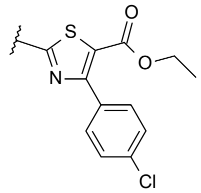	0.9
**3a**	$-CH_2$	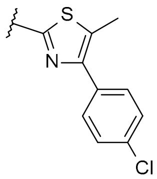	2.9
**3b**	$-CH_2$	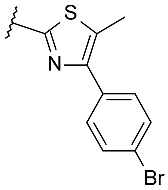	1.9
**3c**	$-CH_2$	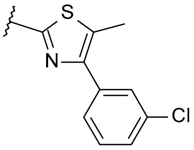	2.3

**3d**	$-CH_2$	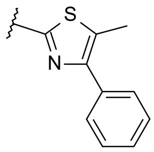	1.6
**3e**	$-CH_2$	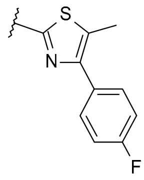	0.9
**4a**	$-CH_2$	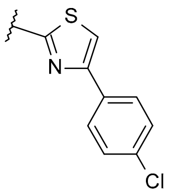	2.9
**4b**	$-CH_2$	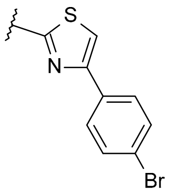	4.9
**4c**	$-CH_2$	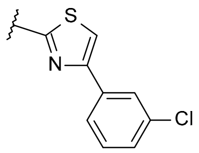	8.2
**4d**	$-CH_2$	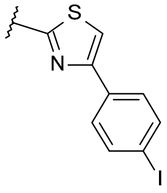	1.2
**4e**	$-CH_2$	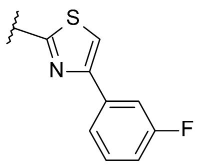	2.1
**5a**	$-CH_2$	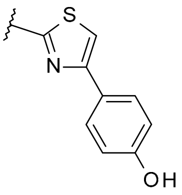	1.8

**5b**	-CH_2	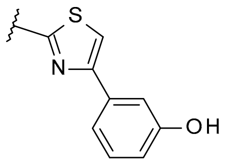	2.3
**5c**	-CH_2	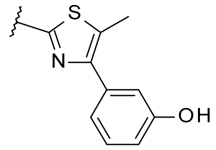	1.2
**5d**	-CH_2	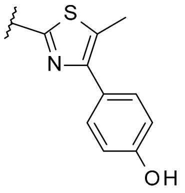	3.9
**5e**	-CH_2	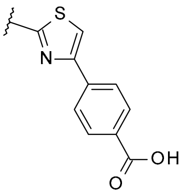	> 5
**5f**	-CH_2	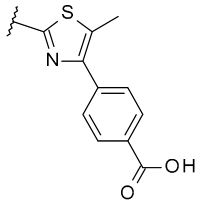	2.9
**5g**	-CH_2	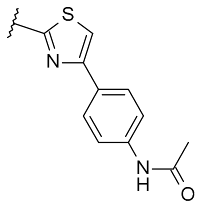	> 5
**6a**	-CF_2	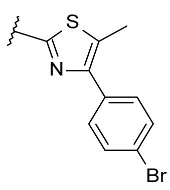	3.8
**6b**	-CF_2	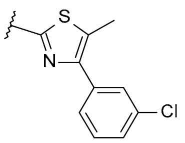	5.4

**6c**	$-CF_2$	1.3
**6d**	$-CF_2$	1.7
**6e**	$-CF_2$	4

Figure S3. Docking positioning at the modelled F508del-CFTR of ALK-809 (C atom; pink) and **2a** (C atom; yellow). The hybrid **2a** ligplot is shown on the right.

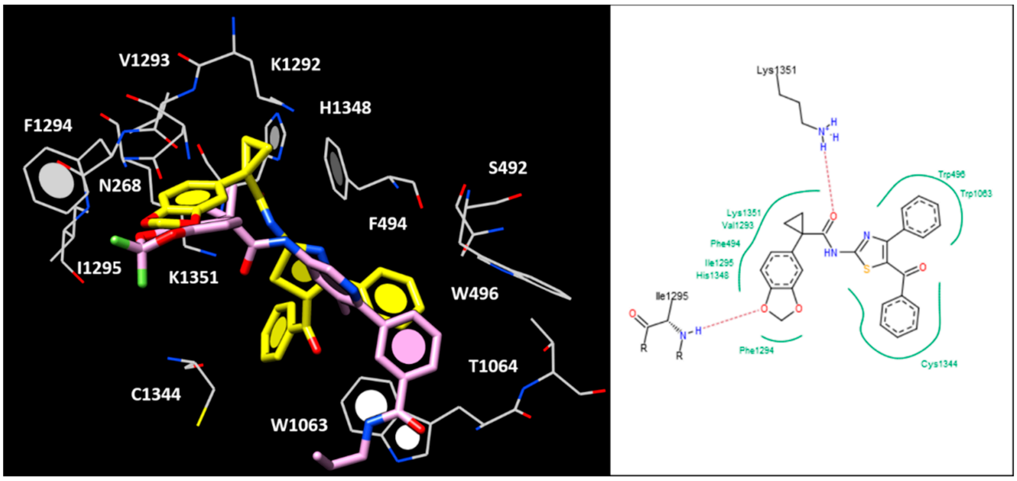

Table S2. Chemical structure and biological activity of most potent hybrids second series [28].

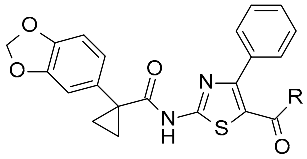

compound	R	EC_{50}(μM)

**7a**	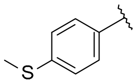	0.10
**7b**	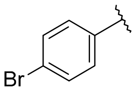	0.53
**7c**	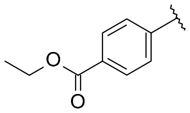	0.41
**7d**	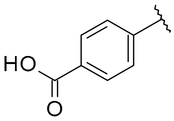	7.31
**7e**	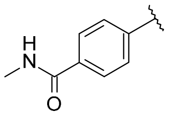	not active
**7f**	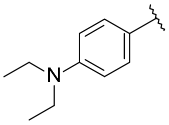	0.21
**7g**	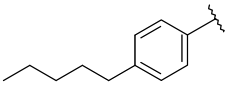	0.43
**7h**	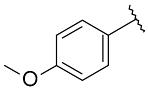	0.45
**7i**	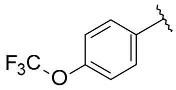	0.36
**7j**	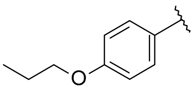	0.017
**7k**	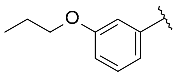	0.16
**7l**	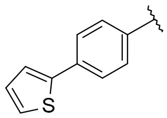	1.06

**7m**	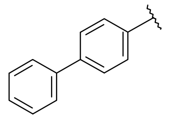	0.07
**7n**	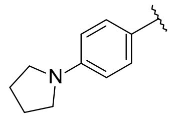	0.37
**7o**	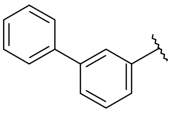	0.25
**7p**	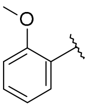	0.14
**7q**	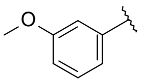	0.10
**7r**	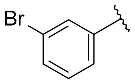	0.18
**7s**	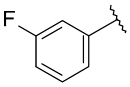	0.13
**7t**	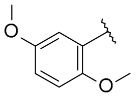	0.11
**7u**	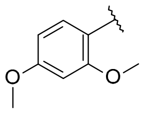	0.40
**7v**	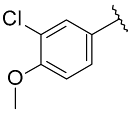	0.41
**7w**	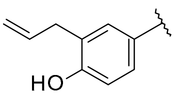	0.28

**7x**	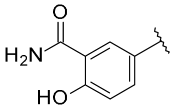	13.70
**7y**	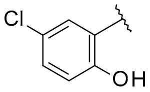	1.60
**7z**	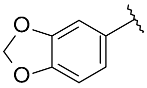	0.11

Table S3. Binding affinity values obtained by molecular docking studies of the previously synthesized compounds **7a**, **7m**, **7q** (highlighted in light orange), taken as reference compounds of the hybrids first series, and of the newly developed **9b**, **9d**, **9g**, **9h**, **9i**, **9j**, **9q**, **9y** (second series) (highlighted in green).

Protein-Ligand Complex (LeadIT)	Binding Affinity Energy G (kJ/mol)	Protein-Ligand Complex (LeadIT)	Binding Affinity Energy G (kJ/mol)
F508del CFTR-**7a**	-16.0	F508del CFTR-**9h**	-22.0
F508del CFTR-**7m**	-19.0	F508del CFTR-**9i**	-15.0
F508del CFTR-**7q**	-15.0	F508del CFTR-**9j**	-19.0
F508del CFTR-**9b**	-17.0	F508del CFTR-**9q**	-15.0
F508del CFTR-**9d**	-14.0	F508del CFTR-**9y**	-20.0
F508del CFTR-**9g**	-16.0		

Figure S4. Docking positioning of the hybrid **7m** at the modelled F508del-CFTR.

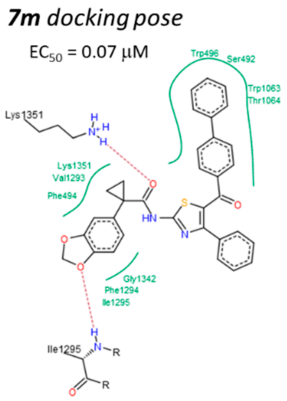

Figure S5. Docking positioning of the hybrid precursor **7a** and of the newly synthesized analogues **9d** and **9y** at the modelled F508del-CFTR.

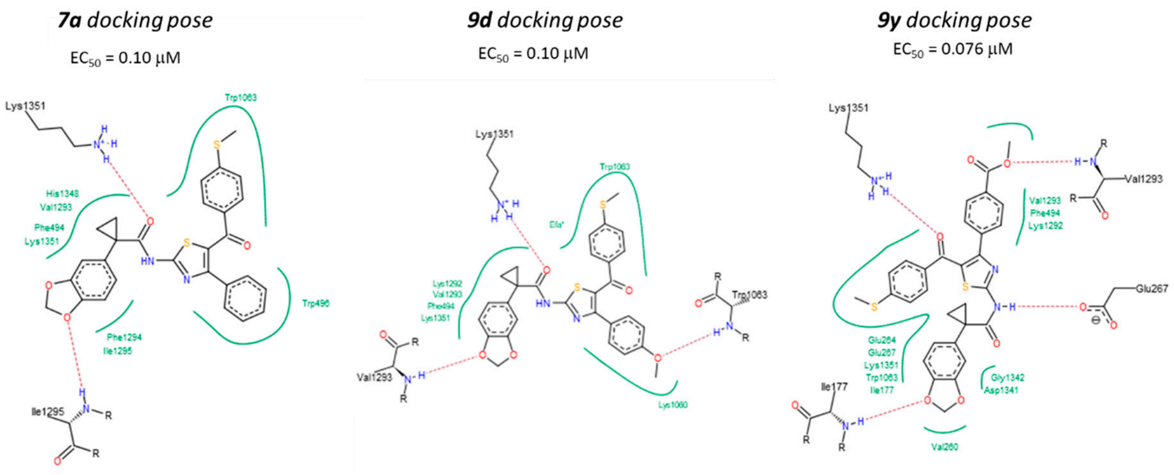

Figure S6. Docking positioning of the newly synthesized compounds **9g** (C atom; purple) and **9j** (C atom; white) at the modelled F508del-CFTR.

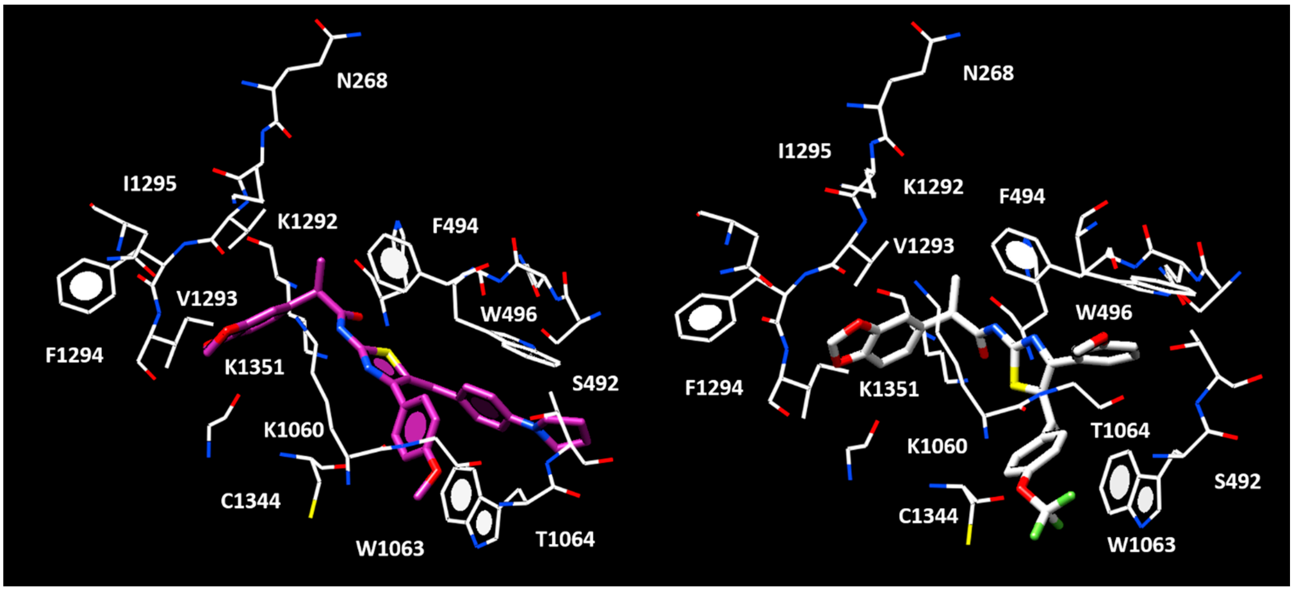

Table S4. Chemical structure (as SMILE format) and biological activity of the most potent F508del-CFTR correctors discovered within the first (highlighted in cyan) [27] and second series of hybrids (highlighted in cyan) [28]. The newly discovered third series of compounds are also reported (highlighted in green).

Hybrids	Molecules as SMILE format	EC_{50} (µM)	pEC_{50} (M)
2a	s1c(C(=O)c2ccccc2)c(nc1NC(=O)C1(CC1)c1cc2OCOc2cc1)-c1ccccc1	0.087	7.06

**2b**	Brc1ccc(cc1)-c1nc(sc1C(OCC)=O)NC(=O)C1(CC1)c1cc2OCOc2cc1	0.300	6.52
**2c**	Clc1cc(ccc1)-c1nc(sc1C(OCC)=O)NC(=O)C1(CC1)c1cc2OCOc2cc1	0.550	6.26
**2d**	Clc1ccc(cc1)-c1nc(sc1C(OCC)=O)NC(=O)C1(CC1)c1cc2OCOc2cc1	0.900	6.05
**3a**	Clc1ccc(cc1)-c1nc(sc1C)NC(=O)C1(CC1)c1cc2OCOc2cc1	2.900	5.54
**3b**	Brc1ccc(cc1)-c1nc(sc1C)NC(=O)C1(CC1)c1cc2OCOc2cc1	1.900	5.72
**3c**	Clc1cc(ccc1)-c1nc(sc1C)NC(=O)C1(CC1)c1cc2OCOc2cc1	2.300	5.64
**3d**	s1c(C)c(nc1NC(=O)C1(CC1)c1cc2OCOc2cc1)-c1ccccc1	1.600	5.80
**3e**	s1c(C)c(nc1NC(=O)C1(CC1)c1cc2OCOc2cc1)-c1ccc(F)cc1	0.900	6.05
**4a**	Clc1ccc(cc1)-c1nc(sc1)NC(=O)C1(CC1)c1cc2OCOc2cc1	2.900	5.54
**4b**	Brc1ccc(cc1)-c1nc(sc1)NC(=O)C1(CC1)c1cc2OCOc2cc1	4.900	5.31
**4c**	Clc1cc(ccc1)-c1nc(sc1)NC(=O)C1(CC1)c1cc2OCOc2cc1	8.200	5.09
**4d**	Ic1ccc(cc1)-c1nc(sc1)NC(=O)C1(CC1)c1cc2OCOc2cc1	1.200	5.92
**4e**	s1cc(nc1NC(=O)C1(CC1)c1cc2OCOc2cc1)-c1cc(F)ccc1	2.100	5.68
**5a**	s1cc(nc1NC(=O)C1(CC1)c1cc2OCOc2cc1)-c1ccc(O)cc1	1.800	5.74
**5b**	s1cc(nc1NC(=O)C1(CC1)c1cc2OCOc2cc1)-c1cc(O)ccc1	2.300	5.64
**5c**	s1c(C)c(nc1NC(=O)C1(CC1)c1cc2OCOc2cc1)-c1cc(O)ccc1	1.200	5.92
**5d**	s1c(C)c(nc1NC(=O)C1(CC1)c1cc2OCOc2cc1)-c1ccc(O)cc1	3.900	5.41
**5e**	s1cc(nc1NC(=O)C1(CC1)c1cc2OCOc2cc1)-c1ccc(cc1)C(O)=O	5.000	5.30
**5f**	s1c(C)c(nc1NC(=O)C1(CC1)c1cc2OCOc2cc1)-c1ccc(cc1)C(O)=O	2.900	5.54
**5g**	s1cc(nc1NC(=O)C1(CC1)c1cc2OCOc2cc1)-c1ccc(NC(=O)C)cc1	5.000	5.30
**6a**	Brc1ccc(cc1)-c1nc(sc1C)NC(=O)C1(CC1)c1cc2OC(F)(F)Oc2cc1	3.800	5.42
**6b**	Clc1cc(ccc1)-c1nc(sc1C)NC(=O)C1(CC1)c1cc2OC(F)(F)Oc2cc1	5.400	5.27
**6c**	s1cc(nc1NC(=O)C1(CC1)c1cc2OC(F)(F)Oc2cc1)-c1cc(O)ccc1	1.300	5.89
**6d**	s1cc(nc1NC(=O)C1(CC1)c1cc2OC(F)(F)Oc2cc1)-c1ccc(O)cc1	1.700	5.77
**6e**	Clc1cc(ccc1)-c1nc(sc1)NC(=O)C1(CC1)c1cc2OC(F)(F)Oc2cc1	4.000	5.40
**7a**	s1c(C(=O)c2ccc(SC)cc2)c(nc1NC(=O)C1(CC1)c1cc2OCOc2cc1)-c1ccccc1	0.100	7.00
**7b**	Brc1ccc(cc1)C(=O)c1sc(nc1-c1ccccc1)NC(=O)C1(CC1)c1cc2OCOc2cc1	0.530	6.28
**7c**	s1c(C(=O)c2ccc(cc2)C(OCC)=O)c(nc1NC(=O)C1(CC1)c1cc2OCOc2cc1)-c1ccccc1	0.410	6.39
**7d**	s1c(C(=O)c2ccc(cc2)C(O)=O)c(nc1NC(=O)C1(CC1)c1cc2OCOc2cc1)-c1ccccc1	7.310	5.14
**7e**	s1c(C(=O)c2ccc(cc2)C(=O)NC)c(nc1NC(=O)C1(CC1)c1cc2OCOc2cc1)-c1ccccc1	1000.000	3.00
**7f**	s1c(C(=O)c2ccc(N(CC)CC)cc2)c(nc1NC(=O)C1(CC1)c1cc2OCOc2cc1)-c1ccccc1	0.210	6.68
**7g**	s1c(C(=O)c2ccc(cc2)CCCCC)c(nc1NC(=O)C1(CC1)c1cc2OCOc2cc1)-c1ccccc1	0.430	6.37
**7h**	s1c(C(=O)c2ccc(OC)cc2)c(nc1NC(=O)C1(CC1)c1cc2OCOc2cc1)-c1ccccc1	0.450	6.35
**7i**	s1c(C(=O)c2ccc(OC(F)(F)F)cc2)c(nc1NC(=O)C1(CC1)c1cc2OCOc2cc1)-c1ccccc1	0.360	6.44
**7j**	s1c(C(=O)c2ccc(OCCC)cc2)c(nc1NC(=O)C1(CC1)c1cc2OCOc2cc1)-c1ccccc1	0.017	7.77
**7k**	s1c(C(=O)c2cc(OCCC)ccc2)c(nc1NC(=O)C1(CC1)c1cc2OCOc2cc1)-c1ccccc1	0.160	6.80
**7l**	s1c(C(=O)c2ccc(cc2)-c2sccc2)c(nc1NC(=O)C1(CC1)c1cc2OCOc2cc1)-c1ccccc1	1.060	5.97
**7m**	s1c(C(=O)c2ccc(cc2)-c2ccccc2)c(nc1NC(=O)C1(CC1)c1cc2OCOc2cc1)-c1ccccc1	0.070	7.15
**7n**	s1c(C(=O)c2ccc(N3CCCC3)cc2)c(nc1NC(=O)C1(CC1)c1cc2OCOc2cc1)-c1ccccc1	0.370	6.43
**7o**	s1c(C(=O)c2cc(ccc2)-c2ccccc2)c(nc1NC(=O)C1(CC1)c1cc2OCOc2cc1)-c1ccccc1	0.250	6.60
**7p**	s1c(C(=O)c2ccccc2OC)c(nc1NC(=O)C1(CC1)c1cc2OCOc2cc1)-c1ccccc1	0.140	6.85

**7q**	s1c(C(=O)c2cc(OC)ccc2)c(nc1NC(=O)C1(CC1)c1cc2OCOc2cc1)-c1ccccc1	0.100	7.00
**7r**	Brc1cc(ccc1)C(=O)c1sc(nc1-c1ccccc1)NC(=O)C1(CC1)c1cc2OCOc2cc1	0.180	6.74
**7s**	s1c(C(=O)c2cc(F)ccc2)c(nc1NC(=O)C1(CC1)c1cc2OCOc2cc1)-c1ccccc1	0.130	6.89
**7t**	s1c(C(=O)c2cc(OC)ccc2OC)c(nc1NC(=O)C1(CC1)c1cc2OCOc2cc1)-c1ccccc1	0.110	6.96
**7u**	s1c(C(=O)c2ccc(OC)cc2OC)c(nc1NC(=O)C1(CC1)c1cc2OCOc2cc1)-c1ccccc1	0.400	6.40
**7v**	Clc1cc(ccc1OC)C(=O)c1sc(nc1-c1ccccc1)NC(=O)C1(CC1)c1cc2OCOc2cc1	0.410	6.39
**7w**	s1c(C(=O)c2cc(CC=C)c(O)cc2)c(nc1NC(=O)C1(CC1)c1cc2OCOc2cc1)-c1ccccc1	0.280	6.55
**7x**	s1c(C(=O)c2cc(C(=O)N)c(O)cc2)c(nc1NC(=O)C1(CC1)c1cc2OCOc2cc1)-c1ccccc1	13.700	4.86
**7y**	Clc1cc(C(=O)c2sc(nc2-c2ccccc2)NC(=O)C2(CC2)c2cc3OCOc3cc2)c(O)cc1	1.600	5.80
**7z**	s1c(C(=O)c2cc3OCOc3cc2)c(nc1NC(=O)C1(CC1)c1cc2OCOc2cc1)-c1ccccc1	0.110	6.96
**24a**	s1c(C(=O)C)c(nc1NC(=O)C1(CC1)c1cc2OCOc2cc1)-c1ccccc1	2.720	5.57
**24b**	s1c(C(=O)c2cccnc2)c(nc1NC(=O)C1(CC1)c1cc2OCOc2cc1)-c1ccccc1	0.790	6.10
**24c**	s1c(C(=O)N2CCOCC2)c(nc1NC(=O)C1(CC1)c1cc2OCOc2cc1)-c1ccccc1	8.500	5.07
**9a**	s1c(C(=O)c2ccccc2)c(nc1NC(=O)C1(CC1)c1cc2OCOc2cc1)-c1ccc(OC)cc1	0.350	6.46
**9b**	s1c(C(=O)c2ccc(OC)cc2)c(nc1NC(=O)C1(CC1)c1cc2OCOc2cc1)-c1ccc(OC)cc1	0.064	7.19
**9c**	s1c(C(=O)c2ccc(OC(F)(F)F)cc2)c(nc1NC(=O)C1(CC1)c1cc2OCOc2cc1)-c1ccc(OC)cc1	0.140	6.85
**9d**	s1c(C(=O)c2ccc(SC)cc2)c(nc1NC(=O)C1(CC1)c1cc2OCOc2cc1)-c1ccc(OC)cc1	0.100	7.00
**9e**	s1c(C(=O)c2ccc(cc2)C(OCC)=O)c(nc1NC(=O)C1(CC1)c1cc2OCOc2cc1)-c1ccc(OC)cc1	0.560	6.25
**9f**	s1c(C(=O)c2ccc(cc2)-c2ccccc2)c(nc1NC(=O)C1(CC1)c1cc2OCOc2cc1)-c1ccc(OC)cc1	0.140	6.85
**9g**	s1c(C(=O)c2ccc(N3CCCC3)cc2)c(nc1NC(=O)C1(CC1)c1cc2OCOc2cc1)-c1ccc(OC)cc1	0.033	7.48
**9h**	s1c(C(=O)c2cc(OC)ccc2)c(nc1NC(=O)C1(CC1)c1cc2OCOc2cc1)-c1ccc(OC)cc1	0.120	6.92
**9i**	s1c(C(=O)c2ccc(OC)cc2)c(nc1NC(=O)C1(CC1)c1cc2OCOc2cc1)-c1cc(OC)ccc1	0.190	6.72
**9j**	s1c(C(=O)c2ccc(OC(F)(F)F)cc2)c(nc1NC(=O)C1(CC1)c1cc2OCOc2cc1)-c1cc(OC)ccc1	0.037	7.43
**9k**	s1c(C(=O)c2ccc(SC)cc2)c(nc1NC(=O)C1(CC1)c1cc2OCOc2cc1)-c1cc(OC)ccc1	0.360	6.44
**9l**	s1c(C(=O)c2ccc(cc2)C(OCC)=O)c(nc1NC(=O)C1(CC1)c1cc2OCOc2cc1)-c1cc(OC)ccc1	0.490	6.31
**9m**	s1c(C(=O)c2ccc(cc2)-c2ccccc2)c(nc1NC(=O)C1(CC1)c1cc2OCOc2cc1)-c1cc(OC)ccc1	0.740	6.13
**9n**	s1c(C(=O)c2ccc(N3CCCC3)cc2)c(nc1NC(=O)C1(CC1)c1cc2OCOc2cc1)-c1cc(OC)ccc1	0.600	6.22
**9o**	s1c(C(=O)c2cc(OC)ccc2)c(nc1NC(=O)C1(CC1)c1cc2OCOc2cc1)-c1cc(OC)ccc1	0.210	6.68
**9p**	s1c(C(=O)c2ccc(OC)cc2)c(nc1NC(=O)C1(CC1)c1cc2OCOc2cc1)-c1ccccc1OC	0.220	6.66
**9q**	s1c(C(=O)c2ccc(OC(F)(F)F)cc2)c(nc1NC(=O)C1(CC1)c1cc2OCOc2cc1)-c1ccccc1OC	0.150	6.82
**9r**	s1c(C(=O)c2ccc(cc2)-c2ccccc2)c(nc1NC(=O)C1(CC1)c1cc2OCOc2cc1)-c1ccc(cc1)C(OC)=O	0.120	6.92
**9s**	s1c(C(=O)c2ccc(cc2)-c2ccccc2)c(nc1NC(=O)C1(CC1)c1cc2OCOc2cc1)-c1ccc(SC)cc1	0.130	6.89
**9t**	s1c(C(=O)c2ccc(cc2)-c2ccccc2)c(nc1NC(=O)C1(CC1)c1cc2OCOc2cc1)-c1cccnc1	0.130	6.89
**9u**	Clc1ccc(cc1)-c1nc(sc1C(=O)c1ccc(cc1)-c1ccccc1)NC(=O)C1(CC1)c1cc2OCOc2cc1	0.640	6.19

**9v**	s1c(C(=O)c2cc(OC)ccc2)c(nc1NC(=O)C1(CC1)c1cc2OCOc2cc1)-c1ccc(SC)cc1	0.280	6.55
**9w**	s1c(C(=O)c2cc(OC)ccc2)c(nc1NC(=O)C1(CC1)c1cc2OCOc2cc1)-c1cccnc1	1.520	5.82
**9x**	Clc1ccc(cc1)-c1nc(sc1C(=O)c1cc(OC)ccc1)NC(=O)C1(CC1)c1cc2OCOc2cc1	0.370	6.43
**9y**	s1c(C(=O)c2ccc(SC)cc2)c(nc1NC(=O)C1(CC1)c1cc2OCOc2cc1)-c1ccc(cc1)C(OC)=O	0.076	7.12
**9z**	s1c(C(=O)c2cc(SC)ccc2)c(nc1NC(=O)C1(CC1)c1cc2OCOc2cc1)-c1ccc(SC)cc1	0.300	6.52

Table S5. Molecular structure and biological activity as F508del-CFTR correctors of the most potent hybrids discovered, featuring pEC_{50} values > 6.50 M. Compounds belonging to the first [27], second [28] and to the newly third series of hybrids are highlighted in cyan, light orange and green, respectively. The corresponding ranking number is reported within brackets.

**Hybrid**	**Molecular Structure**	**pEC_{50} (M)**	**Hybrid**	**Molecular Structure**	**pEC_{50} (M)**
**(1) 7j**	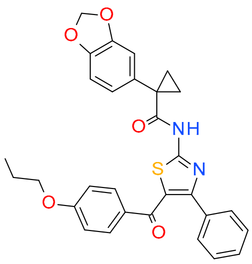	7.77	**(17) 9t**	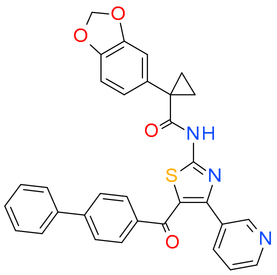	6.89
**(2) 9g**	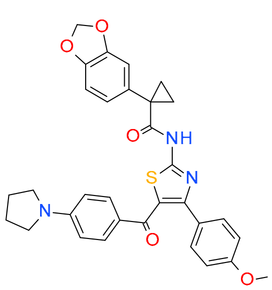	7.48	**(18) 7p**	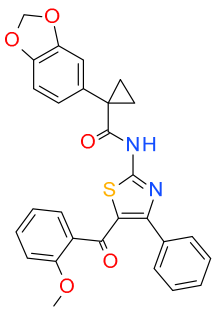	6.85
**(3) 9j**	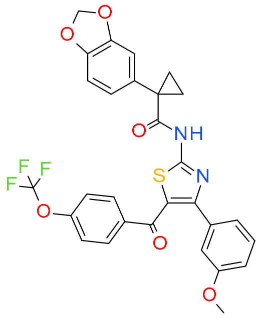	7.43	**(19) 9c**	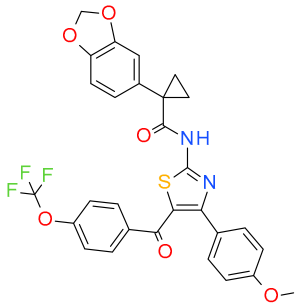	6.85

**(4) 9b**	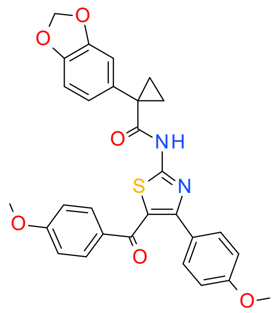	7.19	**(20) 9f**	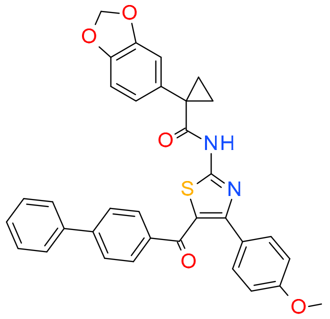	6.85
**(5) 7m**	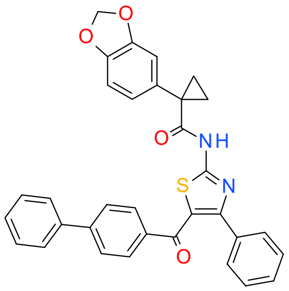	7.15	**(21) 9q**	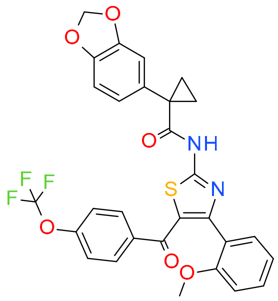	6.82
**(6) 9y**	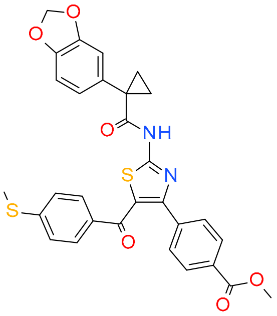	7.12	**(22) 7k**	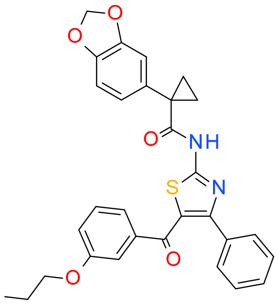	6.80
**(7) 2a**	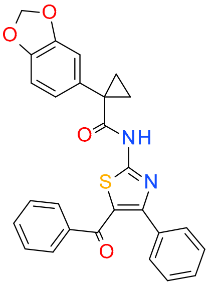	7.06	**(23) 7r**	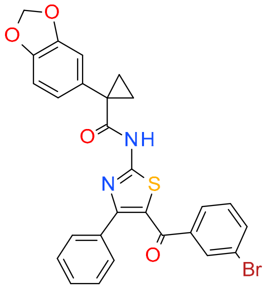	6.74
**(8) 7a**	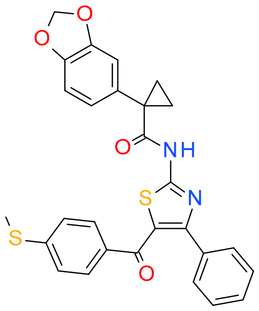	7.00	**(24) 9i**	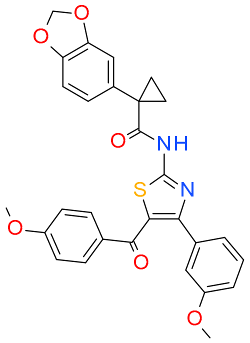	6.72

**(9) 7q**	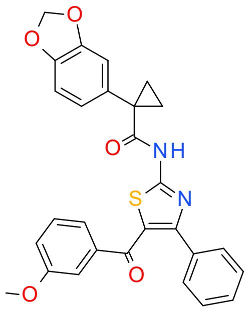	7.00	**(25) 7f**	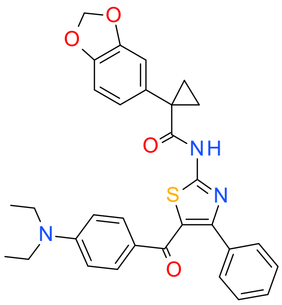	6.68
**(10) 9d**	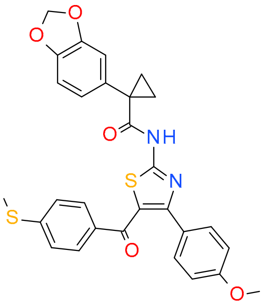	7.00	**(26) 9o**	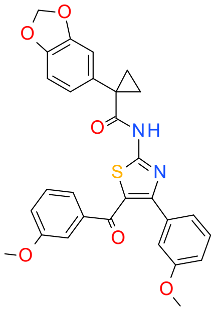	6.68
**(11) 7t**	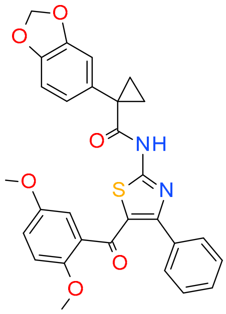	6.96	**(27) 9p**	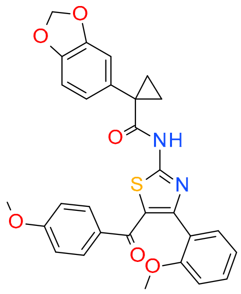	6.66
**(12) 7z**	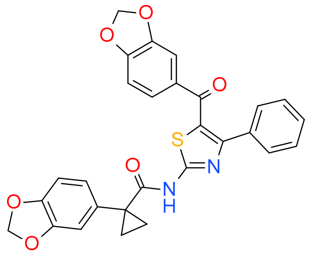	6.96	**(28) 7o**	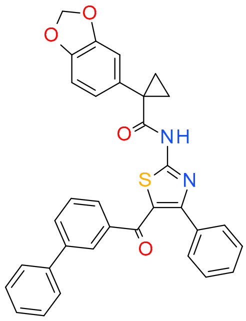	6.60
**(13) 9h**	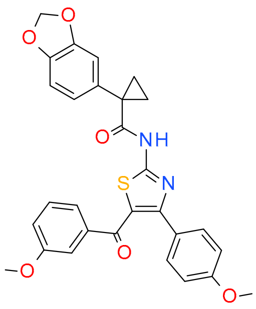	6.92	**(29) 7w**	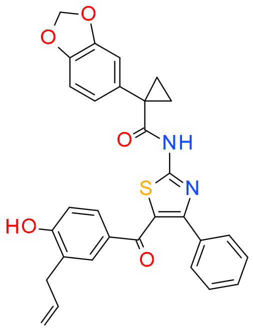	6.55

**(14) 9r**	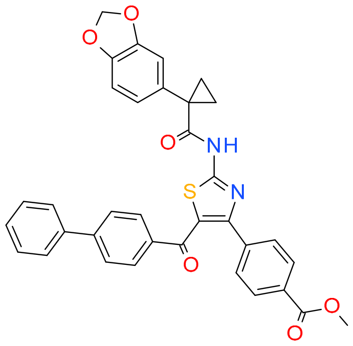	6.92	**(30) 9v**	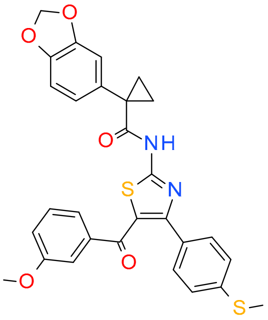	6.55
**(15) 7s**	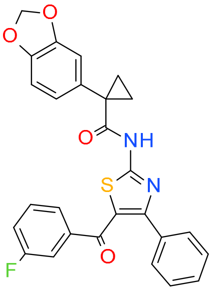	6.89	**(31) 2b**	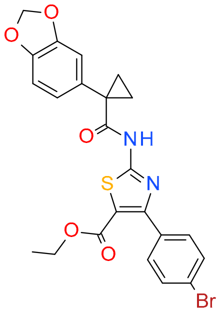	6.52
**(16) 9s**	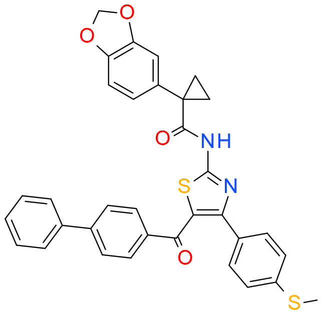	6.89	**(32) 9z**	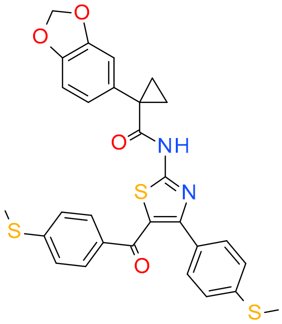	6.52

Figure S7. Pharmacophore model of the most potent hybrids shown in the previous Table S5. Alignment of the thirty-two derivatives is reported, being **9g** (chosen as the most potent hybrid of the new third series) shown in stick (C atom; white).

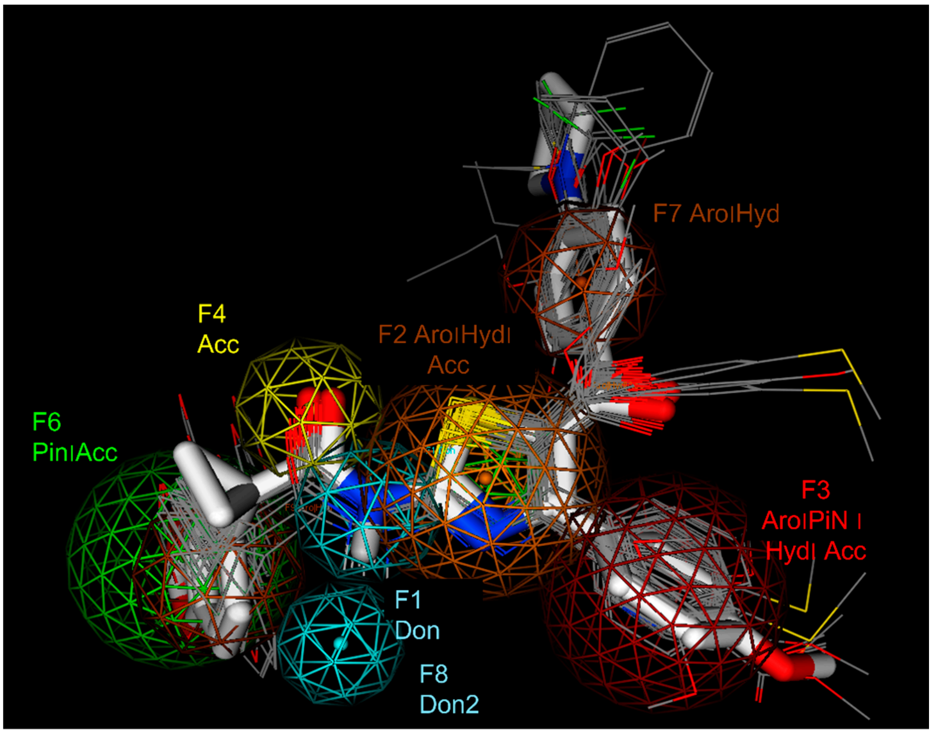

Figure S8. Pharmacophore model of the most potent hybrids shown in the previous Table S5, being **9g** (chosen as the most potent hybrid of the new third series) shown in stick (C atom; white). The superposed structure of VX-809 (C atom; green) and of VX-661 (C atom; yellow) are also reported.

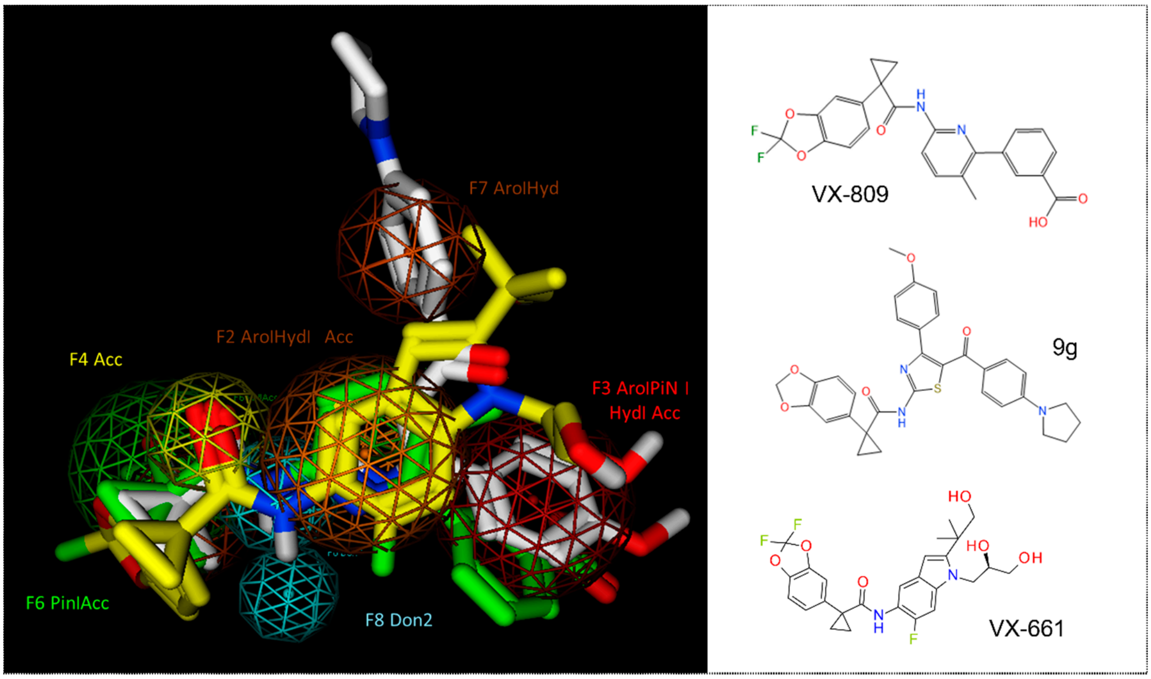

Synthesis and characterization of compounds

*1-(benzo[d][1,3]dioxol-5-yl)-N-(5-(4-methoxybenzoyl)-4-(4-methoxyphenyl)thiazol-2-yl)cyclopropane-1-carboxamide (**9b**)*

Compound **9b** (13.5 mg, 26%) was obtained from (2-amino-4-(4-methoxyphenyl)thiazol-5-yl)(4-methoxyphenyl)methanone (34.0 mg, 0.1 mmol) and benzo[1,3]dioxol-5-yl-cyclopropanecarboxylic acid (20.6 mg, 0.1 mmol) in the same manner as described for **9a**.

^{1}H NMR (200 MHz, DMSO-d6): δ 11.91 (s, 1H, broad, NH); 7.53-6.72 (m, 11H, arom); 6.02 (s, 2H, OCH_2O); 3.62 (s, 3H, OCH_3); 3.57 (s, 3H, OCH_3); 1.64-1.42 (m, 2H, cyclopr); 1.39-1.10 (m, 2H, cyclopr).

^{13}C NMR (50 MHz, DMSO-d6): δ 186.4, 172.2, 159.7, 159.1, 158.3, 153.7, 146.8, 146.3, 138.6, 135.1, 131.7, 128.8, 126.2, 124.5, 123.2, 121.3, 118.3, 113.8, 112.6, 110.2, 107.8, 100.6, 54.7, 30.2, 15.4.

HRMS (ESI) calculated for $C_{29}H_{25}N_2O_6S$:[M + H]$^+$ 529.14332; found 529.14552

*1-(benzo[d][1,3]dioxol-5-yl)-N-(4-(4-methoxyphenyl)-5(4(trifluoromethoxy)benzoyl)thiazol-2-yl)cyclopropane-1-carboxamide (**9c**)*

Compound **9c** (10 mg, 17%) was obtained from (2-amino-4-(4-methoxyphenyl)thiazol-5-yl)(4-(trifluoromethoxy)phenyl)methanone (39.4 mg, 0.1 mmol) and benzo[1,3]dioxol-5-yl-cyclopropanecarboxylic acid (20.6 mg, 0.1 mmol) in the same manner as described for **9a**.

^{1}H NMR (200 MHz, DMSO-d6): δ 12.01 (s, 1H, broad, NH); 7.76-6.82 (m, 11H, arom); 6.03 (s, 2H, OCH_2O); 3.66 (s, 3H, OCH_3); 1.65-1.40 (m, 2H, cyclopr); 1.37-1.08 (m, 2H, cyclopr).

^{13}C NMR (50 MHz, DMSO-d6): δ 188.1, 172.3, 159.2, 153.4, 146.8, 146.3, 144.6, 139.9, 133.5, 131.8, 131.4, 129.2, 128.1, 126.2, 123.9, 123.2, 122.7, 121.1, 118.4, 112.9, 110.2, 107.8, 100.6, 54.6, 30.2, 15.4.

HRMS (ESI) calculated for $C_{29}H_{22}F_3N_2O_6S$: [M + H]$^+$ 583.11506; found 583.11454

*1-(benzo[d][1,3]dioxol-5-yl)-N-(4-(4-methoxyphenyl)-5-(4-(methylthio)benzoyl)thiazol-2-yl)cyclopropane-1-carboxamide (**9d**)*

Compound **9d** (14.7 mg, 27%) was obtained from (2-amino-4-(4-methoxyphenyl)thiazol-5-yl)(4-(methylthio)phenyl)methanone (0.1 mmol, 35.6 mg) and benzo[1,3]dioxol-5-yl-cyclopropanecarboxylic acid (20.6 mg, 0.1 mmol) in the same manner as described for **9a**.

^{1}H NMR (200 MHz, DMSO-d6): δ 11.84 (s, 1H, broad, NH); 7.59-6.69 (m, 11H, arom); 6.02 (s, 2H, OCH_2O); 3.68 (s, 3H, OCH_3); 2.45 (s, 3H, SCH_3); 1.62-1.46 (m, 2H, cyclopr); 1.35-1.14 (m, 2H, cyclopr).

^{13}C NMR (50 MHz, DMSO-d6): δ 187.7, 172.2, 159.2, 159.1, 153.1, 146.8, 146.3, 144.4, 133.3, 131.8, 130.4, 129.2, 126.1, 124.0, 123.2, 122.8, 112.9, 110.2, 107.8, 100.6, 54.7, 30.2, 15.4, 13.5.

HRMS (ESI) calculated for $C_{29}H_{25}N_2O_5S_2$: [M + H]$^+$ 545.12048; found 545.11966

*ethyl4-(2-(1-(benzo[d][1,3]dioxol-5-yl)cyclopropane-1-carboxamido)-4-(4-methoxyphenyl)thiazole-5-carbonyl)benzoate (**9e**)*

Compound **9e** (12.5 mg, 22%) was obtained from ethyl 4-(2-amino-4-(4-methoxyphenyl)thiazole-5-carbonyl)benzoate (38.2 mg, 0.1 mmol) and benzo[1,3]dioxol-5-yl-cyclopropanecarboxylic acid (20.6 mg, 0.1 mmol) in the same manner as described for **9a**.

^{1}H NMR (200 MHz, DMSO-d6): δ 11.89 (s, 1H, broad, NH); 7.63-6.72 (m, 11H, arom); 6.01 (s, 2H, OCH_2O); 4.32 (q, J = 7.2 Hz, 2H, OCH_2); 3.64 (s, 3H, OCH_3); 1.78-1.05 (m, 7H, 3H, CH_3CH_2 + 4H, cyclopr).

^{13}C NMR (50 MHz, DMSO-d6): δ 188.4, 171.9, 166.9, 159.6, 159.2, 153.8, 146.9, 146.3, 138.5, 131.8, 130.4, 128.8, 126.2, 123.2, 121.1, 118.4, 113.0, 112.7, 110.2, 107.8, 100.6, 59.8, 54.7, 30.2, 27.3, 15.4.

HRMS (ESI) calculated for $C_{31}H_{27}N_2O_7S$: [M + H]$^+$ 571.15388; found 571.15322

*N-(5-([1,1′-biphenyl]-4-carbonyl)-4-(4-methoxyphenyl)thiazol-2-yl)-1-(benzo[d][1,3]dioxol-5-yl)cyclopropane-1-carboxamide (**9f**)*

Compound **9f** (13.2 mg, 23%) was obtained from [1,1'-biphenyl]-4-yl(2-amino-4-(4-methoxyphenyl)thiazol-5-yl)methanone (38.0 mg, 0.1 mmol) and benzo[1,3]dioxol-5-yl-cyclopropanecarboxylic acid (20.6 mg, 0.1 mmol) in the same manner as described for **9a**.

^{1}H NMR (200 MHz, DMSO-d6): δ 11.91 (s, 1H, broad, NH); 7.86-6.65 (m, 16H, arom); 6.03 (s, 2H, OCH_2O); 3.64 (s, 3H, OCH_3); 1.62-1.46 (m, 2H, cyclopr); 1.34-1.07 (m, 2H, cyclopr).
^{13}C NMR (50 MHz, DMSO-d6): δ 188.3, 172.3, 159.6, 159.1, 153.9, 146.9, 146.3, 143.3, 138.5, 136.2, 131.8, 130.5, 129.3, 128.6, 127.9, 126.4, 126.2, 125.8, 123.2, 112.8, 110.2, 107.8, 100.6, 54.6, 30.2, 15.5.
HRMS (ESI) calculated for $C_{34}H_{27}N_2O_5S$: $[M + H]^+$ 575.16405; found 575.16323

*1-(benzo[d][1,3]dioxol-5-yl)-N-(4-(4-methoxyphenyl)-5-(4-(pyrrolidin-1-yl)benzoyl)thiazol-2-yl)cyclopropane-1-carboxamide (**9g**)*
Compound **9g** (11.4 mg, 20%) was obtained from (2-amino-4-(4-methoxyphenyl)thiazol-5-yl)(4-(pyrrolidin-1-yl)phenyl)methanone (38.0 mg, 0.1 mmol) and benzo[1,3]dioxol-5-yl-cyclopropanecarboxylic acid (20.6 mg, 0.1 mmol) in the same manner as described for **9a**.
^{1}H NMR (200 MHz, DMSO-d6): δ 11.64 (s, 1H, broad, NH); 7.81-6.37 (m, 11H, arom); 6.04 (s, 2H, OCH_2O); 3.72 (s, 3H, OCH_3); 3.56-3.19 (m, 4H, pyr); 2.18-1.85 (m, 4H, pyr); 1.76-1.12 (m, 6H, 2h pyr + 4H, cyclopr).
^{13}C NMR (50 MHz, DMSO-d6): δ 186.1, 171.9, 158.7, 157.5, 150.4, 149.4, 146.9, 146.3, 131.9, 131.5, 129.7, 126.5, 123.5, 123.2, 122.7, 113.1, 110.3, 107.8, 100.6, 54.7, 46.9, 30.2, 24.4., 15.3.
HRMS (ESI) calculated for $C_{32}H_{30}N_3O_5S$: $[M + H]^+$ 568.19060; found 568.18943

*1-(benzo[d][1,3]dioxol-5-yl)-N-(5-(3-methoxybenzoyl)-4-(4-methoxyphenyl)thiazol-2-yl)cyclopropane-1-carboxamide (**9h**)*
Compound **9h** (22.7 mg, 43%) was obtained from (2-amino-4-(4-methoxyphenyl)thiazol-5-yl)(3-methoxyphenyl)methanone (34.0 mg, 0.1 mmol) and benzo[1,3]dioxol-5-yl-cyclopropanecarboxylic acid (20.6 mg, 0.1 mmol) in the same manner as described for **9a**.
^{1}H NMR (200 MHz, DMSO-d6): δ 11.86 (s, 1H, broad, NH); 7.30-6.61 (m, 11H, arom); 6.01 (s, 2H, OCH_2O); 3.67 (s, 3H, OCH_3); 3.61 (s, 3H, OCH_3); 1.60-1.42 (m, 2H, cyclopr); 1.28-1.04 (m, 2H, cyclopr).
^{13}C NMR (50 MHz, DMSO-d6): δ 188.4, 172.3, 159.7, 159.1, 158.3, 153.9, 146.8, 146.3, 138.6, 131.8, 130.5, 128.9, 126.2, 123.2, 123.1, 121.0, 118.3, 113.0, 112.7, 110.2, 107.8, 100.6, 54.7, 30.2, 15.4.
HRMS (ESI) calculated for $C_{29}H_{25}N_2O_6S$: $[M + H]^+$ 529.14332; found 529.14276

*1-(benzo[d][1,3]dioxol-5-yl)-N-(4-(3-methoxyphenyl)-5-(4-(trifluoromethoxy)benzoyl)thiazol-2-yl)cyclopropane-1-carboxamide (**9j**)*
Compound **9j** (12.0 mg, 21%) was obtained from ((2-amino-4-(3-methoxyphenyl)thiazol-5-yl)(4-(trifluoromethoxy)phenyl)methanone (39.0 mg, 0.1 mmol) and benzo[1,3]dioxol-5-yl-cyclopropanecarboxylic acid ((20.6 mg, 0.1 mmol) in the same manner as described for **9i**.
^{1}H NMR (200 MHz, DMSO-d6): δ 12.00 (s, 1H, broad, NH); 7.74-6.82 (m, 11H, arom); 6.02 (s, 2H, OCH_2O); 3.61 (s, 3H, OCH_3); 1.70-1.44 (m, 2H, cyclopr); 1.42-1.11 (m, 2H, cyclopr).
^{13}C NMR (50 MHz, DMSO-d6): δ 187.6, 172.3, 159.6, 153.5, 146.8, 146.3, 144.6, 140.1, 133.7, 131.8, 131.3, 129.3, 128.3, 126.2, 123.4, 123.2, 122.9, 122.6, 121.1, 118.3, 112.9, 110.2, 107.8, 100.6, 54.7, 30.2, 15.4.
HRMS (ESI) calculated for $C_{29}H_{22}F_3N_2O_6S$: $[M + H]^+$ 583.11506; found 583.11445

*1-(benzo[d][1,3]dioxol-5-yl)-N-(4-(3-methoxyphenyl)-5-(4-(methylthio)benzoyl)thiazol-2-yl)cyclopropane-1-carboxamide (**9k**)*
Compound **9k** (18.5 mg, 34%) was obtained from (2-amino-4-(3-methoxyphenyl)thiazol-5-yl)(4-(methylthio)phenyl)methanone (35.6 mg, 0.1 mmol) and benzo[1,3]dioxol-5-yl-cyclopropanecarboxylic acid (20.6 mg, 0.1 mmol) in the same manner as described for **9i**.
^{1}H NMR (200 MHz, DMSO-d6): δ 11.91 (s, 1H, broad, NH); 7.50-6.73 (m, 11H, arom); 6.01 (s, 2H, OCH_2O); 3.60 (s, 3H, OCH_3); 2.44 (s, 3H, SCH_3); 1.62-1.41 (m, 2H, cyclopr); 1.36-1.10 (m, 2H, cyclopr).

^{13}C NMR (50 MHz, DMSO-d6): δ 187.7, 172.3, 159.3, 158.2, 152.6, 146.8, 146.3, 144.6, 134.9, 133.1, 131.8, 129.2, 128.6, 124.2, 123.2, 121.3, 114.6, 113.9, 110.2, 107.8, 100.6, 54.5, 30.2, 15.4, 13.5.
HRMS (ESI) calculated for $C_{29}H_{25}N_2O_5S_2$: [M + H]+ 545.12048; found 545.11985

*Ethyl4-(2-(1-(benzo[d][1,3]dioxol-5-yl)cyclopropane-1-carboxamido)-4-(3-methoxyphenyl)thiazole-5-carbonyl)benzoate (**9l**)*
Compound **9l** (11.4 mg, 20%) was obtained from ethyl 4-(2-amino-4-(3-methoxyphenyl)thiazole-5-carbonyl)benzoate (38.0 mg, 0.1 mmol) and benzo[1,3]dioxol-5-yl-cyclopropanecarboxylic acid (20.6 mg, 0.1 mmol) in the same manner as described for **9i**.
^{1}H NMR (200 MHz, DMSO-d6): δ 12.02 (s, 1H, broad, NH); 7.70-6.75 (m, 11H, arom); 6.04 (s, 2H, OCH_2O); 4.41 (q, J = 7.4 Hz, 2H, OCH_2); 3.59 (s, 3H, OCH_3); 1.73-1.01 (m, 7H, 3H, CH_3CH_2 + 4H, cyclopr).
^{13}C NMR (50 MHz, DMSO-d6): δ 188.1, 172.2, 167.2, 159.6, 159.2, 152.7, 146.8, 146.3, 138.6, 131.7, 130.5, 128.7, 126.3, 123.4, 123.2, 121.1, 118.3, 113.0, 110.2, 107.8, 100.6, 54.6, 53.3, 30.2, 25.5, 15.4.
HRMS (ESI) calculated for $C_{31}H_{27}N_2O_7S$: $[M + H]^+$ 571.15388; found 571.15304

*N-(5-([1,1′-biphenyl]-4-carbonyl)-4-(3-methoxyphenyl)thiazol-2-yl)-1-(benzo[d][1,3]dioxol-5-yl)cyclopropane-1-carboxamide (**9m**)*
Compound **9m** (12.7 mg, 22%) was obtained from [1,1'-biphenyl]-4-yl(2-amino-4-(3-methoxyphenyl)thiazol-5-yl)methanone (38.6 mg, 0.1 mmol) and benzo[1,3]dioxol-5-yl-cyclopropanecarboxylic acid ((20.6 mg, 0.1 mmol) in the same manner as described for **9i**.
^{1}H NMR (200 MHz, DMSO-d6): δ 11.82 (s, 1H, broad, NH); 7.88-6.69 (m, 16H, arom); 6.01 (s, 2H, OCH_2O); 3.59 (s, 3H, OCH_3); 1.68-1.40 (m, 2H, cyclopr); 1.38-1.12 (m, 2H, cyclopr).
^{13}C NMR (50 MHz, DMSO-d6): δ 188.2, 171.8, 159.7, 159.2, 152.7, 146.9, 146.3, 143.7, 138.4, 136.0, 131.8, 130.6, 129.4, 128.6, 127.9, 126.5, 126.2, 125.9, 123.6, 123.2, 110.2, 107.8, 100.6, 54.7, 30.3, 15.5.
HRMS (ESI) calculated for $C_{34}H_{27}N_2O_5S$: $[M + H]^+$ 575.16405; found 575.16313

*1-(benzo[d][1,3]dioxol-5-yl)-N-(4-(3-methoxyphenyl)-5-(4-(pyrrolidin-1-yl)benzoyl)thiazol-2-yl)cyclopropane-1-carboxamide (**9n**)*
Compound **9n** (11.4 mg, 20%) was obtained from (2-amino-4-(3-methoxyphenyl)thiazol-5-yl)(4-(pyrrolidin-1-yl)phenyl)methanone (38.0 mg, 0.1 mmol) and benzo[1,3]dioxol-5-yl-cyclopropanecarboxylic acid (20.6 mg, 0.1 mmol) in the same manner as described for **9i**.
^{1}H NMR (200 MHz, DMSO-d6): δ 11.78 (s, 1H, broad, NH); 7.80-6.48 (m, 11H, arom); 6.02 (s, 2H, OCH_2O); 3.60 (s, 3H, OCH_3); 3.51-3.15 (m, 4H, pyr); 2.22-1.86 (m, 4H, pyr); 1.72-1.15 (m, 6H, 2H pyr + 4H, cyclopr).
^{13}C NMR (50 MHz, DMSO-d6): δ 186.3, 172.3, 158.7, 157.4, 150.6, 149.4, 146.8, 146.3, 131.8, 131.4, 129.6, 126.5, 123.5, 123.1, 122.7, 113.0, 110.3, 107.8, 100.6, 54.6, 46.8, 30.2, 24.4, 15.3.
HRMS (ESI) calculated for $C_{32}H_{30}N_3O_5S$: $[M + H]^+$ 568.19060; found 568.18983

*1-(benzo[d][1,3]dioxol-5-yl)-N-(5-(3-methoxybenzoyl)-4-(3-methoxyphenyl)thiazol-2-yl)cyclopropane-1-carboxamide (**9o**)*
Compound **9o** (22.3 mg; 42%) was obtained from (2-amino-4-(3-methoxyphenyl)thiazol-5-yl)(3-methoxyphenyl)methanone (34.0 mg, 0.1 mmol) and benzo[1,3]dioxol-5-yl-cyclopropanecarboxylic acid (20.6 mg, 0.1 mmol) in the same manner as described for **9i**.
^{1}H NMR (200 MHz, DMSO-d6): δ 11.93 (s, 1H, broad, NH); 7.28-6.71 (m, 11H, arom); 6.01 (s, 2H, OCH_2O); 3.58 (s, 3H, OCH_3); 3.56 (s, 3H, OCH_3); 1.65-1.41 (m, 2H, cyclopr); 1.38-1.12 (m, 2H, cyclopr).
^{13}C NMR (50 MHz, DMSO-d6): δ 188.5, 172.4, 159.9, 158.2, 153.7, 146.8, 146.3, 138.5, 135.1, 131.7, 128.8, 128.4, 124.6, 123.2, 121.4, 121.0, 118.5, 114.6, 113.9, 112.9, 110.2, 107.8, 100.6, 54.6, 54.5, 30.2, 15.4

HRMS (ESI) calculated for $C_{29}H_{25}N_2O_6S$: $[M + H]^+$ 529.14332; found 529.14270

*1-(benzo[d][1,3]dioxol-5-yl)-N-(4-(2-methoxyphenyl)-5-(4-(trifluoromethoxy)benzoyl)thiazol-2-yl)cyclopropanecarboxamide (**9q**)*
Compound **9q** (15.1 mg, 26 %) was obtained from (2-amino-4-(2-methoxyphenyl)thiazol-5-yl)(4-(trifluoromethoxy)phenyl)methanone (39 mg, 0.1 mmol) and benzo [1,3]dioxol-5-yl-cyclopropanecarboxylic acid (20.6 mg, 0.1 mmol) in the same manner as described for **9p**.
^{1}H NMR (200 MHz, DMSO-d6): δ 11.92 (s, 1H, broad, NH); 7.75-6.91 (m, 11H, arom); 6.03 (s, 2H, OCH_2O); 3.66 (s, 3H, OCH_3); 1.68-1.42 (m, 2H, cyclopr); 1.40-1.13 (m, 2H, cyclopr).
^{13}C NMR (50 MHz, DMSO-d6): δ 187.2, 172.1, 159.5, 153.4, 146.8, 146.3, 144.5, 140.1, 133.6, 131.8, 131.2, 129.3, 128.1, 126.2, 123.3, 123.2, 122.7, 122.6, 121.1, 118.3, 112.9, 110.2, 107.8, 100.6, 54.7, 30.2, 15.5.
HRMS (ESI) calculated for $C_{29}H_{22}F_3N_2O_6S$: $[M + H]^+$ 583.11506; found 583.11478

*1-(benzo[d][1,3]dioxol-5-yl)-N-(5-(3-methoxybenzoyl)-4-(4-(methylthio)phenyl)thiazol-2-yl)cyclopropane-1-carboxamide (**9v**)*
Compound **9v** (13.1 mg, 24%) was obtained from (2-amino-4-(4-(methylthio)phenyl)thiazol-5-yl)(3-methoxyphenyl)methanone (36.0 mg, 0.1 mmol) and benzo[1,3]dioxol-5-yl-cyclopropanecarboxylic acid (20.6 mg, 0.1 mmol) in the same manner as described for **9s**.
^{1}H NMR (200 MHz, DMSO-d6): δ 11.79 (s, 1H, broad, NH); 7.66-6.71 (m, 11H, arom); 6.02 (s, 2H, OCH_2O); 3.60 (s, 3H, OCH_3); 2.44 (s, 3H, SCH_3);1.65-1.42 (m, 2H, cyclopr); 1.40-1.13 (m, 2H, cyclopr).
^{13}C NMR (50 MHz, DMSO-d6): δ 186.5, 171.9, 159.3, 158.1, 151.5, 146.8, 146.3, 135.1, 133.2, 131.9, 129.3, 128.6, 123.2, 121.2, 115.1, 113.0, 112.8, 110.2, 107.8, 100.6, 54.7, 30.2, 15.4, 13.4.
HRMS (ESI) calculated for $C_{29}H_{25}N_2O_5S_2$: $[M + H]^+$ 545.12048; found 545.11990

*1-(benzo[d][1,3]dioxol-5-yl)-N-(5-(3-methoxybenzoyl)-4-(pyridin-3-yl)thiazol-2-yl)cyclopropane-1-carboxamide (**9w**)*
Compound **9w** (12.5 mg, 25%) was obtained from (2-amino-4-(pyridin-3-yl)thiazol-5-yl)(3-methoxyphenyl)methanone (32.0 mg, 0.1 mmol) and benzo[1,3]dioxol-5-yl-cyclopropanecarboxylic acid (20.6 mg, 0.1 mmol) in the same manner as described for **9t**.
^{1}H NMR (200 MHz, DMSO-d6): δ 11.72 (s, 1H, broad, NH); 8.10-7.02 (m, 11H, arom); 6.05 (s, 2H, OCH_2O); 3.62 (s, 3H, OCH_3); 1.77-1.45 (m, 2H, cyclopr); 1.42-1.16 (m, 2H, cyclopr).
^{13}C NMR (50 MHz, DMSO-d6): δ 187.6, 172.1, 159.8, 159.2, 153.1, 149.3, 147.7, 146.3, 138.6, 135.8, 131.8, 129.2, 128.7, 127.9, 123.4, 123.2, 121.3, 118.4, 112.8, 110.2, 107.8, 100.6, 54.7, 30.2, 15.4.
HRMS (ESI) calculated for $C_{27}H_{22}N_3O_5S$: $[M + H]^+$ 500.12801; found 500.12716

*1-(benzo[d][1,3]dioxol-5-yl)-N-(4-(4-chlorophenyl)-5-(3-methoxybenzoyl)thiazol-2-yl)cyclopropane-1-carboxamide (**9x**)*
Compound **9x** (8.7 mg, 16%) was obtained from (2-amino-4-(4-chlorophenyl)thiazol-5-yl)(3-methoxyphenyl)methanone (34.5 mg, 0.1 mmol) and benzo[1,3]dioxol-5-yl-cyclopropanecarboxylic acid (20.6 mg, 0.1 mmol) in the same manner as described for **9u**.
^{1}H NMR (200 MHz, DMSO-d6): δ 11.98 (s, 1H, broad, NH); 7.84-6.85 (m, 11H, arom); 6.02 (s, 2H, OCH_2O); 3.60 (s, 3H, OCH_3); 1.74-1.46 (m, 2H, cyclopr); 1.41-1.15 (m, 2H, cyclopr).
^{13}C NMR (50 MHz, DMSO-d6): δ 186.9, 172.0, 159.8, 152.7, 146.8, 146.3, 143.6, 138.4, 131.7, 129.4, 128.6, 127.9, 126.3, 124.3, 123.2, 121.2, 112.9, 110.2, 107.8, 100.6, 54.6, 30.2, 15.4.
HRMS (ESI) calculated for $C_{28}H_{22}ClN_2O_5S$: [M + H]+ 533.09378; found 533.09371

*Methyl 4-(2-(1-(benzo[d][1,3]dioxol-5-yl)cyclopropane-1-carboxamido)-5-(4-(methylthio)benzoyl)thiazol-4-yl)benzoate (**9y**)*

Compound **9y** (14.3 mg, 25%) was obtained from methyl 4-(2-amino-5-(4-(methylthio)benzoyl)thiazol-4-yl)benzoate (39.0 mg, 0.1 mmol) and benzo[1,3]dioxol-5-yl-cyclopropanecarboxylic acid (20.6 mg, 0.1 mmol) in the same manner as described for **9r**.

^{1}H NMR (200 MHz, DMSO-d6): δ 11.96 (s, 1H, broad, NH); 7.79-6.78 (m, 11H, arom); 6.01 (s, 2H, OCH_2O); 4.12 (s, 3H, CH_3O); 2.41 (s, 3H, SCH_3);1.65-1.42 (m, 2H, cyclopr); 1.39-1.12 (m, 2H, cyclopr).

^{13}C NMR (50 MHz, DMSO-d6): δ 188.7, 172.3, 165.7, 159.7, 159.1, 152.4, 146.8, 146.3, 138.6, 131.8, 129.2, 128.6, 127.8, 126.3, 124.1, 123.2, 121.4, 113.1, 110.2, 107.8, 100.6, 52.8, 30.2, 15.4, 13.5.

HRMS (ESI) calculated for $C_{30}H_{25}N_2O_6S_2$: $[M + H]^+$ 573.11539; found 573.11438

*1-(benzo[d][1,3]dioxol-5-yl)-N-(5-(4-(methylthio)benzoyl)-4-(4-(methylthio)phenyl)thiazol-2-yl)cyclopropanecarboxamide (**9z**)*

Compound **9z** (12 mg, 21%) was obtained from 2-amino-4-(4-(methylthio)phenyl)thiazol-5-yl)(4-(methylthio)phenyl)methanone (37.2 mg, 0.1 mmol) and benzo[1,3]dioxol-5-yl-cyclopropanecarboxylic acid (20.6 mg, 0.1 mmol) in the same manner as described for **9s**.

^{1}H NMR (200 MHz, DMSO-d6): δ 11.89 (s, 1H, broad, NH); 7.64-6.71 (m, 11H, arom); 6.01 (s, 2H, OCH_2O); 2.47 (s, 3H, SCH_3); 2.42 (s, 3H, SCH_3);1.63-1.42 (m, 2H, cyclopr); 1.39-1.14 (m, 2H, cyclopr).

^{13}C NMR (50 MHz, DMSO-d6): δ 187.2, 172.2, 159.8, 159.2, 153.1, 146.8, 146.3, 138.5, 131.9, 129.3, 128.7, 127.9, 124.2, 123.2, 121.3, 118.4, 112.8, 110.2, 107.8, 100.6, 30.2, 15.4, 13.5.

HRMS (ESI) calculated for $C_{29}H_{25}N_2O_4S_3$: [M + H]+ 561.09763; found 561.09670

2-amino-4-phenylthiazol-5-yl(phenyl)methanone (11)

2-bromo-1, 3- diphenylpropano-1, 3-dione 10 (300.3 mg, 1 mmol) and thiourea (77 mg, 1 mmol) in anhydrous EtOH (2 ml) was stirred at reflux until the reaction was judged complete (about 2 h) (HPLC-MS).

The solvent was concentrated under reduced pressure and the mixture partitioned between dichloromethane and H_2O. The organic layer was washed with brine and dried over anhydrous Na_2SO_4.The organic phases were concentrated in vacuum and the crude product, verified by HPLC and MS was purified with preparative HPLC to give the final product as white solid with purity higher than 95% ,as confirmed by HPLC-MS (211 mg, 75%).

^{1}H NMR (200 MHz, DMSO-d6) δ 7.98 (s, 2H,NH_2);7.42-7.35 (m, 2H,arom); 7.32-7.26 (m, 1H,arom); 7.23 (m,2H,arom); 7.17 (m, 3H,arom); 7.11 (m, 2H,arom).

^{13}C NMR (75 MHz, DMSO-d6) d 188.5, 170.9, 157.9, 138.5, 133.8, 131.7, 129.6, 128.5, 128.3, 127.7, 127.3, 122.5.

HRMS (ESI) calculated for $C_{16}H_{13}N_2OS$ $[M + H]^+$ 281.0743, found 281.0761.

2-(benzo[d][1,3]dioxol-5-yl)-N-(5-benzoyl-4-phenylthiazol-2-yl)acetamide(12b)

2-(benzo[d][1,3]dioxol-5-yl)acetic acid (18 mg, 0.1 mmol), DIPEA (38 µL, 0.1 mmol) and HATU (42 mg, 0.1 mmol) were dissolved in in anhydrous DMF (1 mL). Then, to the solution was added 2-amino-(4-phenylthiazol-5-yl) phenylmethanone 11 (28 mg, 0.1 mmol). The reaction was heated to 50 °C for 15 hours. The purification of the final product was performed by preparative HPLC to obtain the title compound as pale-yellow oil with purity of >95% as confirmed by HPLC-MS (20 mg, 45%).

^{1}H NMR (200 MHz, DMSO-d6): δ 11.87 (s, 1H, broad, NH); 7.81-6.84 (m, 13H, arom); 6.00 (s, 2H, OCH2O); 3.12 (s, 2H, ArCH2C(O)).

^{13}C NMR (50 MHz, DMSO-d6): δ 188.4, 171.5, 159.7, 158.3, 152.7, 146.8, 146.3, 138.5, 131.8, 129.3, 128.7, 127.9, 124.3, 123.2, 121.3, 118.5, 113.1, 110.3, 107.8, 100.6, 41.7.

HRMS (ESI) calculated for $C_{25}H_{19}N_2O_4S$: [M + H]+ 443.10654; found 443.10554

3-(benzo[d][1,3]dioxol-5-yl)-N-(5-benzoyl-4-phenylthiazol-2-yl)-2-methylpropanamide (13b)

3-(benzo[d][1,3]dioxol-5-yl)-2-methylpropanoic acid (21 mg, 0.1 mmol) was dissolved in 1 mL of anhydrous DMF with HATU (38 mg, 0.1 mmol) and DIPEA (38 µL, 0.1 mmol). The reaction was stirred at room temperature and after 5 min and 2-amino-(4-phenylthiazol-5-yl) phenylmethanone 11 (28 mg, 0.1 mmol) was added. The solution was heated to 50 °C until completeness (about 20 h). The purification of the final product was performed by preparative HPLC. The title compound, afforded as pale-yellow oil, had a purity of >95% as confirmed by HPLC-MS (21 mg, 45%).

^{1}H NMR (200 MHz, DMSO-d6): δ 11.98 (s, 1H, broad, NH); 7.82-6.79 (m, 13H, arom); 6.01 (s, 2H, OCH2O); 2.96 (m, 1H, CH); 2.78 (d, J = 6.2 Hz, 2H, ArCH2); 1.18 (d, 3H, J = 6.8 Hz, CH3).

^{13}C NMR (50 MHz, DMSO-d6): δ 187.6, 171.3, 159.7, 159.1, 153.1, 146.8, 146.3, 138.4, 131.8, 129.2, 128.7, 127.9, 124.2, 123.2, 121.2, 118.4, 112.8, 110.2, 107.8, 100.6, 40.4, 36.8, 16.1.

HRMS (ESI) calculated for $C_{27}H_{23}N_2O_4S$: [M + H]+ 471.13784; found 471.13753

(Z)-3-(benzo[d][1,3]dioxol-5-yl)-N-(5-benzoyl-4-phenylthiazol-2-yl)acrylamide (14b)

((Z)-3-(benzo[d][1,3]dioxol-5-yl)acrylic acid (19 mg, 0.1 mmol), HATU (38 mg, 0.1 mmol) and DIPEA (38 µL, 0.1 mmol) were dissolved in anhydrous DMF (1 mL) and stirred for 5 min. Then, 2-amino-(4-phenylthiazol-5-yl) phenylmethanone 11 (28 mg, 0.1 mmol) was added. The mixture was heated to 50 °C overnight and then purified by preparative HPLC. The final product was afforded as a pale brown oil with purity of >95% as confirmed by HPLC-MS (18 mg, 40%).

^{1}H NMR (200 MHz, DMSO-d6): δ 12.02 (s, 1H, broad, NH); 7.86-6.75 (m, 13H, arom); 6.34 (d, J = 8.6 Hz, H, CH=); 6.22 (d, J = 8.6 Hz, H, CH=); 6.05 (s, 2H, OCH2O).

^{13}C NMR (50 MHz, DMSO-d6): δ 188.1, 172.1, 159.7, 158.0, 153.2, 146.8, 146.3, 144.3, 138.4, 131.8, 129.3, 128.7, 127.9, 127.7, 124.1, 123.2, 121.2, 118.5, 112.8, 110.2, 107.8, 100.6.

HRMS (ESI) calculated for $C_{26}H_{18}N_2O_4S$: [M + H]+ 454.09872; found 454.09772

2-(benzo[d][1,3]dioxol-5-yloxy)-N-(5-benzoyl-4-phenylthiazol-2-yl)-2-methylpropanamide (17)

Benzo[d][1,3]dioxol-5-ol 15 (138.0 mg, 1 mmol) and K_2CO_3 (414 mg, 3 mmol) were dissolved in DMF (1 mL). The solution was stirred for 10 min at room temperature and then ethyl 2-bromo-2-methylpropanoate (176 µl, 1.2 mmol) was added. The resulting reaction mixture was stirred at room temperature for 16 h. The residue was taken up in EtOAc and washed with H_2O and brine, dried over Na_2SO_4, and concentrated under reduced pressure to afford the product, ethyl 2-(benzo[d][1,3]dioxol-5-yloxy)-2-methylpropanoate, as a yellow oil (176.0 mg, 70%).

Ethyl 2-(benzo[d][1,3]dioxol-5-yloxy)-2-methylpropanoate was dissolved in ACN (2 mL) and KOH 6N was added drop-wise (1 mL). The reaction was stirred at T= 60 °C until complete hydrolysis (about 3h).

The mixture was then cooled to room temperature and concentrated under reduced pressure. The residue was taken up in H_2O, acidified with 1N HCl and extracted with EtOAc / $NaHCO_3$ 1N (3 x 3 mL). The combined organic layers were washed with brine, dried over Na_2SO_4, and concentrated under reduced pressure to afford 2-(benzo[d][1,3]dioxol-5-yloxy)-2-methylpropanoic acid 16 (136.0 mg, 61%).

To a solution of 2-(benzo[d][1,3]dioxol-5-yloxy)-2-methylpropanoic acid (33.6 mg, 0.15 mmol) in DMF, HATU (51.3 mg, 0.15 mmol) and DIPEA (57.7 µL, 0.15 mmol) were added and stirred for 5 minutes at room temperature. Then, 2-amino-(4-phenylthiazol-5-yl) phenylmethanone 11 (42 mg, 0.15 mmol) was added and the resulting mixture stirred to T = 50°C for 24 h.

The final product was purified by preparative HPLC, and the peak of interest was concentrated under vacuum to obtain 2-(benzo[d][1,3]dioxol-5-yloxy)-N-(5-benzoyl-4-phenylthiazol-2-yl)-2-methylpropanamide as a yellow powder, with purity of >95% as determined by HPLC-MS (15.9 mg, 22%).

^{1}H NMR (200 MHz, DMSO-d6): δ 11.89 (s, 1H, broad, NH); 7.87-6.73 (m, 17H, arom); 6.02 (s, 2H, OCH2O); 1.22 (s, 6H, 2CH3).
^{13}C NMR (50 MHz, DMSO-d6): δ 186.8, 171.9, 159.7, 159.1, 152.8, 146.8, 146.3, 143.2, 138.5, 135.6, 132.6, 131.8, 130.6, 129.2, 128.7, 127.9, 127.3, 126.7, 125.7, 124.2, 123.2, 121.2, 118.6, 112.9, 110.2, 107.8, 100.6, 58.2, 22.5.
HRMS (ESI) calculated for $C_{27}H_{23}N_2O_5S$: [M + H]+ 487.13276; found 487.13252

N-(5-benzoyl-4-phenylthiazol-2-yl)-1-(4-chlorophenyl)cyclopropanecarboxamide(18)
A mixture of 1-(4-chlorophenyl)cyclopropanecarboxylic acid (21.0 mg, 0.1 mmol), HATU (38 mg, 0.1 mmol) and DIPEA (38 μL, 0.1 mmol) was dissolved in anhydrous DMF (1 mL) and stirred for few minutes. To the solution 2-amino-(4-phenylthiazol-5-yl) phenylmethanone 11 (28 mg, 0.1 mmol) was added. The reaction was stirred at 50 °C for 36 hours. After completeness, the mixture was purified by using preparative HPLC. The title compound was obtained as pale brown oil with purity of >95% confirmed by HPLC-MS (23 mg, 50%).
^{1}H NMR (200 MHz, DMSO-d6): δ 11.98 (s, 1H, broad, NH); 7.88-6.79 (m, 13H, arom); 1.73-1.45 (m, 2H, cyclopr); 1.43-1.16 (m, 2H, cyclopr).
^{13}C NMR (50 MHz, DMSO-d6): δ 188.2, 171.8, 159.7, 153.1, 146.8, 146.3, 138.5, 131.6, 129.2, 128.7, 127.8, 124.2, 123.1, 121.3, 118.4, 113.0, 110.2, 107.8, 100.6, 30.2, 15.5.
HRMS (ESI) calculated for $C_{26}H_{20}ClN_2O_2S$: [M + H]+ 459.09339; found 459.09273
